# Functionally diverse microbial communities show resilience in response to a record-breaking rain event

**DOI:** 10.1038/s43705-022-00162-z

**Published:** 2022-09-02

**Authors:** Jordan R. Walker, Alaina C. Woods, Mary K. Pierce, Jamie L. Steichen, Antonietta Quigg, Karl Kaiser, Jessica M. Labonté

**Affiliations:** 1 Department of Marine Biology, Texas A&M University at Galveston, Galveston, TX USA; 2 Department of Marine and Coastal Environmental Sciences, Texas A&M University at Galveston, Galveston, TX USA; 3 Department of Oceanography, Texas A&M University, College Station, TX USA

**Keywords:** Metagenomics, Microbial ecology

## Abstract

Estuaries provide many ecosystem services and host a majority of the world’s population. Here, the response of microbial communities after a record-breaking flood event in a highly urbanized estuary was followed. Hurricane Harvey (hereafter Harvey) was a category 4 hurricane that made landfall on the Texas coast in 2017 and lashed the Houston area with 1.4–1.7 × 10^10^ m^3^ of rainfall, disrupting the natural gradients of nutrients and salinity. Here, we utilized metagenomics to analyze how Harvey altered the microbial community of Galveston Bay over five weeks following the storm. We hypothesized that the community would shift from a marine dominated community to that of a terrestrial and freshwater origin. We found that following the storm there were changes in the distribution of species with specific metabolic capacities, such as Cyanobacteria, enriched in oxygenic photosynthesis and nitrogen fixation genes, as well as Verrucomicrobia and Betaproteobacteria, with high prevalence of the SOX complex and anoxygenic photosynthesis genes. On the other hand, dominant members of the community with more diverse metabolic capabilities showed less fluctuations in their distribution. Our results highlight how massive precipitation disturbances can alter microbial communities and how the coalescence of diverse microorganisms creates a resilient community able to maintain ecosystem services even when the system is in an altered state.

## Introduction

Pulse disturbances, specifically related to precipitation, have garnered increased attention due to the predictions of their increased frequency and strength [1–3]. Models are predicting that by 2070, 99% of the world’s land mass will see increased frequency of extreme weather events [4]. Projections include increased frequencies of flooding from precipitation alone as well as compound flooding from meteorological tides and precipitation in coastal communities [4, 5]. Coastal communities will also be at higher risk of hurricanes which are projected to increase in both intensity and frequency [6]. As a result, it is important to understand how the biological functions of ecosystems are impacted following disturbances from extreme precipitation. Microbial communities control many ecosystem functions through the uptake and digestion of inorganic and organic matter that is transformed and made available to successive trophic levels. The abrupt changes in the water quality of coastal ecosystems following a hurricane (decreased water temperature, salinity, and dissolved oxygen, and increased sediment loading) have been shown to affect the resident microbial communities [7–10]. Similarly, the altered state of the microbial communities following the passing of Hurricanes Katrina and Rita were attributed to higher concentrations and availability of nutrients such as ammonium, nitrate, and nitrite [11]. Analysis of the 16S rRNA genes in Galveston Bay (Texas, USA) following Harvey, showed that microbial communities changed from mainly estuarine to freshwater enriched, before starting to switch back to a more estuarine/marine community [10].

Microbial communities can be highly resilient and can quickly return to their original composition after being disturbed [12, 13]. Microbial resiliency in aquatic environments has been linked to characteristics unique to both the microorganisms and the environment, including dormancy, speciation rates, horizontal gene transfer, water residence times, and distance from inflows [14, 15]. One proposed explanation for community resilience is that there are dominant “core” taxonomic groups of microbes that can retain similar composition and function despite changes in the system [16]. Mixing has been shown to be a source of resilience, which can drive the microbial communities in the epilimnion and hypolimnion layers of a freshwater lake to pre-disturbance conditions in seven and eleven days, respectively [17].

Estuaries act as a buffer for the flow of runoff from freshwater rivers to pelagic ecosystems. This buffer can be particularly important in coastal systems with large anthropogenic influences that can be sources of excessive nutrients and pollutants. With nutrient-rich waters and high rates of primary productivity, microbial communities in estuaries can respire substantial amounts of organic matter and transform nutrients and pollutants brought in from runoff [18, 19]. These processes are important in Galveston Bay given that the watershed includes both the Houston and Dallas-Fort Worth metroplexes. Galveston Bay is frequently affected by oil and chemical releases [20], and the surrounding counties (Chambers, Harris, and Montgomery) contain 28 state and federal superfund sites and over 500,000 acres of farmland [21–23]. In addition, storms can provide a pulse of nutrients stimulating opportunistic algal species leading to harmful algal blooms (HABs) [18, 24, 25]. HABs can lead to hypoxia/anoxia and have consequences at higher levels of the food chain as has been reported historically in Galveston Bay [18, 24, 26]. Thus, it is important to understand how pulse disturbances change Galveston Bay microbial populations and the services they provide.

Harvey made landfall in Port Aransas, TX, on the 25th of August 2017 as a Category 4 storm. Galveston, TX, located ~280 km north-east of Port Aransas, did not experience hurricane-force winds but experienced a tropical storm with winds between 60–120 km/h and a storm surge of <1 m. Moreover, Harvey stalled over Houston and Galveston Bay, releasing 1.4–1.7 × 10^10^ m^3^ of rainfall, or an average of 120–150 cm of cumulated rainfall, over the next four days (August 25–29, 2017) [7], making Harvey the highest-volume rainfall event ever recorded in the United States. Floodwater, storm water, and rainwater all flushed into Galveston Bay, causing a drop in salinity, temperature, and dissolved oxygen [7, 10, 27]. Moreover, 9.86 × 10^7^ metric tons of sediment were resuspended into Galveston Bay along with increases in dissolved organic matter (DOM), nutrients, and turbidity [7, 28]

Here, we examined the impact of this record-breaking disturbance on the resilience of the microbial communities within Galveston Bay. We sampled a transect in Galveston Bay four times over five weeks following the storm and used metagenomics to identify the changes in the taxonomic diversity and metabolic potential of the microbial communities. We hypothesized (i) an increase in microbes associated with sediment and terrestrial environments due to storm and flood waters; (ii) an increase in heterotrophic microbes able to degrade complex carbon compounds from the sediment resuspension; and (iii) a return to estuarine microbes as the environmental conditions reverted back to those before the storm.

## Material and methods

### Sample collection and processing

**Fig. 1:**
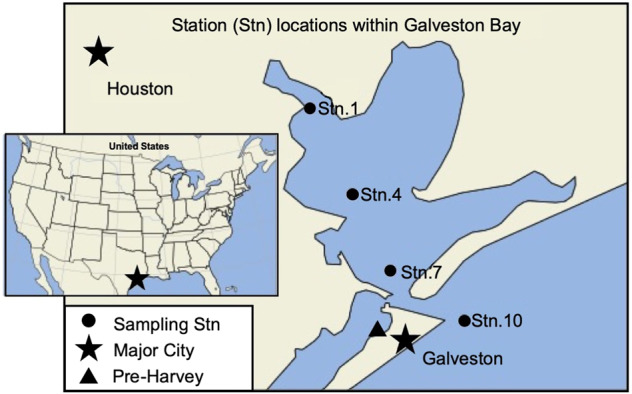
Sampling map of Galveston Bay, Texas (United States) showing the sampling stations (Stn) used during each of the sampling efforts. The triangle represents the Texas A&M University at Galveston boat basin where pre-Harvey samples were taken on July 31 and August 22 of 2017. Sampling trips always started in the morning and transited from Station 1 to Station 10 from the mouth of the San Jacinto River to the Gulf of Mexico.

Sampling campaigns started when safe sailing was possible, one week after Harvey, and ended when the environmental parameters returned to pre-storm conditions. Samples were collected on September 4, 9, 16 and 28, 2017 onboard the Texas A&M University at Galveston’s (TAMUG) R/V Trident along a transect sampled as a subset of a larger effort (Fig. 1). Samples were collected at Station 1 (29°67 N, 94°97 W), Station 4 (29°53 N, 94°89 W), Station 7 (29°41 N, 94°82 W) and Station 10 (29°33 N, 94.68 W). Sampling trips started in the morning and were always transiting from Station 1 to Station 10. The locations chosen represent a gradient from the freshwater inflow of the San Jacinto River to the marine Gulf of Mexico along the Houston Ship Channel. Fortuitously, samples were collected from TAMUG’s boat basin (29°32 N, 94°85 W) on July 31 and August 22, 2017 (see Fig. 1); this is geographically closest to Station 7, but the environmental conditions are more closely related to those at Station 10 (Fig. 1; Supplementary Table 1). Because of the possible differences in environmental parameters, we will compare the post-Harvey samples to the pre-Harvey samples, but will refrain from making assumptions regarding ecosystem recovery. Surface water (4–25 L) was collected using a 5-gallon bucket and pre-filtered onboard immediately (Nitex filter, 30 μm) to remove small grazers and large particles. Samples were then stored on the boat in the dark at room temperature for up to four hours in HDPE carboys and brought to the laboratory, where they were stored at 4 °C and filtered on the same day as sampling. Generally, each sample was filtered through a 142 mm glass fiber (GF) filters followed by a 0.22 µm pore-size polyvinylidene fluoride (PVDF) filters; pore sizes varied due to availability of supplies (Supplementary Table 2). All GF and PVDF filters were stored at −20 °C.

### DNA extraction and sequencing

Total DNA was extracted from an aseptically cut fraction of the filters (corresponding to a water volume of ~4 L; Supplementary Table 2) using a standard phenol chloroform extraction protocol [29], where the lysis buffer consisted of 400 mM NaCl, 750 mM sucrose, 20 mM EDTA, and 50 mM Tris-HCl, pH = 9.0. Between 0.5–1 µg of DNA per sample was sequenced using Illumina HiSeq 4000 (150 bp paired-end) technologies at the Texas A&M Genomics and Bioinformatics facility (College Station, TX, United States).

### Whole community metagenomic analysis

A custom bioinformatics pipeline was utilized to perform quality control (BBtools), de novo assembly MEGAHIT [30], gene prediction and annotation (Prodigal [31] and DIAMOND [32], whole community analysis (MEGAN 6 Ultimate Edition [33], and visualization (R [34] and python matplotlib [35] and Seaborn [36] as detailed in the Supplementary Methods. Reads from the glass fiber and PVDF fractions were combined by each specific sample date and location and processed through the pipeline as a single sample, for a total of 18 metagenomes. Ribosomal RNA sequences were recovered from the metagenomic raw reads using phyloFlash [37].

### Binning and analysis of metagenome assembled genomes (MAGs)

Binning and MAG analysis was performed by taking the merged and unmerged reads from BBtools output, in the community analysis, and performing one de novo co-assembly (MEGAHIT [30]). Reads were mapped back to the co-assembly (BWA [38]), a binning algorithm was applied (MetaBat2 [39]), binning quality was assessed (CheckM [40]), taxonomy was assigned (Kraken2 [41] and CheckM [40]), gene prediction and annotation was performed (Prodigal [31] and GhostKoala [42]), and results were visualized (Anvi’o [43], python matplotlib [35] and Seaborn [36], R [34]) as detailed in the Supplementary Methods.

### Statistical analyses

Abundance and depth information were normalized to each metagenome using the total amount of genes predicted by Prodigal. Shannon index, Pielou’s evenness, and Chao1 index calculations were conducted using the R package vegan [44]. For the calculation of the aforementioned metrics, MAGs were considered species and were assigned a 0 value if below 1 × 10^−4^ percent of the community. Testing for statistical difference was done using Kruskal-Wallis and subsequently Dunn’s test, tests were performed grouping by sampling date as well as grouping by station. NMDS plots were constructed using each MAG’s relative abundance, at the class level for the total gene pool, and the environmental variables reported in Steichen et al. Relative abundances were square-root transformed and the metadata was *z*-score transformed prior to analysis. KEGG functions, determined using MEGAN6, were normalized to Prodigal total gene counts, and *z*-score transformed across metagenomes. KEGG functions in the MAGs were extracted from Anvi’o and were assigned to KEGG modules, which represent functional units of gene and reaction sets manually curated by KEGG. Only KEGG modules containing more than 50% completion were considered. The best fit distance matrix for the total gene pool, MAGs, and KEGG functions in the total gene pool was determined using the rankindex() function. We then found the environmental variables with the maximum rank correlation to each dissimilarity using the bioenv() function from vegan. We performed an analysis of similarity between sampling date and stations using the anosim() function in vegan. NMDS analyses of the datasets were compared to NMDS analyses of the environmental data using procrustes and protest in vegan [44].

### Calculation of metabolic overlap

Metabolic overlap was calculated as detailed in Hester et al. [45] using the KEGG annotations (details in the Supplementary Methods).

## Results

Prior to the storm, we had collected samples adjacent to Station 7 (Fig. 1, Supplementary Table 1); afterwards we sampled four times along a transect from the San Jacinto River to the mouth of the Gulf of Mexico. Herein we focus on comparisons of pre-Harvey samples and Station 7, but also compare between stations. For the 18 microbial metagenomes, on average each metagenome had ~360 Mbp, ~32 M reads, and an N50 of 753 bp (Supplementary Table 3). Binning of the co-assembly resulted in the creation of 1395 metagenome assembled genomes (MAGS), of which 103 were high quality MAGs (>90% complete, <5% contamination) and 349 MAGS were medium quality (>50% complete, <10% contamination), not including high quality draft genomes (Supplementary Fig. 1A). In our analyses, we only discuss the high and medium quality MAGs, unless indicated otherwise.

### Runoff from Harvey changed the composition of Galveston Bay’s microbial community

**Fig. 2:**
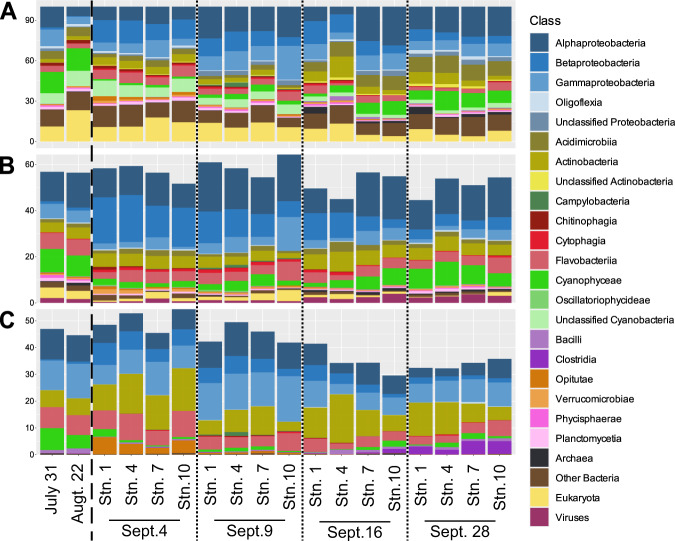
Changes in the microbial community composition of Galveston Bay following Hurricane Harvey. Stacked bar histogram showing the relative abundance of major microbial groups at the class level identified by 16S and 18S rRNA extracted from the metagenomic data (**A**), the complete gene pool (**B**), and the MAGs generated from the co-assembly (**C**). The July 31 and August 22 samples were taken before Hurricane Harvey.

We compared the small subunit ribosomal RNA (SSU rRNA) gene sequences identified in the metagenomes to the taxonomic assignment of the predicted proteins (against NCBI nr database; proxy to look at the whole microbial community via the gene pool characterization) and the taxonomic assignment of the MAGs (with Kraken2; proxy for the dominant members of the microbial community) (Fig. 2). Even with the inherent differences in the types and amount of data, the taxonomic composition of the metagenomes largely agreed at the class level, whether we were comparing the taxonomy of the SSU rRNA genes (Fig. 2A), total predicted proteins (Fig. 2B), or MAGs (Fig. 2C).

Changes were observed for some members of the microbial community, including Proteobacteria, Cyanophyaceae, and Verrucomicrobiae (Fig. 2). Proteobacteria was the most prevalent phylum in all analyses. Proteobacteria’s relative abundance was 15% higher on Sept. 4 than before the storm (Fig. 2A, B). Betaproteobacteria accounted for most of this increase with 16S rRNA relative abundances increasing from 2% to 12% and the total gene pool relative abundances increasing from 1% to 14% (Fig. 2A, B). Alphaproteobacteria relative abundances did not change following the storm, but the relative abundance of Alphaproteobacterial 16S rRNA genes spiked on Sept. 16 to 25% from 13% and remained elevated at 22% until Sept. 28. On Sept. 28, a smaller spike in Alphaproteobacterial genes was seen in the total gene pool (Fig. 2A, B). Cyanophyaceae decreased in both the 16S rRNA and total gene pool, from relative abundances of 16% prior to the storm to 2% on Sept. 4 and from 10% to 2%, respectively (Fig. 2A, B). Cyanophyaceae levels returned to 11% of the 16S rRNA genes and 8% of the gene pool by Sept. 28 (Fig. 2A, B). Considering all Cyanobacteria-related 16S rRNA genes, which include unidentifiable bacteria and chloroplasts, the decrease is starker as relative abundances dropped from 26% to 9% on Sept. 4 and returned to just 13% by Sept. 28. Verrucomicrobia decreased in relative abundance by half on Sept. 16 in both analyses, but minimal changes were observed in the first two samplings (Fig. 2A, B). The 16S rRNA revealed that the Verrucomicrobia classes Opitutae and Methylacidiphilae relative abundances increased from 0.3% to 1.5% and 0% to 0.3%, respectively, on Sept. 4 while Spartobacteria decreased from 1% to 0.2%. Only the classes Opitutae and Verrucomicrobia were found in the total gene pool and all changes within the phylum were attributable to Opitutae. On average 11% of the SSU rRNA genes were identified as eukaryotic; however, in the total gene pool we found that on average <1% of the genes within each metagenome were identified as eukaryotic and none of the MAGs generated were classified as eukaryotic (Fig. 2). Upon inspection of the functional potential of the total gene pool, we found that only 6% of the all photosynthetic genes found in the metagenomes were classified as eukaryotic.

The majority of high quality (77%) and medium quality (61%) MAGS were from the Flavobacteria, Alphaproteobacteria, and Gammaprotebacteria classes (Supplementary Fig. 1B–D). A large fraction of the MAGs, 21% of the medium quality and 4% of the high-quality, were from Actinobacteria (Supplementary Fig. 1C, D). Proteobacteria MAGs relative abundances were not directly affected following the storm; however, by Sept. 28 Proteobacteria had declined by 8% of the total community (Fig. 1C). Alphaproteobacteria and Gammaprotebacteria were the main contributors to the declines seen in Proteobacteria. Betaproteobacteria increased from 0.5% of the community to over 4% on Sept. 4 before dropping to pre-Harvey levels by Sept. 28 (Fig. 1C). Of the small subunit rRNA genes in Betaproteobacteria that had hits to known species, four were gut associated, MAGs #342, #1220, #623, and #836 (two *Sutterella* sp. MAGs, *Burholderia contaminans*, and *Burkholderia multivorans*), and two were associated with soil, MAGs #160 and #396 (*Hydrophaga* sp., *Ramlibacter tatouinensis*) (Supplementary Table 4). Cyanophyaceae MAGs relative abundance was 7% before the storm and fell to 0.4% on Sept. 4, then steadily increased to 2% by Sept. 28 (Fig. 1C). Opitutae MAGs relative abundance was barely detectable (~0.001%) in pre-Harvey samples, rose to 2% on Sept. 4, then declined the following weeks to 0.02% on Sept. 28 (Fig. 1C). MAG #625 was the only MAG containing a SSU rRNA identified as Verrucomicrobia, which shared 100% identity with the SSU rRNA gene of the obligate anaerobe *Opitutus terrae* PB90–1 (Supplementary Table 4).

**Fig. 3:**
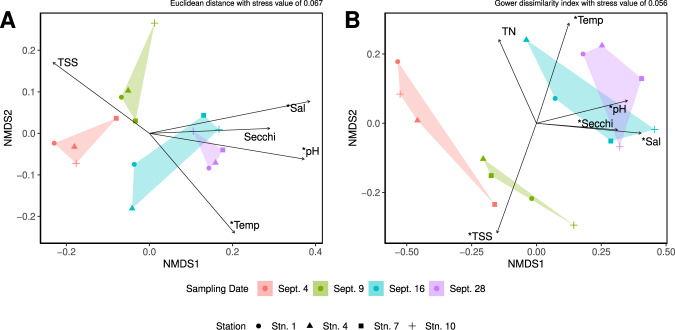
NMDS showing that samples grouped by collection date rather than geographic location within Galveston Bay. NMDS from the complete gene pool (**A**) and the MAGs (**B**), with stress in both plots of type 1, with 2 dimensions, (**A**) had a stress value of 0.067 and (**B**) of 0.056. Dissimilarity indices were assessed via the rankindex function in vegan the euclidean index was used in (**A**) while the gower index was used in (**B**). Significant environmental conditions (α < 0.10) are shown as vectors the lengths of which have been halved in order to better visualize the groupings. Significant vectors with an α < 0.05 are denoted with *.

NMDS analyses showed that samples significantly grouped by collection date rather than by their geographic location within Galveston Bay for both the total gene pool (Fig. 3A) and MAG analyses (Fig. 3B). These results were corroborated by the analysis of similarity which significantly correlated with sampling date (R > 0.60, *p* < 0.05) while grouping by stations returned negative R values, indicating that when grouped by stations the dissimilarity was highest within the groups. Three of the tested abiotic factors (salinity, pH, and temperature) significantly correlated (*p* < 0.05) with the changes on the community composition for both the total gene pool and MAGs. The results of the bioenv() test supported these three parameters as having the maximum rank correlation with the MAG dissimilarity. Turbidity (Secchi disc depth) and total suspended sediments were significant when comparing the MAGs (*p* = 0.05), but to a lesser degree when comparing the total gene pool (*p* = 0.1). Salinity, pH, temperature, and total suspended sediments were the parameters with the highest rank correlation to the total gene pool dissimilarity. Total nitrogen was significantly correlated to the MAG analysis at a confidence value of less than 0.1. Procrustes results showed that the NMDS analyses of the total gene pool and the MAGs did correlate (ms12 > 0.7, *p* < 0.05) with the NMDS of the environmental parameters. We also evaluated the microbial diversity, evenness, and richness from each metagenome using Simpson’s, Shannon’s, Pielou’s, and Chao1 indices. The only metric that showed a significant difference (α < 0.05) was the Simpson index measured for MAGs, and the Dunn’s Test revealed the only significant difference (α < 0.05) was between Sept. 9 and Sept. 28 (Supplementary Fig. 2). In addition to little differentiation in the indices, trends in the different indices were dependent on the type of analysis performed (Supplementary Fig. 2).

**Fig. 4:**
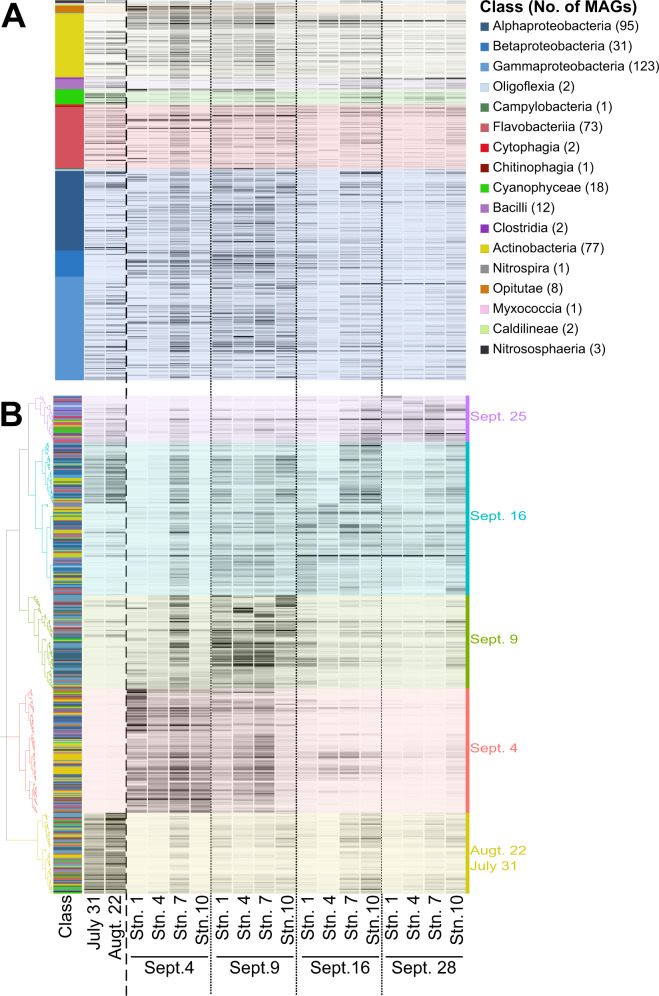
Heat map showing the square root transformed MAG percent community as calculated by CheckM. The MAGs were grouped according to taxonomic classification (**A**) or hierarchical clustering of the MAG percent community (**B**). Each row represents a high or medium quality MAG and the intensity of the bands represent their prevalence in each metagenome (columns). Color bars on the far left and background shading in (**A**) correspond to the class of the MAG as determined by Kraken2.

Hierarchical clustering of the MAGs revealed distinct groupings based on sampling date (Fig. 4B). Alphaproteobacteria and Gammaproteobacteria had MAGs that were representing relatively high percentages of the community throughout all sampling clusters (Fig. 4B). Cyanobacteria MAGs were most prevalent in clusters associated with pre-Harvey and after Sept. 16, except for four MAGs (MAG #3, #98, #617, and #864) (Fig. 4B). All Cyanobacteria MAGs in the two weeks following Harvey that had identifiable small subunit rRNA genes were from freshwater or terrestrial origins (Supplementary Table 4, Fig. 4B). Opitutae MAGs were below detection levels, except in clusters associated with Sept. 4 and Sept. 9 (Fig. 4B). All the MAGs that clustered together on Sept. 4 were largely undetectable in the other sampling date clusters (Fig. 4B).

**Fig. 5:**
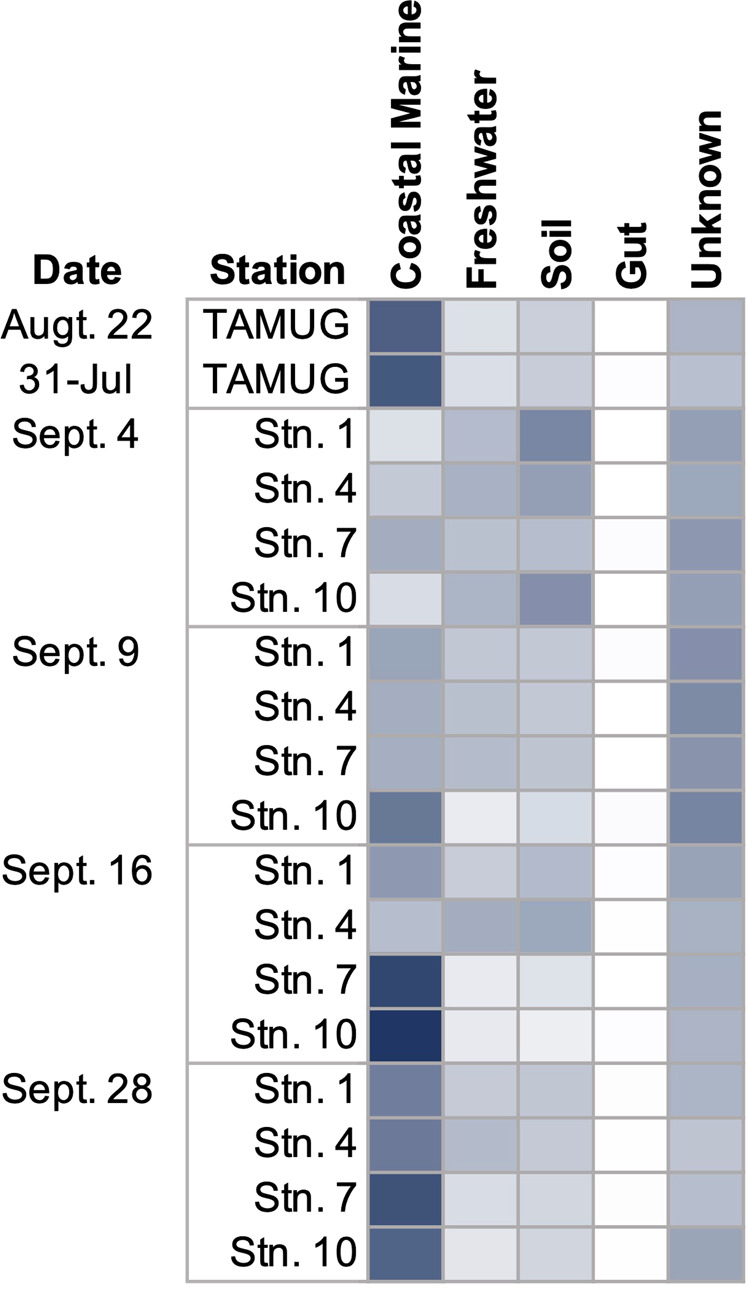
The estimated proportion of each source environment contributing to each metagenome as determined using mSourcetracker. The sources used in mSourcetracker include coastal marine metagenomes from Ocean Sampling Day (2014), freshwater metagenomes from the Solimões river, soil metagenomes from Australia, human gut metagenomes. Unknown stands for sequences of undetermined origin.

We determined the origin of the microbial communities within each sample by utilizing mSourceTracker to compare the taxonomic composition of several environments from urbanized coastal systems to our samples. Prior to Harvey the microbial community had a 50% contribution from coastal or marine sources, 10% from freshwater, and 15% from soil (Fig. 5). Following the storm on Sept. 4 and Sept. 9, the contribution of coastal or marine sources was reduced by half, freshwater contributions increased the most followed by soil and unknown sources (Fig. 5). By Sept. 16, the contributions of sources to the community had shifted back to primarily coastal and marine sources. Additionally, in the weeks of Sept. 16 and Sept. 28, freshwater was more of a contributor to Stations 1 and 4 than Stations 7 and 10 (Fig. 5), reflective of the Galveston Bay estuarine gradient.

### Harvey changed the microbial genomic potential

**Fig. 6:**
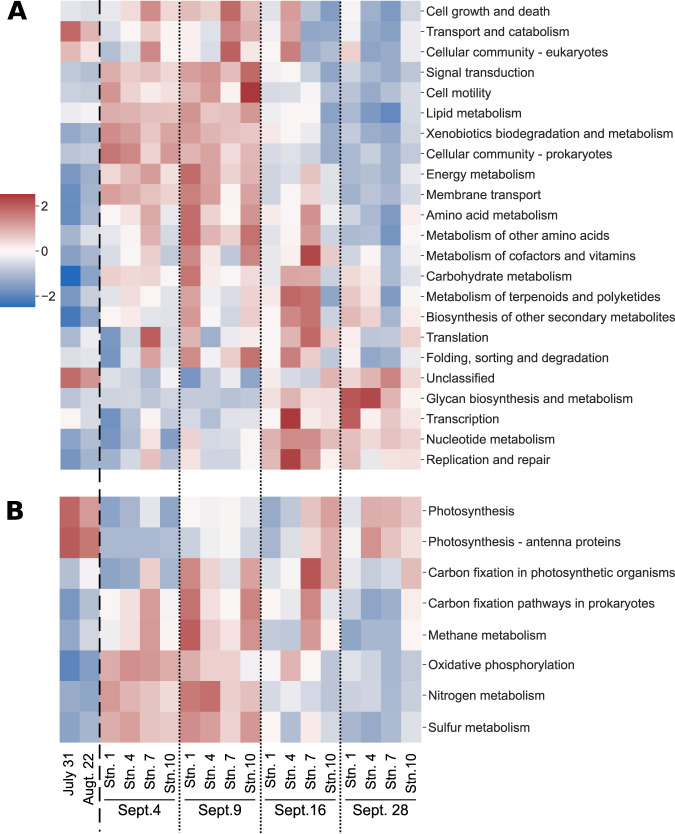
Heat map showing the *z*-score for genes in the complete metagenomes that were in the KEGG category of energy metabolism. The *z*-score is calculated using the proportion of KEGG pathways (**A**) and KEGG modules (**B**) identified in each metagenome to the total number of Prodigal predicted genes, darker red (3.5) there are above average reads and darker blue (−3.5) there are below average reads.

We evaluated the genomic potential of the microbial community following Harvey using KEGG classifications (Fig. 6). Genes associated with common photosynthetic processes (photosystems I and II, cytochrome b6f, and photosynthetic antenna proteins) decreased after the storm (Fig. 6B). There was a −1.46 fold-change in photosynthetic genes and −2.38 fold-change in antenna proteins on Sept. 4 compared to pre-Harvey. These genes never fully recovered with pre-Harvey fold-changes remaining at −0.32 for photosynthetic genes and −0.69 for antenna proteins on Sept. 28. Coupled with the decrease in photosynthetic genes were enrichments in virtually every other KEGG global pathway except for transport and catabolism, cellular community-eukaryotes, and transcription (Fig. 6A). While values were higher than average, as indicated by higher *z*-scores, the fold changes in relative abundance within each metagenome was less than 0.45 for all global pathways, indicating less than 45% increase in relative abundances of genes for all pathways. Amino acid metabolism and xenobiotic degradation and metabolism exhibited the clearest differentiation between pre- and post-Harvey (Supplementary Figs. 3 and 4). Analysis of the dissimilarity indices of KEGG genes agreed with the results from the taxonomy with the bioenv() results indicating the temperature, salinity, and pH were the most influential factors and that the functions grouped significantly by sampling date (analysis of similarity *R* > 0.7 and *p* < 0.05).

Among the KEGG modules associated with sulfur metabolism, genes involved in assimilatory sulfate reduction and cysteine biosynthesis were the most abundant and drove the increase in sulfur metabolism related genes seen in Sept. 4 and Sept. 9 (Fig. 6B). The highest relative abundance of genes involved in assimilatory sulfate reduction was seen on Sept. 4 while the highest relative abundance of genes involved in cysteine biosynthesis was recorded on Sept. 16. Dissimilatory sulfate reduction genes did not follow these trends, decreasing on Sept. 4 and Sept. 9 and returning to near pre-Harvey relative abundances by Sept. 16.

**Fig. 7:**
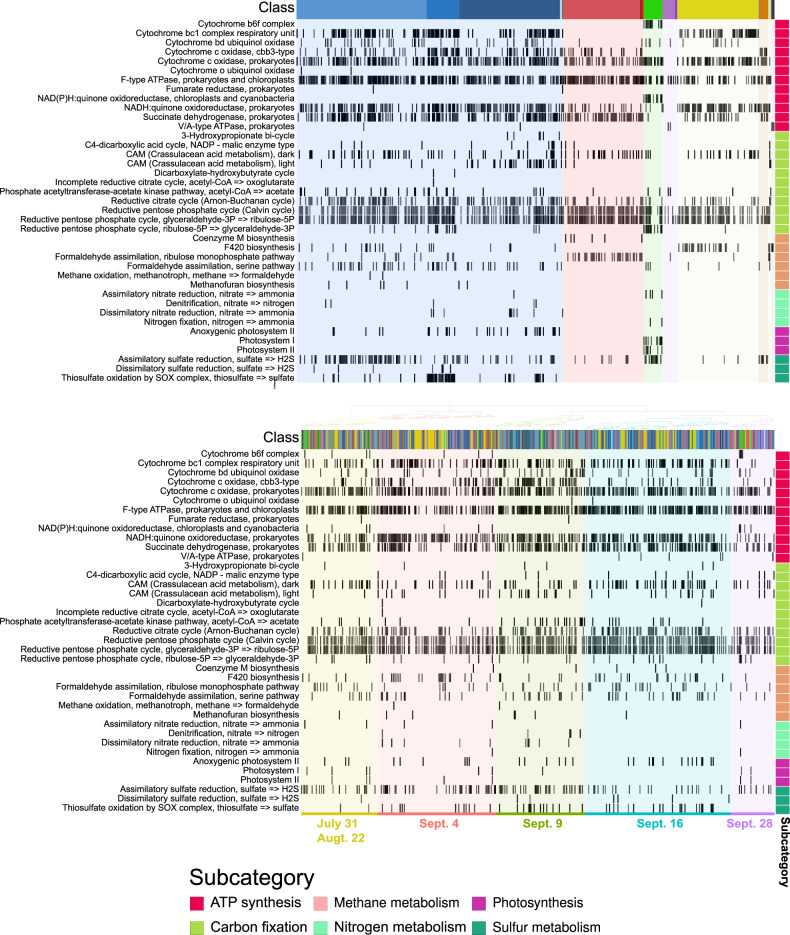
Heat map showing the percent completeness (opacity of the line) of KEGG pathways in the category Energy Metabolism (showing only modules with> 50% genes in each pathway). The MAGs were grouped according to taxonomic classification (**A**) and hierarchical clustering of the MAG percent community (**B**). Each column represents a MAG while each row represents a KEGG metabolism module subcategory.

To investigate major contributors to the metabolic changes observed in the complete gene pool we annotated and estimated the metabolic pathways within each MAG. Except for anoxygenic photosystem II, all photosynthesis KEGG modules were present in MAGs from the class Cyanophyaceae (Fig. 7A). Of the 18 Cyanophyaceae MAGs analyzed, 11 of them had at least one photosynthetic KEGG module present (with >50% completion). The majority of the Cyanophyaceae MAGs with photosynthetic KEGG modules cluster to either pre-Harvey or Sept. 28, with three MAGs clustering on Sept. 4 and Sept. 9 (Fig. 7B). Of these outliers, two had rRNA genes, one with 100% identity to *Cylindrospermopsis raciborskii* (accession number LC455653), a freshwater cyanobacteria, (MAG #864), the other with 100% identity to an uncultured Archaeosporales (accession number MH982482), a fungi found as a plant or Nostoc symbiont [46], (MAG #617) (Supplementary Table 4). The anoxygenic photosystem II KEGG module was found exclusively in the three Proteobacterial classes (Fig. 7A). Even though photosynthesis related genes were found in a limited number of MAGs, genes related to carbon fixation in photosynthetic organisms were found in every class except for Nitrososphaeria and Nitrospira (Fig. 7A). Additionally, the only two MAGs containing the nitrogen fixation KEGG module genes were both Cyanophyaceae MAGs.

Predicted genes associated with sulfur metabolims [assimilatory sulfate reduction, sulfur oxidation (SOX), or dissimilarity sulfate reduction] were found in 150 MAGs among eight different classes including all Proteobacteria, Flavobacteria, Actinobacteria, Opituate, Nitrospira, and Cyanophyaceae (Fig. 7A). Genes predicted to be associated with assimilatory sulfate reduction were found in 110 MAGs, while SOX complex genes were present in 49 MAGs, and dissimilatory sulfate reduction genes were present in 7 MAGs (Fig. 7). Genes related to dissimilatory sulfate reduction and the SOX complex were exclusive to Proteobacteria (Fig. 7A). Genes predicted to be involved in assimilatory sulfate reduction were prevalent throughout all sampling dates; however a majority of MAGs containing genes involved in the SOX complex (47 of 49 MAGs) and dissimilatory sulfate reduction (6 of 7 MAGs) clustered on Sept. 4, Sept. 9, and Sept. 16 (Fig. 7B).

**Fig. 8:**
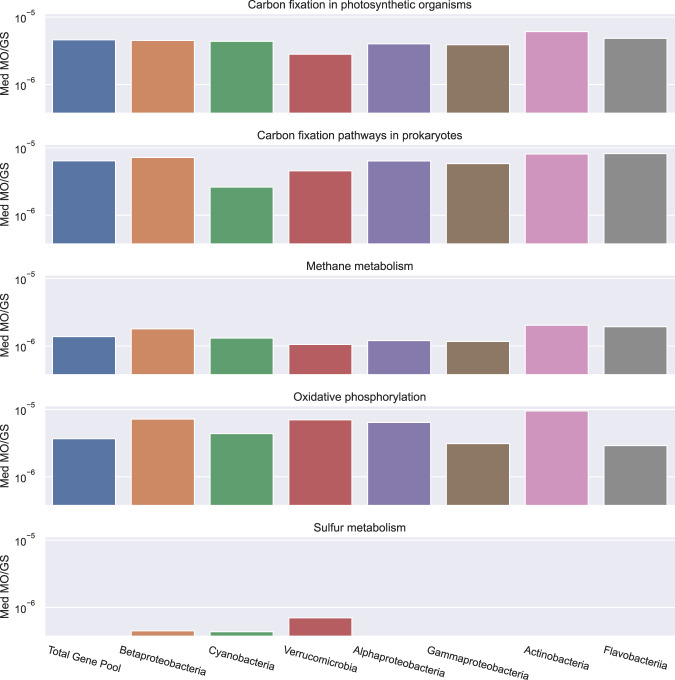
Median metabolic overlap of the KEGG energy metabolisms modules calculated for the total community and the dominant classes. KEGG energy metabolism modules with a median metabolic overlap of 0 in the total gene pool and the classes examined are not shown.

The redundancy in metabolism was assessed by the calculation of metabolic overlap for the total community and the dominant phyla (Fig. 8). The median metabolic overlap for the entire community is similar to calculations made in Hester et al. [45] for marine systems. Additionally, we found that photosynthesis, nitrogen metabolism, and xenobiotic degradation and metabolisms had median metabolic overlap scores of 0, indicating that these are rarer metabolisms among the species in the community. Sulfur metabolism had a score of 0 for the overall community but did show a higher than 0 median metabolic overlap in the phyla Cyanobacteria and Verrucomicrobia, and the class Betaproteobacteria (Fig. 8).

## Discussion

One of the major threats global climate change poses to estuarine systems is the potential for increased frequency and severity of storms and flooding [6]. Additional threats are imposed on industrialized watersheds, which receive stormwater runoff containing increased concentrations of nutrients and chemicals from urban and agricultural sources. The impact of such events on phytoplankton communities has been shown to be dependent on the magnitude of river flows, with higher magnitudes favoring species with high rates of reproduction and lower flows benefiting smaller species, particularly cyanobacteria [25, 47–49]. While several studies focusing on phytoplankton have been conducted, less is known about bacterial responses. Studies that examine the overall bacterial community have typically been limited to cell counts, fecal indicators, or specific marker genes [50, 47, 51]. Here, we took advantage of the opportunity provided by the passage of Harvey to identify the shifts within the microbial population affected by an extreme rain event in a highly industrialized coastal estuary, Galveston Bay, using metagenomics.

### Metabolically diverse microorganisms showed resiliency while primary producers were negatively impacted and some rare taxa thrived

Harvey drastically changed the physicochemical conditions of Galveston Bay, decreasing the salinity (30.43 psu to 0.02 psu), pH (8.4 to 6.32), and temperature (31.37 °C to 24.22 °C) while increasing suspended sediment and turbidity (Supplementary Table 1). The pulse of storm water runoff has been shown to have altered the microbial populations causing large shifts from a mainly estuarine to that of a freshwater dominated community [10]. Additional studies surrounding Harvey have identified that the large abiotic shifts observed along with inputs of terrestrial dissolved organic carbon led to specific changes in aromatic carbon degradation and fecal indicator species [28, 51, 52]. Similarly, our data showed how the taxonomic composition of the whole gene pool and MAGs tended to be more similar according to sampling date rather than location within Galveston Bay (Figs. 2 and 3) supporting the idea that the changes post-Harvey had more effect on community structure than the differences between the locations within Galveston Bay. Unlike previous Harvey findings, the whole gene pool characterized in this study shows relatively small changes in the dominant members of the community such as Alphaproteobacteria, Gammaproteobacteria, Actinobacteria, and Flavobacteria following Harvey (Figs. 2 and 4A). These four classes are made up of diverse species and are found in terrestrial, freshwater, and marine ecosystems. The distribution of the metagenomic profiles related to these bacteria, as well as the metabolic potential of the MAGs, demonstrates how these more metabolically diverse members were able to remain near the same relative abundance following Harvey (Figs. 5 and 6). Additionally, the enrichment of energy metabolisms, all amino acid metabolisms, and xenobiotic degradation related genes show that the conditions following Harvey were supporting microorganisms with more diverse metabolic capabilities (Fig. 6, Supplementary Figs. 3 and 4).

The differences in our study and previous studies, particularly Steichen et al. which sequenced amplicons of separate samples taken on the same cruises, are likely due to the inherent differences between amplicon sequencing and metagenomics. For instance, amplicon sequencing is accurate at identification down to the genus level but has been shown to be biased to certain taxonomic groups and the different databases used do not have a consistent system of classification [53–56]. Metagenomics is capable of making identifications at the species level and tends to make more at every taxonomic level, however, classification is highly dependent on the choices of bioinformatic programs, the options specified in the programs, and the databases used all of which bring their own biases [53–56].

In agreement with Steichen et al.’s [10] findings, the relative abundance of more metabolically limited, or specialized, members of the community such as Cyanobacteria, Betaproteobacteria, and Verrucomicrobia were altered by the influx of freshwater, nutrients, and sediment brought by Harvey. Cyanobacteria were likely flushed out of the bay prior to the first sampling event, leading to the depression in their relative abundance and in the relative abundance of genes involved in photosynthesis and nitrogen fixation observed here (Figs. 2 and 4). During our study, three cyanobacterial MAGs clustering to Sept. 4 were not present before Harvey or after Sept. 9. These are the only MAGs identified with oxygenic photosynthetic genes or  nitrogen fixation during the first two weeks following Harvey, emphasizing their importance in the community. The shift from one of the dominant members of the community (10–26%) to accounting for less than 3% of the community shows how extreme weather events have the potential to drastically impact ecologically important bacteria that play an important role within the community, in this case primary producers, and thus impact ecosystem services (Figs. 2 and 4).

Both Betaproteobacteria and Verrucomicrobia were enriched following Harvey (Figs. 2 and 5). Betaproteobacteria MAGs were enriched in the SOX complex as well as anoxygenic photosynthesis (Fig. 7). The presence of these pathways and classifications, based on Kraken2 and 16S rRNA genes, would indicate that these are purple non-sulfur bacteria that are typically associated with anoxygenic, low light systems such as sediments, soil, or wastewater [57]. The analysis of active Verrucomicrobial single cell genomes have shown that they could be important polysaccharide degraders [58]. The enrichment of Betaproteobacteria and Verrucomicrobia is consistent with the enrichment of terrestrial organic matter derived from leaves and grasses in Galveston Bay following Harvey [28]. These microbes were likely brought in by the resuspension of sediment from the storm and were able to thrive in the altered conditions of Galveston Bay following the storm. Over time the advantage of additional substrates and reduced competition were lessened, and their relative abundances decreased. While rare, these microbes were present prior to Harvey and show that pulse disturbances can allow microbial groups that normally coalesce at lower relative abundances to flourish.

### Redundant and diverse metabolic potential make for a resilient microbial community

Results from mSourceTracker showed that the community shifted to that of freshwater and soil influenced (Fig. 5). Taken with the metabolic analysis of the whole gene pool and the MAGs, these results indicate that a diverse array of bacteria were introduced into Galveston Bay. The resident community appeared to have been flushed out immediately after Harvey [7]. The continued discharge of flood waters, slowing of transit times of particles (particulates, detrital matter), and return of tidal currents would have allowed for a unique coalescence of microbial communities including those normally in Galveston Bay as well as transient groups introduced by waste waters, urban runoff, sediment, and soil [7, 59]. Given the altered microbial community structure and the depression in the relative abundance of photosynthetic genes, the changes in physiochemical properties (e.g., suspend sediments, temperature, salinity) were likely exerting pressure on the microbial community, selecting for organisms with more diverse metabolic potential, mainly heterotrophs. The higher than pre-Harvey rates of virtually every KEGG metabolism reflects a community adapted for a wide range of conditions and energy inputs (Fig. 6).

Despite the change in community structure, pathways present in MAGs were similar across the sampling date clusters including photosynthetic pathways (Fig. 4A and 7B). The presence of similar metabolic genes throughout the sampling period implies that estuaries like Galveston Bay harbor individuals capable of performing the same ecosystem services while at the same time adapted to a range of abiotic conditions. This point is further illustrated by the gradients of abiotic conditions and microbial community composition in later samplings, when the environmental conditions were returning to normal (Supplementary Table 1 and Fig. 5). The redundancy between dominant marine specialists and freshwater/terrestrial specialists was most evident in the case of Cyanobacteria, Betaproteobacteria, and Verrucomicrobia. Cyanobacteria MAGs were enriched in sulfate reduction genes and were the only prevalent pre-Harvey members with a metabolic overlap above 0 for the MEGG module sulfur metabolism (Figs. 7 and 8). Similarly, the Verrucomicrobia and Betaproteobacteria MAGs contained high levels of sulfate reduction and were the only other groups with metabolic overlap scores above 0 for sulfur metabolisms (Figs. 7 and 8). Additionally, the photosynthetic genes from the typically marine Cyanobacteria MAGs lost in the gene pool were present, albeit at lower relative abundances, in freshwater species. Despite the unprecedented amount of rainfall that Harvey deposited, the dominant members of the bacterial community were still present in the water column, although at varying relative abundances. Our results suggest that rarer taxa such as Verrucomicrobia and Betaproteobacteria were able to thrive by taking advantage of the shifts in physiochemical characteristics such as the large input of organic matter. Overall, our results highlight that the microbial communities of the Galveston Bay ecosystem, which are adapted to gradients of fresh to marine environmental estuarine conditions, are resilient to disturbances like Harvey due to their metabolic redundancy.

## Conclusions

Harvey was a record-breaking pulse disturbance. While traditional approaches documented changes in abundance and diversity [10, 28, 52], the use of metagenomics in this study allowed us to place the microbial community composition within the context of the total genetic pool. The results mirror previous findings in that typically photosynthetic marine Cyanobacteria were flushed out of Galveston Bay and replaced with terrestrially derived Betaproteobacteria and Verrucomicrobia. Where our study differed was the observed lack of a change to taxa that tend to be metabolically diverse, such as Alphaproteobacteria, Gammaproteobacteria, Actionbacteria, and Flavobacteria. By reconstructing genomes within the total gene pool, we were able to identify dominate members of the community that also exhibited resilience to disturbances as well as diverse metabolisms present before, directly following, and a month after Harvey. This study highlights the resilience of microbial communities, however, with the prediction of increased severity and frequency of storms it remains to be seen if these systems can withstand prolonged or continuous disturbances.

## Supplementary information

Supplementary Material

## Data Availability

All metagenomes are publicly available in the MG-RAST metagenomics analysis server and NCBI SRA Archive (accession numbers listed in Supplementary Table 3).
