# Supplementary material for: Functionally diverse microbial communities show resilience in response to a record-breaking rain event
Source: ISME Commun. 2022 Sep 2;2:81. doi: 10.1038/s43705-022-00162-z (PMC9723638; doi:10.1038/s43705-022-00162-z)
Supplement: Supplementary file 1 — Supplementary Material [file 43705_2022_162_MOESM1_ESM.docx]

**Supplementary Material**

**Functionally diverse microbial communities show resilience in response to a record-rain event**

Jordan R. Walker^1^, Alaina C. Woods^1^, Mary K. Pierce^1^, Jamie L. Steichen^1^, Antonietta Quigg^1^, Karl Kaiser^2,3^, Jessica M. Labonté^1^

**Supplementary Material and Methods**

***Whole Community Bioinformatics Pipeline***

Quality control of the metagenomic reads was performed with BBMap as follows. BBduk was run to remove sequencing artifacts using the files containing common sequencing artifacts provided by BBMap. A second BBduk command was used to trim reads that contained adapter sequence and had low quality scores (Phred score Q<10). BBmerge was used to merge the forward and reverse paired-end reads using default settings (Bushnell et al., 2017a). BBmask was used to soft-mask regions of low complexity (Shannon entropy < 0.7). MEGAHIT was utilized to perform de novo assembly by grouping the GF and 0.22 fractions by specific sampling (Li et al., 2015). Gene prediction was performed with Prodigal (version 2.6.3) (Hyatt et al., 2010). Genes and proteins predicted by Prodigal were annotated by blastp alignment using DIAMOND (version 0.9.26), with the “sensitive” setting against the NCBI non-redundant protein database (created August 23, 2019). DIAMOND outputs were passed to MEGAN6, Ultimate Edition, for community analysis (Huson et al, 2016, 2011). Counts of genes were extracted from MEGAN6 and statistical analysis and visualization was performed in R.

The source of microbes was determined using mSourceTracker (McGhee et al., 2020) with sources including coastal marine, human gut, freshwater, and soil metagenomes. Each set of genes predicted for each metagenome from this study was used as an environmental sink. Taxonomic assignments of contigs in the source and sink metagenomes were made using Kraken2 (version 2.0.9) (Wood et al., 2019). The results were filtered to only retain reads classification at genus or lower level, and all other reads were designated “Unassigned”. The final mSourceTracker results were generated using 10 chains and 200 draws, which, using the diagnostic tool, allowed for less than 1% variability.

***MAG binning and analysis pipeline***

All merged and unmerged read files from all samples, refined using the BBtools package, were co-assembled using MEGAHIT (version 1.2.8) with a minimum contig length of 2000 bp. The merged and unmerged reads of each sampling date and location were then mapped back to the co-assembly using Burrows-Wheeler Aligner (BWA) (version 0.7.17) (Li and Durbin, 2009). Outputs of BWA were converted to BAM files and unmapped reads were filtered out using SAMtools (version 1.9) (Li *et al.*, 2009). The depth of coverage for each sequence was calculated using the jgi_summarize_bam_contig_depths script from the Metabat2 package (version 2.14) (Kang *et al.*, 2019). Binning of the contigs was completed using Metabat2 with minimum contig length set to 2 500 bp. Completion and contamination of each bin was determined using the lineage workflow in CheckM (version 1.1.3) (Parks *et al.*, 2015). The profile function in CheckM was used to estimate the percent community each bin represented in each sample. The CheckM profile function estimates the percent community based on the percentage of reads mapping to a bin, adjusted for the bin size and the number of reads mapping to assembled contigs. Taxonomy of the bins was assigned using Kraken2 (version 2.09) (Wood *et al.*, 2019), with the standard Kraken2 database used to generate the standard output along with the MetaPhlAn (MPA) formatted report. Taxonomy was determined by assigning a phylum, class, and order to each bin based on the highest number of fragments assigned at each level in each bin. Additionally, the ssu_finder function in CheckM was used to determine if bins contained any ribosomal genes, which were analyzed using BLASTn with default settings. Genes were predicted and annotated with GHOSTKOALA (Kanehisa *et al.*, 2016) which was then uploaded to Anvi’o (version 7) (Eren *et al.*, 2021). Anvi’o’s estimate metabolism function was used to determine the completion of KEGG modules. Bins were assigned quality based on the following standards: ≥90% complete and <5% contamination were of high quality, ≥50% complete and <10% contamination were of medium quality (not including high quality), and all other bins were low quality. Bins were then filtered to remove those of low quality and the remaining were considered good quality bins (Supplementary Figure 1). The remaining bins were clustered based on the percent community output from CheckM using Anvi’o’s matrix-to-newick function which uses Euclidean distance with ward linkages. Clusters were assigned sampling dates based on the highest cumulative percent community of MAGs within clades of the dendrogram generated by the previous step. The taxonomy and KEGG module completeness of each bin were exported from Anvi’o to tables for manual manipulation in R. The anvi-interactive manual mode or R was used for visualization of each dataset.

***Metabolic Overlap Calculation***

The Bio.kegg package was used to retrieve database entries and then link these entries to the KO numbers output by anvio-estimate-metabolism function. The result was a collection of reactants utilized within each KEGG metabolism contained in each MAG. Matches of reactants within each KEGG metabolism between each doublet of MAGs were counted and placed into a matrix. The matrices for each KEGG metabolism were then summed according to KEGG pathways or the overall community. Median numbers were calculated and normalized to median genome size for the overall community as well as the different taxonomic groups.

**References**

1. Bushnell B, Rood J, Singer E. BBMerge – Accurate paired shotgun read merging via overlap. 2017; 1–15.

2. Li D, Liu CM, Luo R, Sadakane K, Lam TW. MEGAHIT: An ultra-fast single-node solution for large and complex metagenomics assembly via succinct de Bruijn graph. *Bioinformatics* 2015; **31**: 1674–1676.

3. Hyatt D, Chen G-L, LoCascio PF, Land ML, Larimer FW, Hauser LJ. Prodigal: prokaryotic gene recognition and translation initiation site identification. *BMC Bioinformatics* 2010; **11**: 119.

4. Buchfink B, Xie C, Huson DH. Fast and sensitive protein alignment using DIAMOND. *Nat Methods* 2014; **12**: 59–60.

5. Huson DH, Beier S, Flade I, Górska A, El-Hadidi M, Mitra S, et al. MEGAN community edition-interactive exploration and analysis of large-scale microbiome sequencing data. *PLoS Comput Biol* 2016; **12**: e1004957.

6. Wood DE, Lu J, Langmead B. Improved metagenomic analysis with Kraken 2. *Genome Biol* 2019; **20**: 257.

7. Li H, Durbin R. Fast and accurate short read alignment with Burrows-Wheeler transform. *Bioinformatics* 2009; **25**: 1754–1760.

8. Li H, Handsaker B, Wysoker A, Fennell T, Ruan J, Homer N, et al. The sequence alignment/map format and SAMtools. *bioinformatics* 2009; **25**: 2078–2079.

9. Kang D, Kirton ES, Thomas A, Egan RS, Wang Z, Kang DD, et al. MetaBAT 2 : an adaptive binning algorithm for robust and efficient genome reconstruction from metagenome assemblies MetaBAT 2 : an adaptive binning algorithm for robust and efficient genome reconstruction from metagenome assemblies. 2019; 0–10.

10. Parks DH, Imelfort M, Skennerton CT, Hugenholtz P, Tyson GW. CheckM: assessing the quality of microbial genomes recovered from isolates, single cells, and metagenomes. *Genome Res* 2015; **25**: 1043–1055.

11. Kanehisa M, Sato Y, Morishima K. BlastKOALA and GhostKOALA: KEGG tools for functional characterization of genome and metagenome sequences. *J Mol Biol* 2016; **428**: 726–731.

12. Eren AM, Kiefl E, Shaiber A, Veseli I, Miller SE, Schechter MS, et al. Community-led, integrated, reproducible multi-omics with anvi’o. *Nat Microbiol* 2021; **6**: 3–6.

**Supplementary Figures**


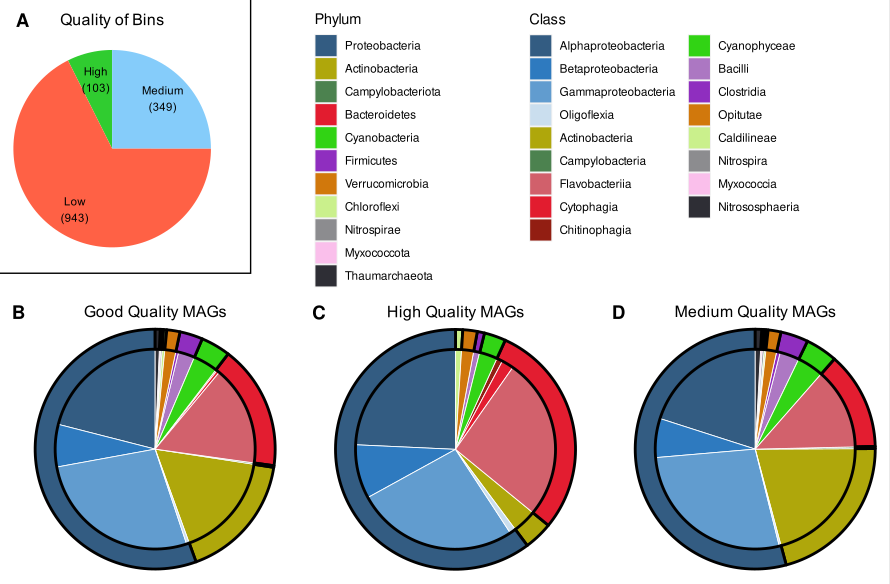


**Supplementary Figure 1:** Number of MAGs classified as high, medium, and low quality (A). Breakdown of the taxonomic classification of all good quality MAGs, those classified as either high or medium quality (A), only high quality MAGs (B), and only medium quality MAGs (C).


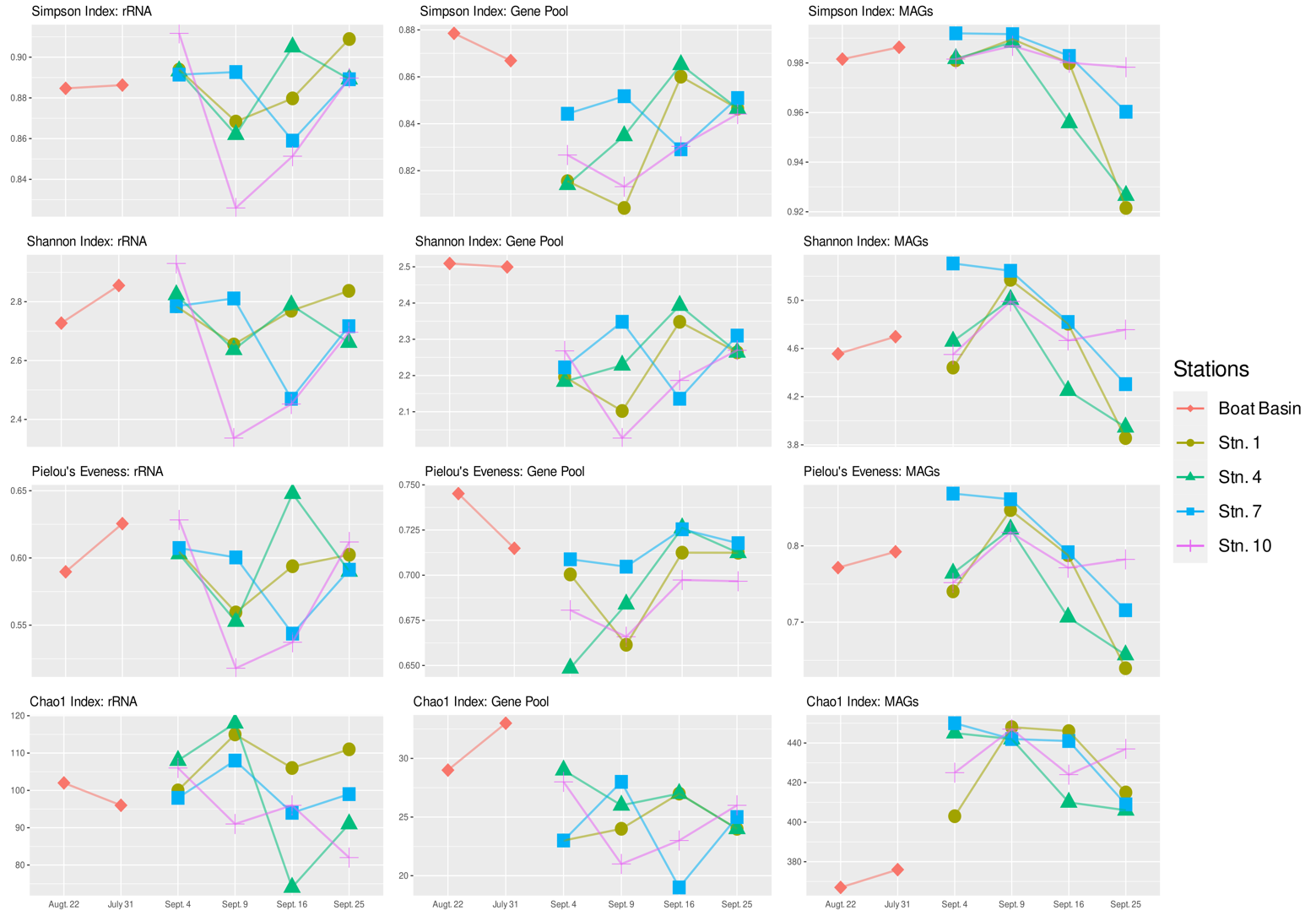


**Supplementary Figure 2:** Estimates of diversity, richness, and evenness for the 16S rRNA genes, the total gene pool, and the MAGs found in the metagenomes. Each calculation is separated by sampling date.


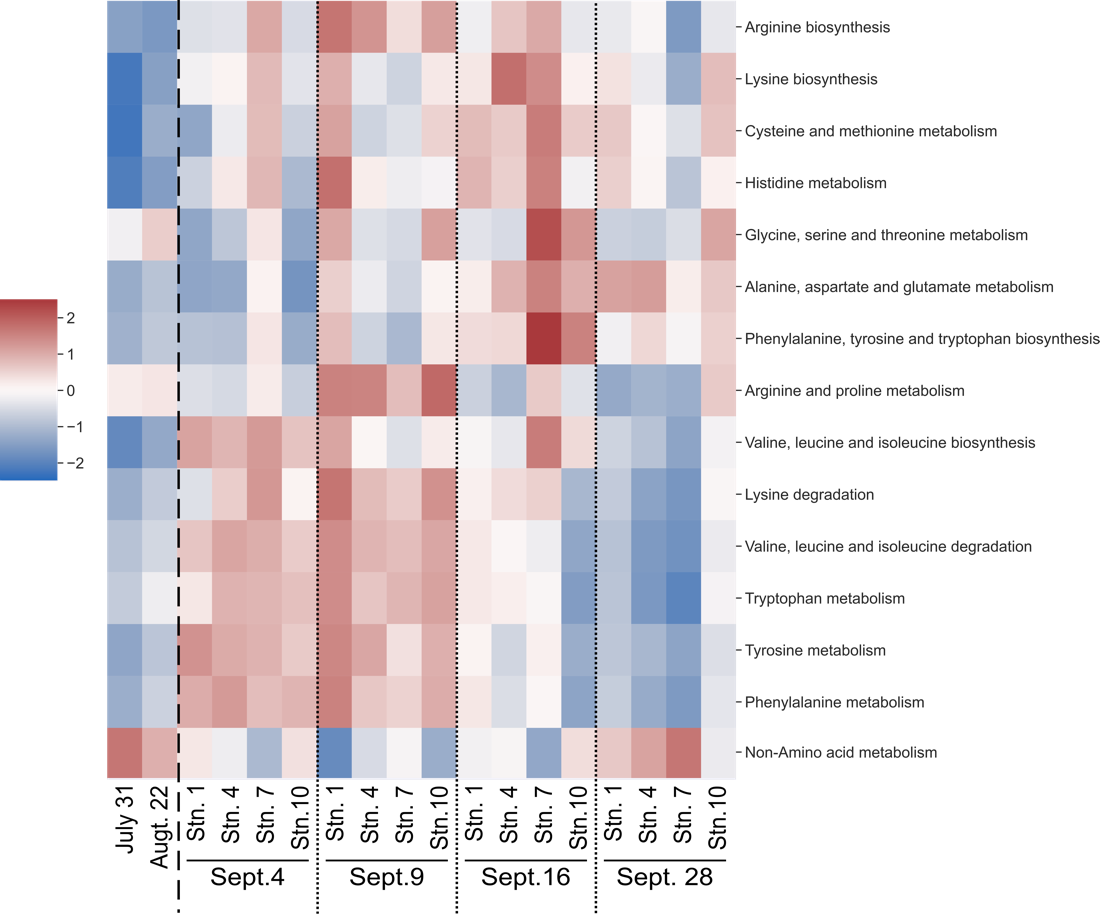


**Supplementary Figure 3.** Heatmap of amino acid metabolisms found in the total gene pool. Gene counts were normalized to the total number of predicted genes in each metagenome then z-score transformed across metagenomes.


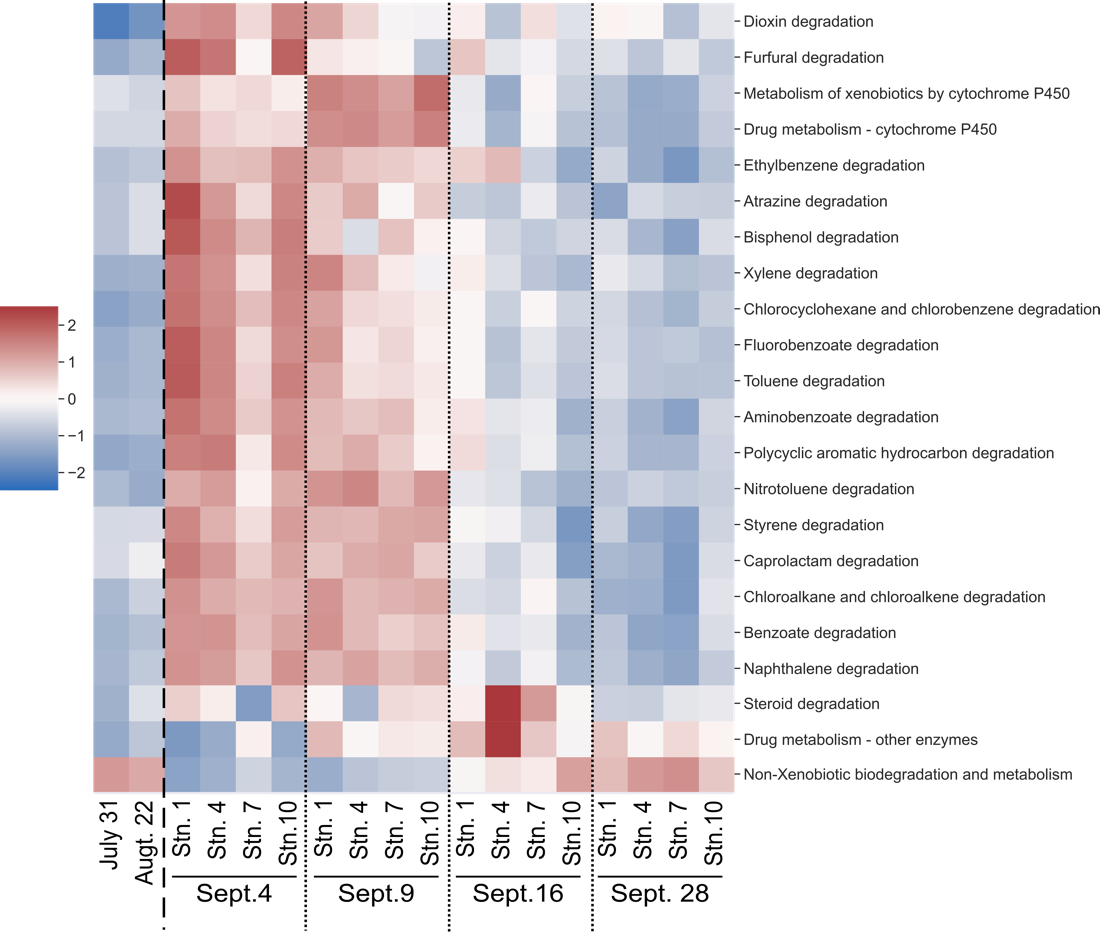


**Supplementary Figure 4.** Heatmap of xenobiotics degradation and metabolisms found in the total gene pool. Gene counts were normalized to the total number of predicted genes in each metagenome then z-score transformed across metagenomes.

**Supplementary Table 1:** Physiochemical properties of Galveston Bay throughout the sampling period, adapted from Steichen et al. (2019).

| **Field Observations** | | | | | | | **Hydrolab** | | | | | | | | **Refrac** | **Secchi** |
| --- | --- | --- | --- | --- | --- | --- | --- | --- | --- | --- | --- | --- | --- | --- | --- | --- |
| Date | Time | Trip Type | Station | Lat | Long | Wave Ht (ft) | Depth (m) | Temp (°C) | Salinity (psu) | LDO (mg/L) | LDO % | pH | SpC (mS/cm) | TSS  (g mL-1) | psu | m |
| 7/19/17 | 9:47 | *Pre-Storm* | TAMUG Boat Basin | 29.32 | -94.85 | 0 | 0 | 29.97 | 22.29 | 6.34 | 92.5 | 8.11 | 33.36 |  |  |  |
| 7/29/16 | 9:33 | *Pre-Storm* | TAMUG Boat Basin | 29.32 | -94.85 | 0 | 0 | 30.71 | 22.78 | 6.79 | 104.5 | 8.37 | 36.1 |  |  |  |
| 8/1/16 | 9:45 | *Pre-Storm* | TAMUG Boat Basin | 29.32 | -94.85 | 0 | 0 | 30.71 |  |  |  |  |  |  |  |  |
| 8/21/17 | 12:15 | *Pre-Storm* | TAMUG Boat Basin | 29.32 | -94.85 | s | 0 | 31.37 | 30.43 | 5.67 | 92.9 | 8.1 | 44.71 |  |  |  |
| 8/22/17 | 12:15 | *Pre-Storm* | TAMUG Boat Basin | 29.32 | -94.85 | 0 | 0 | 29.16 | 29.00 |  |  | 8.40 |  |  |  |  |
| 8/30/17 | 11:30 | *Post-Storm* | TAMUG Boat Basin | 29.32 | -94.85 | 1 | 0 | 24.22 | 7.32 | 7.56 | 96.6 | 7.87 | 13.10 |  |  |  |
| 8/31/17 | 10:00 | *Post-Storm* | TAMUG Boat Basin | 29.32 | -94.85 | 0 | 0 | 25.13 | 2.34 | 7.25 | 91.4 | 7.91 | 4.29 |  |  |  |
| 9/1/17 | 9:38 | *Post-Storm* | TAMUG Boat Basin | 29.32 | -94.85 | 0 | 0 | 26.11 | 3.74 | 6.64 | 85.5 | 7.80 | 6.10 |  |  |  |
| 9/2/17 | 9:35 | *Post-Storm* | TAMUG Boat Basin | 29.32 | -94.85 | 0 | 0 | 26.90 | 6.20 | 5.14 | 68.6 | 7.65 | 10.98 |  |  |  |
| 9/3/17 | 9:19 | *Post-Storm* | TAMUG Boat Basin | 29.32 | -94.85 | 0 | 0 | 27.59 | 6.34 | 5.13 | 69.2 | 7.71 | 11.17 |  |  |  |
| 8/22/17 | 10:09 | *Pre-Storm* | GB4 | 29.60866 | -94.92953 | 0 | 0 | 31.18 | 12.83 | 7.13 | 106.9 | 8.3 | 21.390 |  | 15.0 | 0.59 |
| 9/4/17 | 9:25 | R/V Trident | Stn1 | 29.6725 | -94.976944 | 0 | 0 | - | - | - | - | - | - |  | 0.0 | 0.25 |
|  |  |  | Stn1 |  |  |  | 1 | 27.23 | 0.02 | 7.63 | 96.2 | 6.32 | 0.054 | 20.00 |  |  |
| 9/4/17 | 11:15 | R/V Trident | Stn4 | 29.535556 | -94.897222 | 0.5 | 0 | 27.66 | 0.84 | 6.28 | 80.9 | 7.42 | 1.595 | 82.00 | 0.5 | 0.25 |
| 9/4/17 | 12:47 | R/V Trident | Stn7 | 29.4125 | -94.825833 | 0.5 | 0 | 27.79 | 0.81 | 6.12 | 80.1 | 7.37 | 1.546 | 88.00 | 1.0 | 0.25 |
| 9/4/17 | 14:10 | R/V Trident | Stn10 | 29.333611 | -94.688611 | 0.5 | 0 | 28.92 | 6.57 | 6.56 | 90.9 | 7.83 | 11.530 | 30.00 | 7.0 | 0.50 |
| 9/9/17 | 9:09 | R/V Trident | Stn1 | 29.670556 | -94.978889 | 0 | 0 | 25.93 | 4.35 | 5.24 | 67.4 | 7.42 | 7.787 | 96.00 | 4 | 0.25 |
| 9/9/17 | 10:45 | R/V Trident | Stn4 | 29.534167 | -94.899722 | 1 | 0 | 25.78 | 4.09 | 6.88 | 88.9 | 7.71 | 7.315 | 112.00 | 4 | 0.25 |
| 9/9/17 | 12:15 | R/V Trident | Stn7 | 29.411944 | -94.826667 | 1 | 0 | 25 | 4.52 | 7.52 | 97.7 | 7.88 | 8.275 | 70.00 | 5 | 0.5 |
| 9/9/17 | 13:51 | R/V Trident | Stn10 | 29.335 | -94.690833 | 2 | 0 | 26.73 | 10.38 | 7.55 | 103 | 8.03 | 17.46 | 20.00 | 10 | 0.75 |
| 9/16/17 | 9:35 | R/V Trident | Stn1 | 29.671 | -94.9787 | 0 | 0 | 26.97 | 5.4 | 6.4 | 85.1 | 7.73 | 9.557 | 59.33 | 5 | 0.4 |
| 9/16/17 | 10:46 | R/V Trident | Stn4 | 29.5373 | -94.9016 | 0 | 0 | 27.34 | 4.16 | 8.79 | 116.5 | 8.36 | 8.651 | 24.67 | 6 | 0.5 |
| 9/16/17 | 11:50 | R/V Trident | Stn7 | 29.4118 | -94.8216 | 0 | 0 | 28.48 | 12.18 | 8.78 | 124.3 | 8.33 | 19.79 | 23.33 | 11 | 0.75 |
| 9/16/17 | 12:51 | R/V Trident | Stn10 | 29.3344 | -94.6959 | 1 | 0 | 28.32 | 19.38 | 7.76 | 114.9 | 8.22 | 31.54 | 26.00 | 20 | 2.5 |
| 9/28/17 | 9:25 | R/V Trident | Stn1 | 29.668056 | -94.978056 | 0 | 0 | 28.92 | 7.84 | 5.94 | 82.8 | 7.92 | 13.62 | 18.67 | 8 | 0.5 |
| 9/28/17 | 10:27 | R/V Trident | Stn4 | 29.535 | -94.900278 | 0 | 0 | 29.03 | 8.31 | 7.75 | 104 | 8.26 | 14.35 | 10.67 | 8 | 1 |
| 9/28/17 | 11:23 | R/V Trident | Stn7 | 29.414167 | -94.827222 | 0.5 | 0 | 29.19 | 13.6 | 7.14 | 103.4 | 8.14 | 22.6 | 6.67 | 14 | 0.9 |
| 9/28/17 | 12:22 | R/V Trident | Stn10 | 29.332778 | -94.691111 | 0.5 | 0 | 29.55 | 20.08 | 6.99 | 105.4 | 8.13 | 32.09 | 4.00 | 21 | 3 |

**Supplementary Table 2:** Filtrations details for each sampling including the total amount of volume filtered, the filter types, and the fraction of the filter used for DNA extraction (Samp1=Sept. 4; Samp2=Sept. 9; Samp3=Sept. 16; Samp5=Sept. 28; Pre-Harvey1=July31; Pre-Harvey2=Augt.22).

| SampID | Sampling Date | Coordinates | Volume Filtered (L) | Filters Used | Fraction of Filters (DNA extraction) |
| --- | --- | --- | --- | --- | --- |
| Samp1_Stn1 | 9/4/17 | - 94.976944, 29.6725 | 4 | GF/D (2.7 µm) then 0.22 µm PVDF | 1.00 |
| Samp1_Stn4 | 9/4/17 | - 94.897222, 29.535556 | 4 | GF/D (2.7 µm) then 0.22 µm PVDF | 1.00 |
| Samp1_Stn7 | 9/4/17 | - 94.825833, 29.4125 | 4 | GF/D (2.7 µm) then 0.22 µm PVDF | 1.00 |
| Samp1_Stn10 | 9/4/17 | - 94.688611, 29.333611 | 4 | GF/D (2.7 µm) then 0.22 µm PVDF | 1.00 |
| Samp2_Stn1 | 9/9/17 | - 94.976944, 29.6725 | 10 | GF/D (2.7 µm) then 0.22 µm PVDF | 0.50 |
| Samp2_Stn4 | 9/9/17 | - 94.897222, 29.535556 | 10 | GF/D (2.7 µm) then 0.45 µm PVDF | 0.50 |
| Samp2_Stn7 | 9/9/17 | - 94.825833, 29.4125 | 10 | GF/D (2.7 µm) then 0.45 µm PVDF | 0.50 |
| Samp2_Stn10 | 9/9/17 | - 94.688611, 29.333611 | 10 | GF/D (2.7 µm) then 0.45 µm PVDF | 0.50 |
| Samp3_Stn1 | 9/16/17 | - 94.976944, 29.6725 | 17 | GF/D (2.7 µm) then 0.45 µm PVDF | 0.25 |
| Samp3_Stn4 | 9/16/17 | - 94.897222, 29.535556 | 17.5 | GF/F (0.7 µm) then 0.22 µm PVDF | 0.25 |
| Samp3_Stn7 | 9/16/17 | - 94.825833, 29.4125 | 20 | GF/F (0.7 µm) then 0.22 µm PVDF | 0.25 |
| Samp3_Stn10 | 9/16/17 | - 94.688611, 29.333611 | 10 | GF/F (0.7 µm) then 0.22 µm PVDF | 0.50 |
| Samp5_Stn1 | 9/28/17 | - 94.976944, 29.6725 | 20 | GF/F (0.7 µm) then 0.22 µm PVDF | 0.25 |
| Samp5_Stn4 | 9/28/17 | - 94.897222, 29.535556 | 10 | GF/F (0.7 µm) then 0.22 µm PVDF | 0.50 |
| Samp5_Stn7 | 9/28/17 | - 94.825833, 29.4125 | 15.75 | GF/F (0.7 µm) then 0.22 µm PVDF | 0.33 |
| Samp5_Stn10 | 9/28/17 | - 94.688611, 29.333611 | 19.5 | GF/F (0.7 µm) then 0.22 µm PVDF | 0.25 |
| Pre-Harvey1 | 7/31/17 | - 94.85, 29.32 |  | GF/D (2.7 µm) then 0.22 µm PVDF |  |
| Pre-Harvey2 | 8/22/17 | - 94.85, 29.32 | 21 | GF/D (2.7 µm) then 0.22 µm PVDF | 0.25 |

**Supplementary Table 3:** Quality control, assembly, gene prediction statistics, and database accession numbers for each metagenome.

| **Sample ID** | **Filter Size** | **Total # Contigs** | **Total bp Assembled** | **Min Contig Length** | **Max Contig Length** | **Average Contig Length** | **N50** | **# Proteins** | **# reads** | **# reads post-QC** | **% merged** | **% masked (merged)** | **NCBI accession** | **MG-Rast ID** | **MG-Rast Metagenome ID** |
| --- | --- | --- | --- | --- | --- | --- | --- | --- | --- | --- | --- | --- | --- | --- | --- |
| **Samp1_Stn1** | 0.22 | 584,688 | 380,807,570 | 200 | 181,496 | 651 | 668 | 828,435 | 32,165,848 | 32,035,118 | 19.31 | 0.02 | SRR13054106 | mgl817989 | mgm4882934.3 |
| **Samp1_Stn4** | 0.22 | 540,483 | 366,600,185 | 200 | 132,707 | 678 | 714 | 783,902 | 34,420,532 | 34,274,700 | 14.65 | 0.02 | SRR13054094 | mgl817995 | mgm4882972.3 |
| **Samp1_Stn7** | 0.22 | 538,660 | 360,809,803 | 200 | 89,548 | 670 | 704 | 790,577 | 31,429,186 | 31,317,318 | 17.26 | 0.04 | SRR13054083 | mgl817998 | mgm4882949.3 |
| **Samp1_Stn10** | 0.22 | 547,564 | 371,660,270 | 200 | 166,982 | 679 | 709 | 792,777 | 33,958,200 | 33,835,650 | 17.11 | 0.04 | SRR13054105 | mgl817992 | mgm4882960.3 |
| **Samp2_Stn1** | 0.45 | 574,772 | 418,240,335 | 200 | 81,556 | 728 | 814 | 864,246 | 36,157,034 | 36,001,282 | 28.26 | 0.03 | SRR13054100 | mgl818031 | mgm4882965.3 |
| **Samp2_Stn4** | 0.45 | 577,526 | 417,442,972 | 200 | 543,787 | 723 | 794 | 857,422 | 35,438,314 | 35,333,472 | 18.92 | 0.06 | SRR13054098 | mgl818037 | mgm4882958.3 |
| **Samp2_Stn7** | 0.45 | 520,220 | 357,986,102 | 200 | 80,516 | 688 | 731 | 763,602 | 29,597,168 | 29,525,854 | 22.2 | 0.05 | SRR13054097 | mgl818040 | mgm4882942.3 |
| **Samp2_Stn10** | 0.45 | 403,307 | 304,838,186 | 200 | 70,494 | 756 | 872 | 606,950 | 23,472,810 | 23,415,436 | 33.08 | 0.07 | SRR13054099 | mgl818034 | mgm4882930.3 |
| **Samp3_Stn1** | 0.22 | 444,073 | 322,839,415 | 200 | 99,903 | 727 | 809 | 684,673 | 27,420,504 | 27,345,366 | 23 | 0.03 | SRR13054072 | mgl818001 | mgm4882928.3 |
| **Samp3_Stn4** | 0.22 | 490,412 | 381,694,878 | 200 | 121,364 | 778 | 920 | 766,353 | 32,124,260 | 32,040,026 | 23.07 | 0.04 | SRR13054056 | mgl818007 | mgm4882938.3 |
| **Samp3_Stn7** | 0.22 | 515,190 | 398,991,752 | 200 | 238,672 | 774 | 909 | 795,285 | 34,428,046 | 34,308,366 | 16.29 | 0.04 | SRR13054055 | mgl818010 | mgm4882956.3 |
| **Samp3_Stn10** | 0.22 | 559,582 | 406,062,959 | 200 | 77,453 | 726 | 820 | 843,242 | 36,536,080 | 36,368,464 | 25.25 | 0.03 | SRR13054061 | mgl818004 | mgm4882968.3 |
| **Samp5_Stn1** | 0.22 | 597,030 | 436,773,626 | 200 | 140,955 | 732 | 837 | 893,175 | 47,263,132 | 47,081,744 | 26.42 | 0.08 | SRR13054054 | mgl818013 | mgm4882970.3 |
| **Samp5_Stn4** | 0.22 | 361,311 | 258,458,740 | 200 | 130,635 | 715 | 802 | 551,864 | 32,890,468 | 32,773,616 | 25.46 | 0.03 | SRR13054104 | mgl818019 | mgm4882939.3 |
| **Samp5_Stn7** | 0.22 | 470,957 | 352,276,317 | 200 | 173,418 | 748 | 859 | 730,096 | 32,195,330 | 32,098,010 | 30.09 | 0.03 | SRR13054103 | mgl818022 | mgm4882966.3 |
| **Samp5_Stn10** | 0.22 | 358,917 | 269,820,294 | 200 | 85,257 | 752 | 864 | 555,612 | 23,965,818 | 23,842,924 | 21.85 | 0.02 | SRR13054053 | mgl818016 | mgm4882952.3 |
| **Pre-Harvey1** | 0.22 | 494,328 | 364,865,701 | 200 | 55,138 | 738 | 825 | 735,220 | 27,887,626 | 27,791,264 | 18.22 | 0.03 | SRR13054102 | mgl818025 | mgm4882925.3 |
| **Pre-Harvey2** | 0.2 | 632,933 | 449,485,501 | 200 | 90,292 | 710 | 774 | 919,021 | 32,160,120 | 32,058,328 | 25.52 | 0.03 | SRR13054101 | mgl818028 | mgm4882955.3 |
| **Samp1_Stn1** | GFD | 481,796 | 295,863,296 | 200 | 322,552 | 614 | 624 | 624,959 | 27,011,362 | 26,930,666 | 17.69 | 0.12 | SRR13054096 | mgl818043 | mgm4882944.3 |
| **Samp1_Stn4** | GFD | 464,833 | 270,922,896 | 200 | 76,749 | 583 | 585 | 551,911 | 25,941,884 | 25,835,122 | 19.94 | 0.22 | SRR13054093 | mgl818049 | mgm4882948.3 |
| **Samp1_Stn7** | GFD | 442,933 | 252,155,932 | 200 | 39,597 | 569 | 566 | 556,460 | 26,548,726 | 26,487,858 | 26.68 | 0.47 | SRR13054092 | mgl818052 | mgm4882973.3 |
| **Samp1_Stn10** | GFD | 538,739 | 322,983,257 | 200 | 77,883 | 600 | 598 | 684,011 | 29,471,906 | 29,355,146 | 21.41 | 0.22 | SRR13054095 | mgl818046 | mgm4882941.3 |
| **Samp2_Stn1** | GFD | 504,945 | 310,570,014 | 200 | 52,301 | 615 | 624 | 651,039 | 34,538,226 | 34,415,282 | 17.8 | 0.33 | SRR13054091 | mgl818055 | mgm4882974.3 |
| **Samp2_Stn4** | GFD | 523,651 | 347,345,466 | 200 | 146,628 | 663 | 690 | 704,808 | 33,717,552 | 33,609,022 | 17.14 | 0.36 | SRR13054089 | mgl818061 | mgm4882929.3 |
| **Supplementary Table 3 (continued)** | | | | | | | | | | | | | | | |
| **Samp2_Stn7** | GFD | 645,360 | 408,224,781 | 200 | 60,818 | 633 | 644 | 827,671 | 33,263,434 | 33,139,334 | 21.47 | 0.22 | SRR13054088 | mgl818064 | mgm4882957.3 |
| **Samp2_Stn10** | GFD | 490,896 | 322,138,454 | 200 | 94,643 | 656 | 692 | 631,363 | 30,689,328 | 30,440,254 | 19.44 | 0.61 | SRR13054090 | mgl818058 | mgm4882937.3 |
| **Samp3_Stn1** | GFF | 635,532 | 487,604,658 | 200 | 86,627 | 767 | 892 | 937,146 | 40,496,102 | 40,394,972 | 14.81 | 0.16 | SRR13054087 | mgl818067 | mgm4882978.3 |
| **Samp3_Stn4** | GFF | 496,819 | 345,771,611 | 200 | 81,711 | 696 | 760 | 694,278 | 25,489,008 | 25,415,396 | 22.29 | 0.17 | SRR13054085 | mgl818073 | mgm4882947.3 |
| **Samp3_Stn7** | GFF | 531,449 | 351,935,643 | 200 | 149,754 | 662 | 697 | 758,212 | 27,893,754 | 27,822,518 | 24.1 | 0.18 | SRR13054084 | mgl818076 | mgm4882963.3 |
| **Samp3_Stn10** | GFF | 529,522 | 367,369,414 | 200 | 137,899 | 694 | 746 | 768,822 | 30,862,948 | 30,771,102 | 20.39 | 0.28 | SRR13054086 | mgl818070 | mgm4882932.3 |
| **Samp5_Stn1** | GFF | 472,772 | 321,751,838 | 200 | 138,312 | 681 | 726 | 669,782 | 30,082,442 | 29,971,260 | 20.83 | 0.37 | SRR13054082 | mgl818079 | mgm4882936.3 |
| **Samp5_Stn4** | GFF | 510,601 | 374,058,254 | 200 | 78,298 | 733 | 832 | 740,499 | 31,759,374 | 31,611,686 | 18.51 | 0.2 | SRR13054080 | mgl818085 | mgm4882933.3 |
| **Samp5_Stn7** | GFF | 467,757 | 356,100,990 | 200 | 106,610 | 761 | 891 | 693,354 | 31,518,434 | 31,396,792 | 17.6 | 0.19 | SRR13054079 | mgl818088 | mgm4882953.3 |
| **Samp5_Stn10** | GFF | 667,378 | 459,600,971 | 200 | 104,332 | 689 | 741 | 936,465 | 35,135,656 | 35,004,760 | 28.31 | 0.21 | SRR13054081 | mgl818082 | mgm4882945.3 |
| **Pre-Harvey1** | GFD | 652,489 | 438,889,672 | 200 | 89,952 | 673 | 717 | 876,799 | 39,073,962 | 38,934,916 | 15.04 | 0.27 | SRR13054078 | mgl818091 | mgm4882950.3 |
| **Pre-Harvey2** | 5µm | 519,384 | 324,961,850 | 200 | 89,811 | 626 | 649 | 670,489 | 34,793,716 | 34,652,358 | 16.97 | 0.4 | SRR13054077 | mgl818094 | mgm4882969.3 |
| **Co-Assembly** | All | 666,406 | 2,922,820,702 | 2,000 | 369,958 | 4,386 | 4,627 | 2,933,591 | NA | NA | NA | NA | NA | NA | NA |

**Supplementary Table 4:** Taxonomic classification of the MAGs from Kraken2, completeness and contamination estimates, and SSUs found by CheckM.

| **Bin ID** | **Karken 2 Taxonomic Classification** | **CheckM Lineage Results** | **CheckM SSU Find Results** |  |  |  |  |  |
| --- | --- | --- | --- | --- | --- | --- | --- | --- |
|  | **Domain** | **Phylum** | **Class** | **Completeness** | **Contamination** | **Total Length** | **Number Contigs** | **Note** |
| bin_1 | Bacteria | Bacteroidetes | Flavobacteriia | 56.37 | 0.59 | 1775252 | 571 | no_ssu |
| bin_10 | Bacteria | Cyanobacteria | NA | 52.99 | 4.31 | 1895994 | 485 | no_ssu |
| bin_100 | Bacteria | Actinobacteria | Actinobacteria | 69.28 | 23.43 | 1935609 | 425 | Uncultured bacterium clone 4 16S ribosomal RNA gene, partial sequence |
| bin_1000 | Bacteria | Proteobacteria | Alphaproteobacteria | 0 | 0 | 201782 | 59 | no_ssu |
| bin_1001 | Bacteria | Proteobacteria | Betaproteobacteria | 38.79 | 8.62 | 1111511 | 260 | no_ssu |
| bin_1002 | Bacteria | Firmicutes | Bacilli | 1.29 | 0.14 | 327793 | 84 | no_ssu |
| bin_1003 | Bacteria | Proteobacteria | Deltaproteobacteria | 86.29 | 2.42 | 4798208 | 730 | no_ssu |
| bin_1004 | Bacteria | Proteobacteria | Gammaproteobacteria | 22.47 | 3.38 | 1491167 | 533 | no_ssu |
| bin_1005 | Bacteria | Proteobacteria | Alphaproteobacteria | 99.09 | 26.16 | 3824162 | 391 | Oceanicola sp. LA276 16S ribosomal RNA gene, partial sequence |
| bin_1006 | Bacteria | Cyanobacteria | NA | 9.47 | 0.69 | 516835 | 191 | no_ssu |
| bin_1007 | Bacteria | Actinobacteria | Actinobacteria | 44.79 | 1.5 | 1124892 | 361 | Uncultured bacterium clone Wu-C35 16S ribosomal RNA gene, partial sequence |
| bin_1008 | Bacteria | Bacteroidetes | Flavobacteriia | 95.16 | 4.95 | 2923833 | 288 | no_ssu |
| bin_1009 | Bacteria | Proteobacteria | Gammaproteobacteria | 39.73 | 0 | 1061204 | 310 | no_ssu |
| bin_101 | NA | NA | NA | 4.01 | 0 | 355603 | 30 | no_ssu |
| bin_1010 | Bacteria | Actinobacteria | Actinobacteria | 71.28 | 42.24 | 1429627 | 430 | no_ssu |
| bin_1011 | Bacteria | Proteobacteria | Gammaproteobacteria | 4.17 | 0 | 460410 | 102 | no_ssu |
| bin_1012 | Bacteria | Proteobacteria | Gammaproteobacteria | 0 | 0 | 289772 | 69 | no_ssu |
| bin_1013 | Bacteria | Bacteroidetes | Flavobacteriia | 49.9 | 15.75 | 2262875 | 619 | no_ssu |
| bin_1014 | Bacteria | Firmicutes | Clostridia | 0 | 0 | 230030 | 52 | no_ssu |
| bin_1015 | Bacteria | Gemmatimonadetes | Gemmatimonadetes | 10.74 | 0 | 795607 | 301 | no_ssu |
| bin_1016 | Bacteria | Actinobacteria | Actinobacteria | 87.86 | 12.62 | 2346952 | 306 | Uncultured bacterium clone BJGMM-3s-275 16S ribosomal RNA gene, partial sequence |
| bin_1017 | Bacteria | Actinobacteria | Actinobacteria | 65.24 | 12.93 | 1123854 | 224 | no_ssu |
| bin_1018 | Bacteria | Actinobacteria | Actinobacteria | 52.3 | 21.55 | 874074 | 271 | no_ssu |
| bin_1019 | Bacteria | Firmicutes | Bacilli | 2.76 | 0 | 328326 | 79 | no_ssu |
| bin_102 | Bacteria | Proteobacteria | Alphaproteobacteria | 97 | 4.87 | 3132721 | 231 | Uncultured bacterium clone 3C003141 16S ribosomal RNA gene, partial sequence |
| bin_1020 | Bacteria | Actinobacteria | Actinobacteria | 82.95 | 1.36 | 1975792 | 308 | Uncultured marine bacterium clone S17-152 16S ribosomal RNA gene, partial sequence |
| bin_1021 | Bacteria | Proteobacteria | Alphaproteobacteria | 94.67 | 98.64 | 5247972 | 1199 | no_ssu |
| bin_1022 | Bacteria | Actinobacteria | Actinobacteria | 0 | 0 | 269287 | 98 | no_ssu |
| bin_1023 | Bacteria | Actinobacteria | Actinobacteria | 20.69 | 1.72 | 307639 | 91 | no_ssu |
| bin_1024 | Viruses | NA | NA | 6 | 0.86 | 469643 | 60 | no_ssu |
| bin_1025 | Bacteria | Cyanobacteria | NA | 4.31 | 0 | 508029 | 179 | no_ssu |
| bin_1026 | Bacteria | Bacteroidetes | Flavobacteriia | 0 | 0 | 217340 | 38 | no_ssu |
| bin_1027 | Bacteria | Proteobacteria | Gammaproteobacteria | 29.31 | 0 | 1231823 | 388 | no_ssu |
| bin_1028 | Bacteria | Proteobacteria | Alphaproteobacteria | 89.94 | 10.71 | 2722382 | 496 | Uncultured bacterium clone ML-7-9.2 16S ribosomal RNA gene, partial sequence |
| bin_1029 | Bacteria | Proteobacteria | Betaproteobacteria | 96.99 | 4.17 | 4663344 | 330 | Uncultured marine bacterium clone FJ-C41 16S ribosomal RNA gene, partial sequence |
| bin_103 | Bacteria | Actinobacteria | Actinobacteria | 83.05 | 2.14 | 1903184 | 316 | Uncultured Acidimicrobiales bacterium clone S2-9-069 16S ribosomal RNA gene, partial sequence |
| bin_1030 | Bacteria | Proteobacteria | Gammaproteobacteria | 5.25 | 0 | 1142489 | 230 | no_ssu |
| bin_1031 | Bacteria | Proteobacteria | Alphaproteobacteria | 7.64 | 0 | 429726 | 48 | no_ssu |
| bin_1032 | Bacteria | Firmicutes | Bacilli | 17.67 | 1.89 | 1432940 | 271 | no_ssu |
| bin_1033 | Bacteria | Firmicutes | Bacilli | 4.17 | 0 | 688773 | 172 | no_ssu |
| bin_1034 | Bacteria | Proteobacteria | Alphaproteobacteria | 50 | 3.45 | 1669282 | 483 | no_ssu |
| bin_1035 | Bacteria | Proteobacteria | Alphaproteobacteria | 16.61 | 0 | 515066 | 180 | no_ssu |
| bin_1036 | Bacteria | Proteobacteria | Gammaproteobacteria | 48.99 | 0.08 | 438355 | 81 | no_ssu |
| bin_1037 | Bacteria | Proteobacteria | Alphaproteobacteria | 0 | 0 | 657788 | 151 | no_ssu |
| bin_1038 | Bacteria | Cyanobacteria | NA | 4.46 | 0.14 | 351174 | 77 | no_ssu |
| bin_1039 | Bacteria | Actinobacteria | Actinobacteria | 95.91 | 7.36 | 3273787 | 282 | Uncultured bacterium clone BJGMM-3s-336 16S ribosomal RNA gene, partial sequence |
| bin_104 | Bacteria | Cyanobacteria | NA | 69.25 | 7.16 | 2256652 | 666 | no_ssu |
| bin_1040 | Bacteria | Cyanobacteria | NA | 65.83 | 9.24 | 2185190 | 616 | no_ssu |
| bin_1041 | Bacteria | Proteobacteria | Alphaproteobacteria | 6.9 | 0 | 203105 | 81 | no_ssu |
| bin_1042 | Bacteria | Bacteroidetes | Flavobacteriia | 94.58 | 14.1 | 4012143 | 759 | Uncultured bacterium clone MD164 16S ribosomal RNA gene, partial sequence |
| bin_1043 | Bacteria | Actinobacteria | Actinobacteria | 0 | 0 | 268165 | 9 | no_ssu |
| bin_1044 | Bacteria | Firmicutes | Bacilli | 6.31 | 0.58 | 678592 | 110 | no_ssu |
| bin_1045 | Bacteria | Proteobacteria | Gammaproteobacteria | 0 | 0 | 253258 | 99 | no_ssu |
| bin_1046 | Bacteria | Firmicutes | Clostridia | 0 | 0 | 426592 | 22 | no_ssu |
| bin_1047 | Bacteria | Cyanobacteria | NA | 1.72 | 0 | 266032 | 24 | no_ssu |
| bin_1048 | Bacteria | Firmicutes | Clostridia | 3.52 | 0 | 358979 | 87 | no_ssu |
| bin_1049 | Bacteria | Bacteroidetes | Flavobacteriia | 36.79 | 0 | 611663 | 216 | no_ssu |
| bin_105 | Bacteria | Actinobacteria | Actinobacteria | 63.88 | 10.08 | 1021129 | 173 | no_ssu |
| bin_1050 | Bacteria | Cyanobacteria | NA | 89.86 | 2.08 | 4230680 | 599 | no_ssu |
| bin_1051 | NA | NA | NA | 0 | 0 | 306201 | 13 | no_ssu |
| bin_1052 | Bacteria | Proteobacteria | Betaproteobacteria | 0 | 0 | 247997 | 52 | no_ssu |
| bin_1053 | Bacteria | Proteobacteria | Gammaproteobacteria | 62.88 | 0.34 | 1496243 | 386 | Buchnera aphidicola BCc, complete genome |
| bin_1054 | Bacteria | Bacteroidetes | Flavobacteriia | 94.32 | 5.05 | 2730567 | 358 | no_ssu |
| bin_1055 | Bacteria | Proteobacteria | Gammaproteobacteria | 12.73 | 0 | 836134 | 308 | no_ssu |
| bin_1056 | Bacteria | Proteobacteria | Gammaproteobacteria | 86.85 | 3.98 | 2805137 | 424 | Uncultured bacterium clone INDI_S_SPR_12E 16S ribosomal RNA gene, partial sequence |
| bin_1057 | Bacteria | Proteobacteria | Gammaproteobacteria | 0 | 0 | 309413 | 115 | no_ssu |
| bin_1058 | Bacteria | Proteobacteria | Gammaproteobacteria | 9.15 | 0.07 | 460054 | 52 | no_ssu |
| bin_1059 | Bacteria | Bacteroidetes | Flavobacteriia | 0 | 0 | 281196 | 100 | no_ssu |
| bin_106 | Bacteria | Proteobacteria | Alphaproteobacteria | 93.77 | 3.36 | 2289056 | 190 | Uncultured bacterium clone A20 16S ribosomal RNA gene, partial sequence |
| bin_1060 | Bacteria | Cyanobacteria | NA | 0 | 0 | 347617 | 91 | Halobacteriovorax sp. BALOs_7 chromosome, complete genome |
| bin_1061 | Bacteria | Proteobacteria | Alphaproteobacteria | 8.33 | 0 | 660317 | 197 | no_ssu |
| bin_1062 | Bacteria | Actinobacteria | Actinobacteria | 65.25 | 2.84 | 1390612 | 287 | no_ssu |
| bin_1063 | Bacteria | Proteobacteria | Betaproteobacteria | 100 | 107.35 | 6069871 | 1043 | no_ssu |
| bin_1064 | Bacteria | Bacteroidetes | Flavobacteriia | 67.75 | 3.92 | 1365125 | 329 | no_ssu |
| bin_1065 | Bacteria | Firmicutes | Bacilli | 5 | 0.5 | 340431 | 86 | no_ssu |
| bin_1066 | Bacteria | Actinobacteria | Actinobacteria | 17.43 | 0.58 | 310690 | 120 | no_ssu |
| bin_1067 | Bacteria | Proteobacteria | Gammaproteobacteria | 0 | 0 | 343299 | 35 | no_ssu |
| bin_1068 | Bacteria | Proteobacteria | Alphaproteobacteria | 59.54 | 2.43 | 2203632 | 660 | no_ssu |
| bin_1069 | Bacteria | Proteobacteria | Gammaproteobacteria | 24.14 | 0 | 1360197 | 417 | no_ssu |
| bin_107 | Bacteria | Actinobacteria | Actinobacteria | 60.74 | 14.18 | 2554459 | 676 | no_ssu |
| bin_1070 | Bacteria | Actinobacteria | Actinobacteria | 74.38 | 8 | 1318847 | 133 | no_ssu |
| bin_1071 | Bacteria | Actinobacteria | Actinobacteria | 86.99 | 68.92 | 3279216 | 479 | no_ssu |
| bin_1072 | Bacteria | Bacteroidetes | Flavobacteriia | 93.6 | 4.3 | 1769698 | 164 | Uncultured bacterium clone 3C003407 16S ribosomal RNA gene, partial sequence |
| bin_1073 | Bacteria | Firmicutes | Clostridia | 0 | 0 | 211969 | 42 | no_ssu |
| bin_1074 | Bacteria | Proteobacteria | Gammaproteobacteria | 16.38 | 1.72 | 411776 | 157 | no_ssu |
| bin_1075 | Bacteria | Proteobacteria | Gammaproteobacteria | 31.46 | 0.84 | 709653 | 247 | no_ssu |
| bin_1076 | Bacteria | Bacteroidetes | Chitinophagia | 95.57 | 0.49 | 2004545 | 69 | Uncultured bacterium clone C12 16S ribosomal RNA gene, partial sequence |
| bin_1077 | Bacteria | Bacteroidetes | Flavobacteriia | 8.65 | 0.58 | 746741 | 40 | no_ssu |
| bin_1078 | Bacteria | Bacteroidetes | Flavobacteriia | 98.44 | 5 | 2513213 | 259 | no_ssu |
| bin_1079 | Bacteria | Proteobacteria | Alphaproteobacteria | 37.93 | 5.17 | 1250284 | 425 | no_ssu |
| bin_108 | Bacteria | Cyanobacteria | Gloeobacteria | 3.04 | 0.81 | 439165 | 40 | no_ssu |
| bin_1080 | Bacteria | Proteobacteria | Betaproteobacteria | 16.67 | 0 | 1170479 | 335 | no_ssu |
| bin_1081 | Bacteria | Bacteroidetes | Flavobacteriia | 0 | 0 | 331134 | 61 | no_ssu |
| bin_1082 | Bacteria | Proteobacteria | Alphaproteobacteria | 9.8 | 0 | 1207463 | 398 | Thraustochytrium aureum mitochondrial DNA, partial genome |
| bin_1083 | Viruses | NA | NA | 0 | 0 | 205090 | 43 | no_ssu |
| bin_1084 | Bacteria | Bacteroidetes | Flavobacteriia | 0 | 0 | 208612 | 7 | no_ssu |
| bin_1085 | Bacteria | Proteobacteria | Alphaproteobacteria | 67.4 | 28.74 | 3964633 | 1310 | Uncultured bacterium clone 8S11 16S ribosomal RNA gene, partial sequence |
| bin_1086 | Bacteria | Proteobacteria | Alphaproteobacteria | 87.73 | 4.4 | 2928869 | 258 | no_ssu |
| bin_1087 | Bacteria | Firmicutes | Bacilli | 69.24 | 40.71 | 17810717 | 3998 | no_ssu |
| bin_1088 | Bacteria | Firmicutes | Clostridia | 5.38 | 1.26 | 596594 | 85 | no_ssu |
| bin_1089 | Bacteria | Proteobacteria | Betaproteobacteria | 4.62 | 1.29 | 417897 | 95 | no_ssu |
| bin_109 | Viruses | NA | NA | 0 | 0 | 340148 | 77 | no_ssu |
| bin_1090 | Bacteria | Bacteroidetes | Flavobacteriia | 73.95 | 4.97 | 1562922 | 311 | no_ssu |
| bin_1091 | Bacteria | Proteobacteria | Gammaproteobacteria | 96.07 | 0.56 | 6036888 | 295 | no_ssu |
| bin_1092 | Bacteria | Proteobacteria | Alphaproteobacteria | 17.41 | 0.47 | 1428740 | 521 | no_ssu |
| bin_1093 | Bacteria | Firmicutes | Bacilli | 0 | 0 | 316242 | 76 | no_ssu |
| bin_1094 | Bacteria | Actinobacteria | Actinobacteria | 76.97 | 3.35 | 2093519 | 439 | Uncultured bacterium clone LVB03B 16S ribosomal RNA gene, partial sequence |
| bin_1095 | Bacteria | Proteobacteria | Gammaproteobacteria | 0 | 0 | 227324 | 37 | no_ssu |
| bin_1096 | Bacteria | Proteobacteria | Gammaproteobacteria | 95.45 | 1.14 | 4584843 | 177 | Uncultured bacterium clone N4_106 16S ribosomal RNA gene, partial sequence |
| bin_1097 | Bacteria | Actinobacteria | Actinobacteria | 64.53 | 3.94 | 1577780 | 279 | Uncultured bacterium clone DP7.2.103 16S ribosomal RNA gene, partial sequence |
| bin_1098 | Bacteria | Firmicutes | Clostridia | 2.52 | 0 | 410844 | 69 | no_ssu |
| bin_1099 | Bacteria | Proteobacteria | Alphaproteobacteria | 93.13 | 3.31 | 4287632 | 464 | no_ssu |
| bin_11 | Bacteria | Firmicutes | Bacilli | 3.98 | 0.43 | 612485 | 61 | no_ssu |
| bin_110 | Bacteria | Proteobacteria | Gammaproteobacteria | 0 | 0 | 413590 | 139 | no_ssu |
| bin_1100 | Bacteria | Proteobacteria | Gammaproteobacteria | 15.85 | 0.33 | 1705671 | 487 | no_ssu |
| bin_1101 | Bacteria | Bacteroidetes | Flavobacteriia | 65.55 | 3.14 | 1390248 | 317 | Uncultured bacterium clone 224_304 16S ribosomal RNA gene, partial sequence |
| bin_1102 | Bacteria | Bacteroidetes | Flavobacteriia | 3.51 | 1.08 | 296314 | 61 | no_ssu |
| bin_1103 | Bacteria | Proteobacteria | Gammaproteobacteria | 26.65 | 6.9 | 980816 | 319 | no_ssu |
| bin_1104 | Bacteria | Proteobacteria | Alphaproteobacteria | 0 | 0 | 204337 | 34 | no_ssu |
| bin_1105 | Bacteria | Actinobacteria | Actinobacteria | 19.63 | 0 | 592205 | 169 | Uncultured bacterium clone B06LBA 16S ribosomal RNA gene, partial sequence |
| bin_1106 | Bacteria | Proteobacteria | Gammaproteobacteria | 0 | 0 | 243576 | 50 | no_ssu |
| bin_1107 | Bacteria | Proteobacteria | Gammaproteobacteria | 32.42 | 17.27 | 16489202 | 5154 | Uncultured bacterium clone E127_B03 16S ribosomal RNA gene, partial sequence |
| bin_1108 | Bacteria | Proteobacteria | Gammaproteobacteria | 52.8 | 4.49 | 1858225 | 546 | no_ssu |
| bin_1109 | Bacteria | Proteobacteria | Alphaproteobacteria | 21.47 | 0 | 623101 | 215 | no_ssu |
| bin_111 | Bacteria | Firmicutes | Bacilli | 0 | 0 | 299335 | 25 | no_ssu |
| bin_1110 | Bacteria | Firmicutes | Clostridia | 1.73 | 0 | 422920 | 78 | no_ssu |
| bin_1111 | Bacteria | Actinobacteria | Actinobacteria | 51.02 | 2.11 | 733383 | 148 | no_ssu |
| bin_1112 | Bacteria | Firmicutes | Bacilli | 58.57 | 3.88 | 1195659 | 318 | no_ssu |
| bin_1113 | Bacteria | Proteobacteria | Gammaproteobacteria | 5.44 | 0 | 370153 | 119 | no_ssu |
| bin_1114 | Bacteria | Proteobacteria | Gammaproteobacteria | 88.84 | 2.81 | 2802680 | 274 | no_ssu |
| bin_1115 | Bacteria | Verrucomicrobia | Opitutae | 94.21 | 1.51 | 2473372 | 271 | no_ssu |
| bin_1116 | Bacteria | Proteobacteria | Gammaproteobacteria | 86.77 | 5.02 | 2695264 | 327 | Uncultured bacterium clone T6_0611_49 16S ribosomal RNA gene, partial sequence |
| bin_1117 | Viruses | NA | NA | 0 | 0 | 355796 | 52 | no_ssu |
| bin_1118 | Bacteria | Firmicutes | Bacilli | 17.05 | 0 | 400886 | 139 | no_ssu |
| bin_1119 | Bacteria | Proteobacteria | Alphaproteobacteria | 85.97 | 9.45 | 1331286 | 252 | Rickettsiaceae endosymbiont of Carteria cerasiformis rrs, nlpD2 genes for MutT/nudix family protein, 16S ribosomal RNA, M23 superfamily membrane-bound metallopeptidase, complete cds |
| bin_112 | Bacteria | Firmicutes | Bacilli | 34.73 | 1.1 | 629418 | 210 | no_ssu |
| bin_1120 | Bacteria | Bacteroidetes | Flavobacteriia | 16.11 | 0 | 658283 | 229 | no_ssu |
| bin_1121 | Bacteria | Actinobacteria | Actinobacteria | 50.98 | 1.44 | 1401777 | 353 | Uncultured bacterium clone 2 16S ribosomal RNA gene, partial sequence |
| bin_1122 | Bacteria | Actinobacteria | Actinobacteria | 3.98 | 0.81 | 451895 | 30 | no_ssu |
| bin_1123 | Bacteria | Cyanobacteria | NA | 47.82 | 10.34 | 2149572 | 504 | no_ssu |
| bin_1124 | Bacteria | Bacteroidetes | Flavobacteriia | 81.97 | 0.9 | 2576653 | 495 | no_ssu |
| bin_1125 | Bacteria | Firmicutes | Bacilli | 0 | 0 | 216091 | 38 | no_ssu |
| bin_1126 | Bacteria | Proteobacteria | Alphaproteobacteria | 7.47 | 0.86 | 353704 | 141 | no_ssu |
| bin_1127 | Bacteria | Proteobacteria | Gammaproteobacteria | 0 | 0 | 388878 | 85 | no_ssu |
| bin_1128 | Archaea | Thaumarchaeota | NA | 76.24 | 1.29 | 1036774 | 157 | Candidatus Nitrosopumilus sp. SW chromosome, complete genome |
| bin_1129 | Bacteria | Bacteroidetes | Flavobacteriia | 0 | 0 | 238779 | 36 | no_ssu |
| bin_113 | Bacteria | Actinobacteria | Actinobacteria | 33.8 | 1.72 | 719590 | 194 | no_ssu |
| bin_1130 | Bacteria | Actinobacteria | Actinobacteria | 46.11 | 3.7 | 806866 | 248 | no_ssu |
| bin_1131 | Bacteria | Proteobacteria | Alphaproteobacteria | 81.28 | 138.9 | 13994024 | 3668 | no_ssu |
| bin_1132 | Bacteria | Proteobacteria | Gammaproteobacteria | 0 | 0 | 230058 | 43 | no_ssu |
| bin_1133 | Bacteria | Proteobacteria | Alphaproteobacteria | 89.38 | 4.56 | 3493317 | 350 | no_ssu |
| bin_1134 | Bacteria | Bacteroidetes | Flavobacteriia | 2.73 | 0 | 285778 | 13 | no_ssu |
| bin_1135 | Bacteria | Proteobacteria | Gammaproteobacteria | 34.04 | 3.51 | 2629080 | 794 | Uncultured bacterium partial 16S rRNA gene, clone WK60H42 |
| bin_1136 | Bacteria | Proteobacteria | Alphaproteobacteria | 94.76 | 7.26 | 7158344 | 801 | Uncultured bacterium gene for 16S rRNA, partial sequence, clone: RBC3-48 |
| bin_1137 | Bacteria | Firmicutes | Bacilli | 13.01 | 0 | 693030 | 171 | no_ssu |
| bin_1138 | Bacteria | Actinobacteria | Actinobacteria | 59.82 | 0.6 | 1668303 | 318 | no_ssu |
| bin_1139 | NA | NA | NA | 0.07 | 0 | 253170 | 28 | no_ssu |
| bin_114 | Bacteria | Firmicutes | Clostridia | 5.78 | 0.47 | 464906 | 60 | no_ssu |
| bin_1140 | Bacteria | Actinobacteria | Actinobacteria | 39.84 | 0 | 859260 | 274 | Uncultured bacterium clone 14 16S ribosomal RNA gene, partial sequence |
| bin_1141 | Bacteria | Actinobacteria | Actinobacteria | 95.7 | 2.83 | 3654886 | 78 | Unidentified marine bacterioplankton clone P4-2B_23 16S ribosomal RNA gene, partial sequence |
| bin_1142 | Bacteria | Proteobacteria | Gammaproteobacteria | 24.03 | 0.89 | 1189904 | 418 | no_ssu |
| bin_1143 | Bacteria | Actinobacteria | Actinobacteria | 26.72 | 0 | 399381 | 106 | no_ssu |
| bin_1144 | Bacteria | Proteobacteria | Alphaproteobacteria | 92.87 | 8.41 | 3174009 | 356 | no_ssu |
| bin_1145 | Bacteria | Proteobacteria | Alphaproteobacteria | 57.52 | 18.97 | 3714901 | 1205 | no_ssu |
| bin_1146 | Bacteria | Proteobacteria | Alphaproteobacteria | 63.23 | 2.49 | 3049766 | 744 | no_ssu |
| bin_1147 | Bacteria | Bacteroidetes | Flavobacteriia | 97.46 | 4.64 | 2943363 | 214 | no_ssu |
| bin_1148 | Bacteria | Bacteroidetes | Flavobacteriia | 39.68 | 0 | 1026235 | 305 | no_ssu |
| bin_1149 | Bacteria | Proteobacteria | Alphaproteobacteria | 50.4 | 7.83 | 541112 | 135 | no_ssu |
| bin_115 | NA | NA | NA | 1.1 | 0.36 | 276636 | 11 | no_ssu |
| bin_1150 | Bacteria | Cyanobacteria | NA | 6.9 | 1.72 | 453901 | 137 | no_ssu |
| bin_1151 | Bacteria | Proteobacteria | Gammaproteobacteria | 79.78 | 1.5 | 1592370 | 322 | Leptocylindrus danicus chloroplast, complete genome |
| bin_1152 | Bacteria | Proteobacteria | Alphaproteobacteria | 64.23 | 11.26 | 3707490 | 885 | Breoghania sp. L-A4 chromosome, complete genome |
| bin_1153 | Bacteria | Actinobacteria | Actinobacteria | 50.52 | 1.72 | 715991 | 202 | no_ssu |
| bin_1154 | Bacteria | Proteobacteria | Alphaproteobacteria | 0 | 0 | 404242 | 162 | no_ssu |
| bin_1155 | Bacteria | Proteobacteria | Gammaproteobacteria | 23.68 | 3.51 | 405670 | 136 | no_ssu |
| bin_1156 | Bacteria | Proteobacteria | Betaproteobacteria | 74.78 | 3.6 | 3252598 | 791 | no_ssu |
| bin_1157 | Bacteria | Proteobacteria | Gammaproteobacteria | 82.13 | 14.33 | 2931675 | 660 | no_ssu |
| bin_1158 | Bacteria | Proteobacteria | Gammaproteobacteria | 0 | 0 | 231559 | 37 | no_ssu |
| bin_1159 | Bacteria | Cyanobacteria | NA | 5.38 | 0.29 | 469677 | 103 | no_ssu |
| bin_116 | Bacteria | Proteobacteria | Gammaproteobacteria | 91.63 | 60.31 | 5045733 | 1340 | no_ssu |
| bin_1160 | Bacteria | Proteobacteria | Gammaproteobacteria | 14.04 | 2.27 | 6059372 | 2227 | no_ssu |
| bin_1161 | Bacteria | Proteobacteria | Gammaproteobacteria | 51.29 | 1.68 | 1935972 | 607 | no_ssu |
| bin_1162 | Bacteria | Firmicutes | Clostridia | 3.98 | 0 | 313135 | 23 | no_ssu |
| bin_1163 | Bacteria | Proteobacteria | Alphaproteobacteria | 16.38 | 3.45 | 685745 | 230 | no_ssu |
| bin_1164 | Bacteria | Cyanobacteria | NA | 0 | 0 | 217794 | 9 | no_ssu |
| bin_1165 | Bacteria | Cyanobacteria | NA | 4.17 | 0 | 232479 | 62 | no_ssu |
| bin_1166 | Bacteria | Proteobacteria | Alphaproteobacteria | 25.22 | 0 | 353688 | 97 | no_ssu |
| bin_1167 | Bacteria | Proteobacteria | Alphaproteobacteria | 49.99 | 0 | 500836 | 139 | no_ssu |
| bin_1168 | Bacteria | Proteobacteria | Gammaproteobacteria | 0 | 0 | 304162 | 79 | no_ssu |
| bin_1169 | Bacteria | Proteobacteria | Betaproteobacteria | 0 | 0 | 256496 | 44 | no_ssu |
| bin_117 | Bacteria | Actinobacteria | Actinobacteria | 25 | 1.72 | 717340 | 225 | no_ssu |
| bin_1170 | Bacteria | Proteobacteria | Alphaproteobacteria | 0 | 0 | 393823 | 12 | no_ssu |
| bin_1171 | Bacteria | Proteobacteria | Gammaproteobacteria | 6.03 | 0 | 498186 | 180 | no_ssu |
| bin_1172 | Bacteria | Actinobacteria | Actinobacteria | 58.7 | 5.17 | 1502534 | 373 | no_ssu |
| bin_1173 | Bacteria | Proteobacteria | Gammaproteobacteria | 84.68 | 0.84 | 3198641 | 205 | Uncultured bacterium clone F3041 16S ribosomal RNA gene, partial sequence |
| bin_1174 | Bacteria | Firmicutes | Bacilli | 0 | 0 | 221193 | 29 | no_ssu |
| bin_1175 | Bacteria | Proteobacteria | Gammaproteobacteria | 92.44 | 1.2 | 1795607 | 233 | Uncultured bacterium clone H2_10.4_2 16S ribosomal RNA gene, partial sequence |
| bin_1176 | Bacteria | Proteobacteria | Alphaproteobacteria | 79.57 | 3.12 | 2076228 | 269 | Uncultured bacterium GRIST12 genomic sequence |
| bin_1177 | Bacteria | Bacteroidetes | Flavobacteriia | 98.39 | 1.52 | 2506228 | 203 | Uncultured marine bacterium clone GD-C14 16S ribosomal RNA gene, partial sequence |
| bin_1178 | Bacteria | Proteobacteria | Betaproteobacteria | 20.34 | 0 | 306996 | 91 | no_ssu |
| bin_1179 | Bacteria | Bacteroidetes | Flavobacteriia | 89.15 | 1.16 | 1608688 | 182 | no_ssu |
| bin_118 | Bacteria | Firmicutes | Bacilli | 59.19 | 24.69 | 1496755 | 420 | no_ssu |
| bin_1180 | Bacteria | Proteobacteria | Gammaproteobacteria | 39 | 0.03 | 730692 | 219 | no_ssu |
| bin_1181 | Bacteria | Cyanobacteria | NA | 0 | 0 | 611265 | 155 | no_ssu |
| bin_1182 | Bacteria | Proteobacteria | Alphaproteobacteria | 92.42 | 2.37 | 1008957 | 101 | Uncultured bacterium clone Woods-Hole_a3703 16S ribosomal RNA gene, partial sequence |
| bin_1183 | Bacteria | Proteobacteria | Gammaproteobacteria | 0 | 0 | 278442 | 25 | no_ssu |
| bin_1184 | Bacteria | Cyanobacteria | NA | 13.79 | 0 | 422444 | 158 | no_ssu |
| bin_1185 | Bacteria | Bacteroidetes | Bacteroidia | 0 | 0 | 273964 | 34 | no_ssu |
| bin_1186 | Bacteria | Proteobacteria | Gammaproteobacteria | 28.87 | 2.2 | 622936 | 202 | no_ssu |
| bin_1187 | Bacteria | Proteobacteria | Gammaproteobacteria | 6.31 | 0.93 | 850840 | 123 | no_ssu |
| bin_1188 | Bacteria | Proteobacteria | Gammaproteobacteria | 32.05 | 0 | 1582054 | 342 | no_ssu |
| bin_1189 | Bacteria | Proteobacteria | Gammaproteobacteria | 3.51 | 0.81 | 357029 | 9 | no_ssu |
| bin_119 | Bacteria | Firmicutes | Bacilli | 6.78 | 1.19 | 557752 | 79 | no_ssu |
| bin_1190 | Bacteria | Proteobacteria | Alphaproteobacteria | 2.63 | 0 | 650408 | 186 | no_ssu |
| bin_1191 | Bacteria | Proteobacteria | Betaproteobacteria | 74.92 | 41.65 | 2036325 | 478 | no_ssu |
| bin_1192 | Bacteria | Proteobacteria | Betaproteobacteria | 90.36 | 1.48 | 2753620 | 208 | no_ssu |
| bin_1193 | Bacteria | Actinobacteria | Actinobacteria | 51.03 | 1.72 | 1085670 | 280 | no_ssu |
| bin_1194 | Bacteria | Proteobacteria | Alphaproteobacteria | 16.12 | 2.06 | 413182 | 113 | no_ssu |
| bin_1195 | Bacteria | Proteobacteria | Gammaproteobacteria | 86.56 | 6.49 | 3467968 | 269 | Thiohalobacter thiocyanaticus DNA, complete genome, strain: FOKN1 |
| bin_1196 | Bacteria | Bacteroidetes | Flavobacteriia | 5.99 | 0 | 497364 | 169 | no_ssu |
| bin_1197 | Bacteria | Bacteroidetes | Flavobacteriia | 97.3 | 1.64 | 2569345 | 213 | no_ssu |
| bin_1198 | Bacteria | Firmicutes | Bacilli | 10.52 | 0.29 | 831613 | 218 | no_ssu |
| bin_1199 | Bacteria | Proteobacteria | Alphaproteobacteria | 0 | 0 | 321127 | 103 | no_ssu |
| bin_12 | Bacteria | Proteobacteria | Epsilonproteobacteria | 0.22 | 0 | 411932 | 88 | no_ssu |
| bin_120 | Bacteria | Proteobacteria | Gammaproteobacteria | 8.33 | 0 | 384197 | 148 | no_ssu |
| bin_1200 | Bacteria | Bacteroidetes | Flavobacteriia | 8.62 | 0 | 211511 | 49 | no_ssu |
| bin_1201 | Bacteria | Proteobacteria | Alphaproteobacteria | 13.79 | 0 | 902618 | 309 | no_ssu |
| bin_1202 | Bacteria | Proteobacteria | Gammaproteobacteria | 65.62 | 0.07 | 555513 | 104 | no_ssu |
| bin_1203 | Bacteria | Bacteroidetes | Flavobacteriia | 17.59 | 0 | 360178 | 138 | no_ssu |
| bin_1204 | Bacteria | Proteobacteria | Alphaproteobacteria | 0 | 0 | 223307 | 30 | no_ssu |
| bin_1205 | Bacteria | Proteobacteria | Alphaproteobacteria | 0 | 0 | 329637 | 5 | no_ssu |
| bin_1206 | Bacteria | Bacteroidetes | Flavobacteriia | 75.86 | 17.55 | 1907618 | 421 | no_ssu |
| bin_1207 | Bacteria | Bacteroidetes | Flavobacteriia | 4.17 | 0 | 324646 | 83 | no_ssu |
| bin_1208 | Viruses | NA | NA | 0 | 0 | 354578 | 86 | no_ssu |
| bin_1209 | Bacteria | Proteobacteria | Gammaproteobacteria | 76.7 | 3.11 | 2570256 | 414 | no_ssu |
| bin_121 | Bacteria | Firmicutes | Clostridia | 4.91 | 0.79 | 488566 | 29 | no_ssu |
| bin_1210 | Bacteria | Proteobacteria | Alphaproteobacteria | 25.76 | 0 | 907715 | 276 | no_ssu |
| bin_1211 | Bacteria | Proteobacteria | Gammaproteobacteria | 38.56 | 0 | 2600447 | 746 | no_ssu |
| bin_1212 | Bacteria | Proteobacteria | Gammaproteobacteria | 0 | 0 | 321677 | 89 | no_ssu |
| bin_1213 | Bacteria | Bacteroidetes | Flavobacteriia | 97.78 | 1.62 | 2195490 | 182 | Glaciecola sp. THG-3.7 chromosome, complete genome |
| bin_1214 | Bacteria | Cyanobacteria | NA | 0.78 | 0 | 272688 | 67 | no_ssu |
| bin_1215 | Bacteria | Actinobacteria | NA | 80.45 | 1.58 | 1234366 | 149 | Actinobacteria bacterium IMCC26077, complete genome |
| bin_1216 | Bacteria | Proteobacteria | Gammaproteobacteria | 88.72 | 3.96 | 2344519 | 241 | Uncultured gamma proteobacterium clone CB11C09 16S ribosomal RNA gene, partial sequence |
| bin_1217 | Bacteria | Bacteroidetes | Flavobacteriia | 52.46 | 1.2 | 864594 | 252 | no_ssu |
| bin_1218 | Bacteria | Proteobacteria | Gammaproteobacteria | 81.02 | 3.15 | 2785241 | 497 | Leifsonia sp. strain H8OOH1 16S ribosomal RNA gene, partial sequence |
| bin_1219 | Bacteria | Bacteroidetes | Flavobacteriia | 97.54 | 1.31 | 2407673 | 97 | Uncultured bacterium clone S0122 16S ribosomal RNA gene, partial sequence |
| bin_122 | Bacteria | Proteobacteria | Alphaproteobacteria | 4.17 | 0 | 1937739 | 487 | Actinobacteria bacterium strain LSUCC0392 16S ribosomal RNA gene, partial sequence |
| bin_1220 | Bacteria | Proteobacteria | Betaproteobacteria | 96.11 | 2.45 | 3041603 | 248 | Sutterella sp. strain ASD3426 16S ribosomal RNA gene, partial sequence |
| bin_1221 | Bacteria | Proteobacteria | Betaproteobacteria | 88.97 | 17.09 | 1447633 | 185 | no_ssu |
| bin_1222 | Bacteria | Actinobacteria | Actinobacteria | 2.87 | 0.57 | 331570 | 86 | no_ssu |
| bin_1223 | Bacteria | Proteobacteria | Alphaproteobacteria | 93.18 | 4.24 | 3458137 | 350 | Uncultured bacterium clone Contig\41 16S ribosomal RNA gene, partial sequence |
| bin_1224 | Bacteria | Actinobacteria | Actinobacteria | 39.57 | 1.89 | 532781 | 169 | no_ssu |
| bin_1225 | Bacteria | Proteobacteria | Alphaproteobacteria | 84.28 | 19.86 | 2206310 | 386 | Leisingera aquaemixtae strain R2C4 chromosome, complete genome |
| bin_1226 | Bacteria | Proteobacteria | Alphaproteobacteria | 75.25 | 83.49 | 6346928 | 1772 | no_ssu |
| bin_1227 | Bacteria | Proteobacteria | Alphaproteobacteria | 93.22 | 36.26 | 2989522 | 395 | Uncultured bacterium clone NN83 16S ribosomal RNA gene, partial sequence |
| bin_1228 | Bacteria | Proteobacteria | Alphaproteobacteria | 82.46 | 72.46 | 7292655 | 1330 | no_ssu |
| bin_1229 | Bacteria | Actinobacteria | Actinobacteria | 3.97 | 0 | 268139 | 103 | no_ssu |
| bin_123 | Bacteria | Firmicutes | Clostridia | 4.84 | 0.5 | 435324 | 19 | no_ssu |
| bin_1230 | Bacteria | Proteobacteria | Alphaproteobacteria | 0 | 0 | 275784 | 19 | no_ssu |
| bin_1231 | Bacteria | Proteobacteria | Gammaproteobacteria | 84.68 | 3.54 | 1981629 | 356 | Uncultured bacterium clone B08-148-BAC 16S ribosomal RNA gene, partial sequence |
| bin_1232 | Bacteria | Proteobacteria | Alphaproteobacteria | 88.52 | 47.21 | 3720053 | 850 | no_ssu |
| bin_1233 | Bacteria | Cyanobacteria | NA | 49.27 | 2.75 | 1815909 | 538 | no_ssu |
| bin_1234 | Bacteria | Proteobacteria | Gammaproteobacteria | 0 | 0 | 273173 | 65 | no_ssu |
| bin_1235 | Viruses | NA | NA | 0 | 0 | 882088 | 239 | no_ssu |
| bin_1236 | Bacteria | Proteobacteria | Alphaproteobacteria | 62.43 | 6.42 | 1689835 | 425 | no_ssu |
| bin_1237 | Bacteria | Proteobacteria | Gammaproteobacteria | 83.78 | 83.24 | 4029309 | 1029 | Halomonas elongata DSM 2581, complete genome |
| bin_1238 | Bacteria | Proteobacteria | Alphaproteobacteria | 52.16 | 2.27 | 2234912 | 569 | no_ssu |
| bin_1239 | Bacteria | Proteobacteria | Gammaproteobacteria | 7.25 | 0.29 | 394607 | 56 | no_ssu |
| bin_124 | Bacteria | Actinobacteria | Actinobacteria | 91.63 | 57.05 | 4814638 | 1019 | no_ssu |
| bin_1240 | Bacteria | Proteobacteria | Gammaproteobacteria | 56.14 | 20.69 | 2146358 | 547 | no_ssu |
| bin_1241 | Bacteria | Cyanobacteria | NA | 82.8 | 6.88 | 2321266 | 392 | no_ssu |
| bin_1242 | Bacteria | Actinobacteria | Actinobacteria | 46.98 | 35.34 | 1976339 | 584 | no_ssu |
| bin_1243 | Bacteria | Bacteroidetes | Flavobacteriia | 51.72 | 18.1 | 1593109 | 462 | no_ssu |
| bin_1244 | Bacteria | Proteobacteria | Deltaproteobacteria | 1.72 | 0.16 | 964636 | 266 | no_ssu |
| bin_1245 | Bacteria | Firmicutes | Bacilli | 1.94 | 0 | 507031 | 122 | Porphyridium sordidum plastid, complete genome |
| bin_1246 | Bacteria | Actinobacteria | Actinobacteria | 73.98 | 0.85 | 1650934 | 295 | no_ssu |
| bin_1247 | Bacteria | Actinobacteria | Actinobacteria | 1.51 | 0 | 245015 | 24 | no_ssu |
| bin_1248 | Bacteria | Proteobacteria | Betaproteobacteria | 6.25 | 9.95 | 1003511 | 240 | no_ssu |
| bin_1249 | Bacteria | Actinobacteria | Actinobacteria | 1.64 | 0.31 | 708406 | 55 | no_ssu |
| bin_125 | Bacteria | Proteobacteria | Gammaproteobacteria | 92.61 | 0.1 | 2931692 | 399 | no_ssu |
| bin_1250 | Bacteria | Actinobacteria | Actinobacteria | 17.69 | 0 | 395659 | 150 | no_ssu |
| bin_1251 | Bacteria | Proteobacteria | Gammaproteobacteria | 4.16 | 0.93 | 389153 | 103 | no_ssu |
| bin_1252 | Bacteria | Proteobacteria | Gammaproteobacteria | 0 | 0 | 234965 | 48 | no_ssu |
| bin_1253 | Bacteria | Cyanobacteria | NA | 11.3 | 0.59 | 236562 | 46 | Porphyridium purpureum culture CCMP:1328 plastid, complete genome |
| bin_1254 | Bacteria | Proteobacteria | Gammaproteobacteria | 3.44 | 0.29 | 391028 | 68 | no_ssu |
| bin_1255 | Bacteria | Proteobacteria | Betaproteobacteria | 4.27 | 0 | 687173 | 161 | no_ssu |
| bin_1256 | Bacteria | Actinobacteria | Actinobacteria | 42.16 | 1.28 | 865321 | 239 | Uncultured marine bacterium clone FJ-C11 16S ribosomal RNA gene, partial sequence |
| bin_1257 | Bacteria | Bacteroidetes | Cytophagia | 38.79 | 0 | 1290109 | 433 | no_ssu |
| bin_1258 | Bacteria | Proteobacteria | Betaproteobacteria | 7.11 | 1.29 | 1165838 | 367 | no_ssu |
| bin_1259 | Bacteria | Proteobacteria | Gammaproteobacteria | 0 | 0 | 240492 | 37 | no_ssu |
| bin_126 | Bacteria | Bacteroidetes | Flavobacteriia | 1.68 | 1.08 | 475078 | 163 | no_ssu |
| bin_1260 | Bacteria | Proteobacteria | Alphaproteobacteria | 5.85 | 0 | 630191 | 84 | no_ssu |
| bin_1261 | Bacteria | Proteobacteria | Alphaproteobacteria | 0 | 0 | 331833 | 78 | no_ssu |
| bin_1262 | Bacteria | Firmicutes | Clostridia | 5.38 | 0.5 | 389054 | 51 | no_ssu |
| bin_1263 | Bacteria | Proteobacteria | Gammaproteobacteria | 0 | 0 | 243461 | 29 | no_ssu |
| bin_1264 | Bacteria | Firmicutes | Bacilli | 4.45 | 1.51 | 442982 | 35 | no_ssu |
| bin_1265 | Bacteria | Firmicutes | Clostridia | 4.91 | 0.5 | 449763 | 30 | no_ssu |
| bin_1266 | Bacteria | Proteobacteria | Alphaproteobacteria | 3.98 | 0.5 | 473069 | 73 | no_ssu |
| bin_1267 | Bacteria | Bacteroidetes | Flavobacteriia | 0 | 0 | 418291 | 102 | no_ssu |
| bin_1268 | Bacteria | Proteobacteria | Alphaproteobacteria | 27.51 | 1.54 | 1133376 | 403 | no_ssu |
| bin_1269 | Bacteria | Proteobacteria | Gammaproteobacteria | 0 | 0 | 319061 | 113 | no_ssu |
| bin_127 | Bacteria | Bacteroidetes | Cytophagia | 0 | 0 | 211848 | 39 | no_ssu |
| bin_1270 | Bacteria | Bacteroidetes | Flavobacteriia | 96.19 | 0 | 2225342 | 109 | no_ssu |
| bin_1271 | Bacteria | Bacteroidetes | Flavobacteriia | 96.04 | 2.15 | 2401481 | 82 | no_ssu |
| bin_1272 | Bacteria | Actinobacteria | Actinobacteria | 66.43 | 3.78 | 1475824 | 423 | no_ssu |
| bin_1273 | Bacteria | Firmicutes | Bacilli | 4.45 | 1.51 | 506500 | 102 | no_ssu |
| bin_1274 | Bacteria | Firmicutes | Bacilli | 5.09 | 0.5 | 392081 | 52 | no_ssu |
| bin_1275 | Bacteria | Proteobacteria | Gammaproteobacteria | 5.26 | 0 | 682136 | 245 | Uncultured bacterium clone EB6_3cmOrig-B11 16S ribosomal RNA gene, partial sequence |
| bin_1276 | Bacteria | Proteobacteria | Gammaproteobacteria | 0 | 0 | 233388 | 34 | no_ssu |
| bin_1277 | Bacteria | Proteobacteria | Betaproteobacteria | 50.85 | 19.83 | 2879632 | 887 | no_ssu |
| bin_1278 | Bacteria | Proteobacteria | Alphaproteobacteria | 96.02 | 3.39 | 2973192 | 288 | no_ssu |
| bin_1279 | Bacteria | Proteobacteria | Alphaproteobacteria | 93.58 | 1.44 | 2648526 | 270 | Roseibacterium elongatum DSM 19469, complete genome |
| bin_128 | Bacteria | Proteobacteria | Alphaproteobacteria | 41.17 | 6.89 | 715076 | 121 | no_ssu |
| bin_1280 | Bacteria | Actinobacteria | Actinobacteria | 35 | 5.17 | 1886133 | 639 | no_ssu |
| bin_1281 | Bacteria | Proteobacteria | Gammaproteobacteria | 6.07 | 2.54 | 361825 | 50 | no_ssu |
| bin_1282 | Bacteria | Proteobacteria | Gammaproteobacteria | 25.17 | 8.62 | 1563114 | 522 | no_ssu |
| bin_1283 | Bacteria | Proteobacteria | Betaproteobacteria | 0 | 0 | 264994 | 66 | no_ssu |
| bin_1284 | Bacteria | Actinobacteria | Actinobacteria | 0 | 0 | 232721 | 31 | no_ssu |
| bin_1285 | Bacteria | Proteobacteria | Alphaproteobacteria | 70.01 | 3.92 | 1991741 | 518 | no_ssu |
| bin_1286 | Bacteria | Proteobacteria | Gammaproteobacteria | 15.79 | 0 | 1006690 | 331 | no_ssu |
| bin_1287 | Bacteria | Proteobacteria | Betaproteobacteria | 40.82 | 2.25 | 1449520 | 475 | Uncultured bacterium partial 16S rRNA gene, clone Iron-rich microbial mat clone Hoffnungsstollen_#4-2_B03 |
| bin_1288 | Bacteria | Proteobacteria | Betaproteobacteria | 49.62 | 45.86 | 1969512 | 500 | Polynucleobacter difficilis strain AM-8B5 chromosome, complete genome |
| bin_1289 | Bacteria | Firmicutes | Bacilli | 52.59 | 1.72 | 642736 | 185 | no_ssu |
| bin_129 | Bacteria | Verrucomicrobia | Opitutae | 71.54 | 8.8 | 4239195 | 900 | Uncultured bacterium clone NC89 16S ribosomal RNA gene, partial sequence |
| bin_1290 | Bacteria | Firmicutes | Bacilli | 5.31 | 3.52 | 623472 | 129 | no_ssu |
| bin_1291 | Bacteria | Proteobacteria | Alphaproteobacteria | 62.75 | 13.87 | 4069254 | 1160 | Uncultured bacterium gene for 16S rRNA, partial sequence, clone: Baqar.Sed.Eubac.12 |
| bin_1292 | Bacteria | Bacteroidetes | Flavobacteriia | 0 | 0 | 376057 | 44 | no_ssu |
| bin_1293 | Bacteria | Bacteroidetes | Flavobacteriia | 82.23 | 3.41 | 1682366 | 315 | no_ssu |
| bin_1294 | Bacteria | Actinobacteria | Actinobacteria | 88.89 | 6.41 | 2272070 | 142 | Uncultured Acidimicrobiales bacterium clone OTU187 16S ribosomal RNA gene, partial sequence |
| bin_1295 | Bacteria | Proteobacteria | Gammaproteobacteria | 40.1 | 2.61 | 2020707 | 664 | no_ssu |
| bin_1296 | Bacteria | Firmicutes | Clostridia | 4.6 | 0.07 | 300328 | 73 | no_ssu |
| bin_1297 | Bacteria | Proteobacteria | Alphaproteobacteria | 85.96 | 8.71 | 1777460 | 401 | Uncultured alpha proteobacterium HF0070_34A12 genomic sequence |
| bin_1298 | Bacteria | Proteobacteria | Alphaproteobacteria | 33.28 | 1.72 | 1468735 | 503 | no_ssu |
| bin_1299 | Bacteria | Actinobacteria | Actinobacteria | 4.17 | 0 | 514599 | 127 | no_ssu |
| bin_13 | Bacteria | Proteobacteria | Gammaproteobacteria | 38.55 | 1.42 | 1286212 | 390 | Uncultured bacterium clone C41 16S ribosomal RNA gene, partial sequence |
| bin_130 | Bacteria | Proteobacteria | Gammaproteobacteria | 86.89 | 7.64 | 4489243 | 668 | Uncultured bacterium clone ST27_5m_clone15 16S ribosomal RNA gene, partial sequence |
| bin_1300 | Bacteria | Proteobacteria | Gammaproteobacteria | 17.61 | 0.4 | 391973 | 141 | no_ssu |
| bin_1301 | Bacteria | Cyanobacteria | NA | 8.33 | 0 | 981126 | 306 | no_ssu |
| bin_1302 | Bacteria | Firmicutes | Clostridia | 0 | 0 | 373932 | 65 | no_ssu |
| bin_1303 | Bacteria | Proteobacteria | Alphaproteobacteria | 96.52 | 1.66 | 1523705 | 52 | Uncultured bacterium clone S25_780 16S ribosomal RNA gene, partial sequence |
| bin_1304 | Bacteria | Proteobacteria | Alphaproteobacteria | 88.7 | 4.25 | 3128696 | 516 | no_ssu |
| bin_1305 | Bacteria | Proteobacteria | Alphaproteobacteria | 95.77 | 76.94 | 5109856 | 825 | Sphingomonas sp. W1-2-3 chromosome, complete genome |
| bin_1306 | Bacteria | Actinobacteria | Actinobacteria | 88.84 | 7.68 | 1488479 | 206 | no_ssu |
| bin_1307 | Bacteria | Verrucomicrobia | Opitutae | 75.47 | 13.53 | 2714049 | 378 | Nibricoccus aquaticus strain HZ-65 chromosome, complete genome |
| bin_1308 | Bacteria | Bacteroidetes | Flavobacteriia | 4.17 | 0 | 309993 | 94 | no_ssu |
| bin_1309 | Bacteria | Cyanobacteria | NA | 5.61 | 0.27 | 1428443 | 425 | Uncultured organism clone 051011_T2S1_W_T_SDP_100 small subunit ribosomal RNA gene, partial sequence; mitochondrial |
| bin_131 | Bacteria | Proteobacteria | Gammaproteobacteria | 5.43 | 0 | 1199330 | 417 | no_ssu |
| bin_1310 | Bacteria | Proteobacteria | Alphaproteobacteria | 74.25 | 3.95 | 2198722 | 469 | no_ssu |
| bin_1311 | Bacteria | Proteobacteria | Alphaproteobacteria | 59.39 | 0 | 1710899 | 475 | no_ssu |
| bin_1312 | Bacteria | Proteobacteria | Betaproteobacteria | 65.71 | 1.38 | 1658236 | 373 | no_ssu |
| bin_1313 | Bacteria | Proteobacteria | Alphaproteobacteria | 22.41 | 1.72 | 260825 | 66 | no_ssu |
| bin_1314 | Bacteria | Firmicutes | Bacilli | 94.11 | 5.9 | 1115313 | 116 | no_ssu |
| bin_1315 | Bacteria | Firmicutes | Clostridia | 6.31 | 0.5 | 365358 | 31 | no_ssu |
| bin_1316 | Bacteria | Bacteroidetes | Flavobacteriia | 0 | 0 | 370431 | 62 | no_ssu |
| bin_1317 | Bacteria | Proteobacteria | Gammaproteobacteria | 58.56 | 0.63 | 1814849 | 314 | no_ssu |
| bin_1318 | Bacteria | Proteobacteria | Alphaproteobacteria | 89.78 | 2.16 | 2084186 | 164 | no_ssu |
| bin_1319 | Bacteria | Proteobacteria | Betaproteobacteria | 48.43 | 5.17 | 1773176 | 439 | no_ssu |
| bin_132 | Bacteria | Firmicutes | Clostridia | 3.33 | 2.65 | 618146 | 168 | no_ssu |
| bin_1320 | Bacteria | Bacteroidetes | Flavobacteriia | 0 | 0 | 369361 | 72 | no_ssu |
| bin_1321 | Bacteria | Actinobacteria | Actinobacteria | 91.45 | 3.16 | 3177961 | 230 | Uncultured bacterium clone BJGMM-3s-336 16S ribosomal RNA gene, partial sequence |
| bin_1322 | Bacteria | Proteobacteria | Alphaproteobacteria | 89.14 | 4.6 | 3589960 | 656 | Uncultured bacterium clone MC15 16S ribosomal RNA gene, partial sequence |
| bin_1323 | Bacteria | Proteobacteria | Gammaproteobacteria | 27.59 | 0.86 | 909644 | 297 | no_ssu |
| bin_1324 | Bacteria | Actinobacteria | Actinobacteria | 72.03 | 1.61 | 2568169 | 655 | no_ssu |
| bin_1325 | Bacteria | Proteobacteria | Gammaproteobacteria | 2.9 | 1.44 | 383458 | 33 | no_ssu |
| bin_1326 | Bacteria | Proteobacteria | Alphaproteobacteria | 33.92 | 0 | 760157 | 271 | no_ssu |
| bin_1327 | Bacteria | Bacteroidetes | Flavobacteriia | 76.24 | 5.9 | 2333061 | 459 | Uncultured bacterium clone 5C231472 16S ribosomal RNA gene, partial sequence |
| bin_1328 | Bacteria | Firmicutes | Clostridia | 2.11 | 0.07 | 418949 | 83 | no_ssu |
| bin_1329 | Bacteria | Proteobacteria | Gammaproteobacteria | 86.4 | 1.61 | 2521694 | 138 | no_ssu |
| bin_133 | Bacteria | Proteobacteria | Alphaproteobacteria | 74.9 | 3.14 | 3334748 | 705 | no_ssu |
| bin_1330 | Bacteria | Proteobacteria | Gammaproteobacteria | 78.9 | 7.91 | 1694911 | 383 | no_ssu |
| bin_1331 | Bacteria | Firmicutes | Bacilli | 2.66 | 0 | 290934 | 7 | no_ssu |
| bin_1332 | Bacteria | Proteobacteria | Gammaproteobacteria | 94.89 | 3.41 | 4368590 | 488 | Uncultured bacterium clone JN41 16S ribosomal RNA gene, partial sequence |
| bin_1333 | Viruses | NA | NA | 0 | 0 | 2725535 | 555 | no_ssu |
| bin_1334 | Bacteria | Bacteroidetes | Flavobacteriia | 0 | 0 | 308630 | 8 | no_ssu |
| bin_1335 | Bacteria | Firmicutes | Bacilli | 14.74 | 6.54 | 646925 | 114 | Uncultured bacterium clone 63-18 16S ribosomal RNA gene, partial sequence |
| bin_1336 | Bacteria | Proteobacteria | Gammaproteobacteria | 2.89 | 0.5 | 729194 | 229 | no_ssu |
| bin_1337 | Bacteria | Actinobacteria | Actinobacteria | 58.79 | 3.38 | 1003293 | 187 | no_ssu |
| bin_1338 | Bacteria | Bacteroidetes | Flavobacteriia | 77.59 | 30.39 | 1945873 | 529 | Flavobacteriaceae bacterium F202Z8 chromosome, complete genome |
| bin_1339 | Bacteria | Bacteroidetes | Flavobacteriia | 98.02 | 0 | 2753896 | 136 | no_ssu |
| bin_134 | Bacteria | Firmicutes | Bacilli | 1.37 | 0 | 584326 | 95 | Rhizaria sp. mitochondrion, complete genome |
| bin_1340 | Bacteria | Proteobacteria | Alphaproteobacteria | 45.41 | 6.98 | 2736830 | 864 | no_ssu |
| bin_1341 | Bacteria | Verrucomicrobia | Opitutae | 89.69 | 7.97 | 3042342 | 358 | no_ssu |
| bin_1342 | Bacteria | Actinobacteria | Actinobacteria | 65 | 2.56 | 1065766 | 281 | no_ssu |
| bin_1343 | Bacteria | Bacteroidetes | Flavobacteriia | 94.6 | 0.48 | 1830426 | 120 | Uncultured bacterium clone F3047 16S ribosomal RNA gene, partial sequence |
| bin_1344 | Bacteria | Proteobacteria | Gammaproteobacteria | 50.25 | 3.15 | 1361958 | 358 | no_ssu |
| bin_1345 | Bacteria | Proteobacteria | Gammaproteobacteria | 54.96 | 1.26 | 1198059 | 341 | no_ssu |
| bin_1346 | Bacteria | Bacteroidetes | Flavobacteriia | 79.15 | 0.51 | 2161488 | 442 | no_ssu |
| bin_1347 | Bacteria | Proteobacteria | Betaproteobacteria | 37.41 | 3.45 | 919653 | 263 | no_ssu |
| bin_1348 | Bacteria | Proteobacteria | Alphaproteobacteria | 29.96 | 0.78 | 1694379 | 586 | no_ssu |
| bin_1349 | Bacteria | Bacteroidetes | Flavobacteriia | 89.66 | 32.73 | 2601989 | 671 | no_ssu |
| bin_135 | Bacteria | Actinobacteria | Actinobacteria | 59.43 | 4.37 | 1270312 | 380 | no_ssu |
| bin_1350 | Bacteria | Nitrospirae | Nitrospira | 4.91 | 0.79 | 430261 | 26 | no_ssu |
| bin_1351 | Bacteria | Proteobacteria | Gammaproteobacteria | 44.81 | 1.15 | 2205465 | 687 | no_ssu |
| bin_1352 | Bacteria | Firmicutes | Clostridia | 5.76 | 7.9 | 1194389 | 361 | no_ssu |
| bin_1353 | Bacteria | Firmicutes | Bacilli | 62.81 | 3.37 | 609212 | 60 | Uncultured bacterium clone JC01 16S ribosomal RNA gene, partial sequence |
| bin_1354 | Bacteria | Proteobacteria | Betaproteobacteria | 15.52 | 0 | 370761 | 142 | no_ssu |
| bin_1355 | Bacteria | Proteobacteria | Betaproteobacteria | 60 | 0.86 | 1224634 | 114 | no_ssu |
| bin_1356 | Bacteria | Bacteroidetes | Flavobacteriia | 93.55 | 0.54 | 2557293 | 165 | Uncultured marine bacterium clone B1-42 16S ribosomal RNA gene, partial sequence |
| bin_1357 | Bacteria | Proteobacteria | Betaproteobacteria | 20.69 | 1.72 | 389345 | 130 | no_ssu |
| bin_1358 | Bacteria | Verrucomicrobia | Opitutae | 75.7 | 5.31 | 2692041 | 482 | no_ssu |
| bin_1359 | Bacteria | Actinobacteria | Actinobacteria | 76.2 | 3.51 | 1056907 | 76 | Uncultured bacterium clone 8 16S ribosomal RNA gene, partial sequence |
| bin_136 | Bacteria | Proteobacteria | Betaproteobacteria | 6.35 | 0 | 768794 | 251 | no_ssu |
| bin_1360 | Bacteria | Proteobacteria | Gammaproteobacteria | 78.47 | 5.05 | 2397381 | 420 | Rhodothermaceae bacterium strain MEBiC09517 chromosome, complete genome |
| bin_1361 | Bacteria | Firmicutes | Bacilli | 0 | 0 | 210539 | 44 | no_ssu |
| bin_1362 | Bacteria | Firmicutes | Clostridia | 6.33 | 1.08 | 330703 | 92 | no_ssu |
| bin_1363 | Bacteria | Proteobacteria | Alphaproteobacteria | 95.63 | 2.64 | 2356150 | 88 | no_ssu |
| bin_1364 | Bacteria | Proteobacteria | Gammaproteobacteria | 5.31 | 0.29 | 428043 | 99 | no_ssu |
| bin_1365 | Bacteria | Proteobacteria | Gammaproteobacteria | 4.17 | 0 | 258850 | 30 | no_ssu |
| bin_1366 | Bacteria | Proteobacteria | Gammaproteobacteria | 84.03 | 0.89 | 3658927 | 656 | Uncultured bacterium isolate 1112865261809a 16S ribosomal RNA gene, partial sequence |
| bin_1367 | Bacteria | Bacteroidetes | Flavobacteriia | 69.54 | 0.66 | 1752442 | 449 | Uncultured Sphingobacteriales bacterium clone Clip 105 16S ribosomal RNA gene, partial sequence |
| bin_1368 | Bacteria | Bacteroidetes | Cytophagia | 17.4 | 0 | 540183 | 171 | no_ssu |
| bin_1369 | Bacteria | Proteobacteria | Gammaproteobacteria | 94.08 | 0.56 | 6205501 | 298 | no_ssu |
| bin_137 | Bacteria | Bacteroidetes | Flavobacteriia | 61.31 | 2.42 | 1695914 | 504 | no_ssu |
| bin_1370 | Bacteria | Proteobacteria | Gammaproteobacteria | 69.94 | 4.93 | 1257866 | 165 | Bacterium enrichment culture clone 32(2013) 16S ribosomal RNA gene, partial sequence |
| bin_1371 | Bacteria | Proteobacteria | Gammaproteobacteria | 84.64 | 2.76 | 1305984 | 63 | Uncultured bacterium clone M1-40 16S ribosomal RNA gene, partial sequence |
| bin_1372 | Bacteria | Proteobacteria | Gammaproteobacteria | 0 | 0 | 286068 | 21 | no_ssu |
| bin_1373 | Bacteria | Proteobacteria | Alphaproteobacteria | 21.96 | 3.77 | 474752 | 111 | no_ssu |
| bin_1374 | Bacteria | Actinobacteria | Actinobacteria | 4.17 | 0 | 416160 | 90 | no_ssu |
| bin_1375 | Bacteria | Firmicutes | Bacilli | 3.83 | 1.44 | 583470 | 40 | no_ssu |
| bin_1376 | Bacteria | Proteobacteria | Gammaproteobacteria | 42.23 | 4.07 | 688155 | 186 | no_ssu |
| bin_1377 | Bacteria | Proteobacteria | Gammaproteobacteria | 64.95 | 5.69 | 2375022 | 690 | Psammoneis obaidii chloroplast, complete genome |
| bin_1378 | Bacteria | Actinobacteria | Actinobacteria | 39.32 | 0.85 | 1524931 | 506 | no_ssu |
| bin_1379 | Bacteria | Proteobacteria | Gammaproteobacteria | 85.53 | 3.4 | 4332308 | 643 | Uncultured bacterium clone OTU864 16S ribosomal RNA gene, partial sequence |
| bin_138 | Bacteria | Proteobacteria | Betaproteobacteria | 1.72 | 0 | 620321 | 161 | no_ssu |
| bin_1380 | Bacteria | Bacteroidetes | Flavobacteriia | 51.94 | 0.94 | 950558 | 226 | no_ssu |
| bin_1381 | Bacteria | Actinobacteria | Actinobacteria | 58.3 | 2.25 | 768367 | 165 | no_ssu |
| bin_1382 | Bacteria | Proteobacteria | Gammaproteobacteria | 51.13 | 2.41 | 1961004 | 431 | no_ssu |
| bin_1383 | Bacteria | Proteobacteria | Gammaproteobacteria | 15.52 | 1.72 | 496361 | 122 | no_ssu |
| bin_1384 | Bacteria | Proteobacteria | Alphaproteobacteria | 20.69 | 1.72 | 1140445 | 395 | no_ssu |
| bin_1385 | Bacteria | Firmicutes | Clostridia | 4.17 | 0 | 667352 | 173 | no_ssu |
| bin_1386 | Bacteria | Proteobacteria | Gammaproteobacteria | 81.82 | 3.61 | 3091061 | 467 | Uncultured bacterium clone LF231A36 16S ribosomal RNA gene, partial sequence |
| bin_1387 | Bacteria | Firmicutes | Bacilli | 84.33 | 0 | 784822 | 43 | Uncultured bacterium partial 16S rRNA gene, clone Sta4-05 |
| bin_1388 | Bacteria | Actinobacteria | Actinobacteria | 31.03 | 0 | 490257 | 135 | no_ssu |
| bin_1389 | Bacteria | Deinococcus-Thermus | Deinococci | 1.8 | 0 | 256486 | 11 | no_ssu |
| bin_139 | Bacteria | Proteobacteria | Alphaproteobacteria | 49.42 | 11.21 | 1635325 | 511 | no_ssu |
| bin_1390 | Bacteria | Actinobacteria | Actinobacteria | 60.28 | 20.32 | 1130220 | 313 | Candidatus Rhodoluna planktonica strain MWH-Dar1, complete genome |
| bin_1391 | Bacteria | Firmicutes | Clostridia | 4.3 | 0 | 500219 | 136 | no_ssu |
| bin_1392 | Bacteria | Proteobacteria | Gammaproteobacteria | 20.49 | 0 | 599242 | 212 | no_ssu |
| bin_1393 | Bacteria | Bacteroidetes | Flavobacteriia | 17.59 | 0 | 945717 | 331 | no_ssu |
| bin_1394 | Bacteria | Proteobacteria | Betaproteobacteria | 16.67 | 0 | 835432 | 208 | no_ssu |
| bin_1395 | Bacteria | Proteobacteria | Alphaproteobacteria | 96.68 | 3.64 | 3077063 | 238 | Uncultured bacterium clone E7-150bp 16S ribosomal RNA gene, partial sequence |
| bin_14 | Bacteria | Proteobacteria | Alphaproteobacteria | 4.17 | 0 | 727476 | 252 | no_ssu |
| bin_140 | Bacteria | Proteobacteria | Gammaproteobacteria | 3.45 | 0.29 | 611821 | 99 | no_ssu |
| bin_141 | Bacteria | Bacteroidetes | Flavobacteriia | 84.11 | 7.12 | 1860573 | 341 | no_ssu |
| bin_142 | Bacteria | Actinobacteria | Actinobacteria | 3.04 | 0.5 | 429190 | 10 | no_ssu |
| bin_143 | Bacteria | Actinobacteria | Actinobacteria | 28.79 | 1.71 | 891243 | 264 | Uncultured actinobacterium HF0070_17F14 genomic sequence |
| bin_144 | Bacteria | Bacteroidetes | Flavobacteriia | 3.91 | 0.93 | 739962 | 94 | no_ssu |
| bin_145 | Bacteria | Actinobacteria | Actinobacteria | 58.78 | 31.03 | 1275863 | 273 | no_ssu |
| bin_146 | Bacteria | Proteobacteria | Alphaproteobacteria | 90.88 | 14.9 | 1561817 | 225 | Uncultured alpha proteobacterium clone S1-3-16 16S ribosomal RNA gene, partial sequence |
| bin_147 | Bacteria | Proteobacteria | Gammaproteobacteria | 39.66 | 6.9 | 929097 | 242 | no_ssu |
| bin_148 | Bacteria | Actinobacteria | Actinobacteria | 12.65 | 0.9 | 3774228 | 1395 | Spirostomum sp. small subunit ribosomal RNA gene, partial sequence |
| bin_149 | Bacteria | Bacteroidetes | Flavobacteriia | 88.64 | 15.75 | 2248834 | 381 | Uncultured Flavobacteria bacterium 16S rRNA gene, clone NorSea47 |
| bin_15 | Bacteria | Actinobacteria | Actinobacteria | 55.26 | 1.75 | 684858 | 195 | no_ssu |
| bin_150 | Bacteria | Proteobacteria | Alphaproteobacteria | 5.09 | 1.89 | 201148 | 67 | no_ssu |
| bin_151 | Bacteria | Actinobacteria | Actinobacteria | 0 | 0 | 355223 | 27 | no_ssu |
| bin_152 | Bacteria | Bacteroidetes | Flavobacteriia | 75.21 | 1.93 | 1544577 | 202 | no_ssu |
| bin_153 | Bacteria | Bacteroidetes | Flavobacteriia | 98.28 | 7.05 | 2324624 | 329 | no_ssu |
| bin_154 | Bacteria | Proteobacteria | Gammaproteobacteria | 3.51 | 17.54 | 1050033 | 241 | no_ssu |
| bin_155 | Bacteria | Proteobacteria | Gammaproteobacteria | 83.23 | 16.06 | 10205713 | 2549 | no_ssu |
| bin_156 | Bacteria | Bacteroidetes | Flavobacteriia | 81.61 | 4.05 | 1816870 | 373 | no_ssu |
| bin_157 | Bacteria | Firmicutes | Bacilli | 0 | 0 | 204601 | 26 | no_ssu |
| bin_158 | Bacteria | Proteobacteria | Gammaproteobacteria | 8.58 | 0 | 519688 | 181 | no_ssu |
| bin_159 | Bacteria | Proteobacteria | Gammaproteobacteria | 24.61 | 1.11 | 414637 | 128 | no_ssu |
| bin_16 | Bacteria | Firmicutes | Clostridia | 1.44 | 0.36 | 576987 | 198 | no_ssu |
| bin_160 | Bacteria | Proteobacteria | Betaproteobacteria | 90.66 | 3.9 | 3309212 | 536 | Hydrogenophaga sp. PAMC20947 chromosome, complete genome |
| bin_161 | Bacteria | Firmicutes | Bacilli | 2.61 | 0.14 | 281714 | 71 | no_ssu |
| bin_162 | Bacteria | Proteobacteria | Betaproteobacteria | 56.25 | 10.92 | 1204952 | 336 | no_ssu |
| bin_163 | Bacteria | Actinobacteria | Actinobacteria | 17.4 | 0 | 425519 | 135 | no_ssu |
| bin_164 | Bacteria | Proteobacteria | Gammaproteobacteria | 0 | 0 | 310727 | 86 | no_ssu |
| bin_165 | Bacteria | Proteobacteria | Alphaproteobacteria | 91.38 | 68.76 | 1867296 | 307 | Uncultured bacterium clone PN07-5-54 16S ribosomal RNA gene, partial sequence |
| bin_166 | Bacteria | Bacteroidetes | Cytophagia | 24.14 | 1.72 | 1270592 | 419 | no_ssu |
| bin_167 | Bacteria | Firmicutes | Clostridia | 29.53 | 26.87 | 8799335 | 1540 | Uncultured alpha proteobacterium clone SanDiego8-G1 16S ribosomal RNA gene, partial sequence |
| bin_168 | Bacteria | Proteobacteria | Alphaproteobacteria | 90.29 | 53.91 | 6635910 | 1228 | no_ssu |
| bin_169 | Bacteria | Proteobacteria | Alphaproteobacteria | 66.7 | 4.02 | 3105717 | 642 | Uncultured bacterium clone W1-55 16S ribosomal RNA gene, partial sequence |
| bin_17 | Viruses | NA | NA | 0 | 0 | 219523 | 7 | no_ssu |
| bin_170 | Bacteria | Proteobacteria | Gammaproteobacteria | 50.48 | 3.75 | 981490 | 265 | Uncultured gamma proteobacterium EBAC_27G05 genomic sequence |
| bin_171 | Bacteria | Proteobacteria | Gammaproteobacteria | 90.26 | 2.23 | 3170544 | 210 | Uncultured bacterium clone NN44 16S ribosomal RNA gene, partial sequence |
| bin_172 | Bacteria | Actinobacteria | Actinobacteria | 34.45 | 0 | 523537 | 149 | no_ssu |
| bin_173 | Bacteria | Proteobacteria | Gammaproteobacteria | 34.01 | 0.16 | 1133208 | 333 | no_ssu |
| bin_174 | Bacteria | Bacteroidetes | Flavobacteriia | 54.66 | 27.59 | 2613050 | 833 | no_ssu |
| bin_175 | Bacteria | Proteobacteria | Alphaproteobacteria | 17.55 | 3.45 | 833830 | 302 | no_ssu |
| bin_176 | Bacteria | NA | NA | 0 | 0 | 252006 | 45 | no_ssu |
| bin_177 | Bacteria | Bacteroidetes | Flavobacteriia | 93.41 | 1.26 | 2759816 | 214 | no_ssu |
| bin_178 | Bacteria | Firmicutes | Bacilli | 4.13 | 0.5 | 400541 | 37 | no_ssu |
| bin_179 | Bacteria | Proteobacteria | Betaproteobacteria | 22.68 | 0.12 | 594528 | 226 | no_ssu |
| bin_18 | Bacteria | Proteobacteria | Alphaproteobacteria | 36.16 | 0 | 1122068 | 313 | no_ssu |
| bin_180 | Bacteria | Proteobacteria | Gammaproteobacteria | 84.06 | 0.84 | 3328212 | 559 | no_ssu |
| bin_181 | Bacteria | Firmicutes | Bacilli | 4.91 | 0.79 | 526898 | 35 | no_ssu |
| bin_182 | Bacteria | Firmicutes | Bacilli | 0 | 0 | 214359 | 42 | no_ssu |
| bin_183 | Bacteria | Proteobacteria | Alphaproteobacteria | 13.01 | 0 | 907705 | 296 | no_ssu |
| bin_184 | Bacteria | Bacteroidetes | Flavobacteriia | 4.13 | 0 | 372769 | 17 | no_ssu |
| bin_185 | Bacteria | Proteobacteria | Alphaproteobacteria | 19.61 | 3.57 | 1747456 | 551 | no_ssu |
| bin_186 | Bacteria | Actinobacteria | Actinobacteria | 65.15 | 8.21 | 1740288 | 463 | no_ssu |
| bin_187 | Bacteria | Firmicutes | Bacilli | 0 | 0 | 264798 | 19 | no_ssu |
| bin_188 | Bacteria | Proteobacteria | Gammaproteobacteria | 96.49 | 9.25 | 7824658 | 1225 | no_ssu |
| bin_189 | Bacteria | Proteobacteria | Betaproteobacteria | 13.32 | 0 | 615645 | 227 | no_ssu |
| bin_19 | Bacteria | Proteobacteria | Gammaproteobacteria | 5.38 | 0.5 | 426680 | 25 | no_ssu |
| bin_190 | Bacteria | Proteobacteria | Alphaproteobacteria | 68.85 | 2.39 | 2551216 | 630 | no_ssu |
| bin_191 | Bacteria | Proteobacteria | Gammaproteobacteria | 38.03 | 2.59 | 1154197 | 354 | no_ssu |
| bin_192 | Bacteria | Proteobacteria | Gammaproteobacteria | 76.76 | 5.42 | 2143862 | 429 | no_ssu |
| bin_193 | Bacteria | Proteobacteria | Gammaproteobacteria | 3.2 | 0 | 308216 | 23 | no_ssu |
| bin_194 | Bacteria | Bacteroidetes | Flavobacteriia | 20.38 | 0 | 799934 | 258 | no_ssu |
| bin_195 | Bacteria | Firmicutes | Bacilli | 0 | 0 | 243338 | 74 | Thraustochytrium aureum mitochondrial DNA, partial genome |
| bin_196 | Bacteria | Proteobacteria | Gammaproteobacteria | 3.2 | 0.5 | 330451 | 54 | no_ssu |
| bin_197 | Bacteria | Bacteroidetes | Flavobacteriia | 20 | 0 | 521349 | 159 | no_ssu |
| bin_198 | Bacteria | Firmicutes | Bacilli | 75.23 | 0 | 739647 | 72 | no_ssu |
| bin_199 | Bacteria | Proteobacteria | Alphaproteobacteria | 90.26 | 2.71 | 2118009 | 402 | no_ssu |
| bin_2 | Bacteria | Proteobacteria | Gammaproteobacteria | 91.38 | 27.07 | 3759889 | 337 | Thiohalobacter thiocyanaticus DNA, complete genome, strain: FOKN1 |
| bin_20 | Bacteria | Proteobacteria | Betaproteobacteria | 92.18 | 9.2 | 2113384 | 326 | no_ssu |
| bin_200 | Bacteria | Bacteroidetes | Flavobacteriia | 3.04 | 0 | 302076 | 19 | no_ssu |
| bin_201 | Bacteria | Firmicutes | Bacilli | 7.66 | 0 | 693560 | 43 | no_ssu |
| bin_202 | Bacteria | Proteobacteria | Alphaproteobacteria | 91.18 | 3.33 | 3249152 | 534 | Uncultured alpha proteobacterium HF0010_30A23 genomic sequence |
| bin_203 | Bacteria | Proteobacteria | Alphaproteobacteria | 81.12 | 54.23 | 5700470 | 1521 | no_ssu |
| bin_204 | Bacteria | Firmicutes | Bacilli | 4.06 | 0.5 | 423133 | 87 | no_ssu |
| bin_205 | Bacteria | Proteobacteria | Gammaproteobacteria | 53.71 | 3.76 | 1546962 | 422 | no_ssu |
| bin_206 | Bacteria | Proteobacteria | Alphaproteobacteria | 80.26 | 21.15 | 3488576 | 967 | no_ssu |
| bin_207 | Bacteria | Proteobacteria | Gammaproteobacteria | 94.27 | 6.67 | 2946640 | 246 | no_ssu |
| bin_208 | Bacteria | Proteobacteria | Gammaproteobacteria | 97.85 | 5.38 | 4587778 | 326 | Uncultured bacterium clone SHAB590 16S ribosomal RNA gene, partial sequence |
| bin_209 | Bacteria | Tenericutes | Mollicutes | 0 | 0 | 283057 | 26 | no_ssu |
| bin_21 | Bacteria | Proteobacteria | Betaproteobacteria | 83.15 | 35.57 | 4172181 | 1071 | no_ssu |
| bin_210 | Bacteria | Firmicutes | Bacilli | 3.15 | 0 | 300946 | 71 | no_ssu |
| bin_211 | Bacteria | Proteobacteria | Alphaproteobacteria | 88.46 | 7.34 | 3267620 | 367 | Uncultured bacterium clone PS-12G02 16S ribosomal RNA gene, partial sequence |
| bin_212 | Bacteria | Firmicutes | Bacilli | 8.34 | 1.62 | 1079356 | 231 | no_ssu |
| bin_213 | Bacteria | Proteobacteria | Gammaproteobacteria | 36.28 | 1.79 | 1344226 | 417 | no_ssu |
| bin_214 | Bacteria | Proteobacteria | Gammaproteobacteria | 14.66 | 3.45 | 550793 | 195 | no_ssu |
| bin_215 | Bacteria | Bacteroidetes | Flavobacteriia | 90.98 | 2.71 | 2066220 | 238 | no_ssu |
| bin_216 | Bacteria | Bacteroidetes | Flavobacteriia | 0 | 0 | 319398 | 62 | no_ssu |
| bin_217 | Bacteria | Proteobacteria | Gammaproteobacteria | 0 | 0 | 410220 | 88 | no_ssu |
| bin_218 | Bacteria | Bacteroidetes | Flavobacteriia | 15.69 | 0 | 1039311 | 359 | no_ssu |
| bin_219 | Bacteria | Actinobacteria | Actinobacteria | 41.51 | 2.43 | 564975 | 157 | Uncultured bacterium clone 5C231482 16S ribosomal RNA gene, partial sequence |
| bin_22 | Bacteria | Proteobacteria | Alphaproteobacteria | 64.04 | 17.93 | 3414377 | 915 | no_ssu |
| bin_220 | Bacteria | Proteobacteria | Gammaproteobacteria | 50.93 | 1.69 | 2096394 | 640 | no_ssu |
| bin_221 | Bacteria | Firmicutes | Bacilli | 38.18 | 1.82 | 548291 | 173 | no_ssu |
| bin_222 | Bacteria | Proteobacteria | Betaproteobacteria | 37.43 | 8.46 | 1952580 | 616 | no_ssu |
| bin_223 | Bacteria | Bacteroidetes | Flavobacteriia | 83.61 | 5.65 | 2096157 | 223 | no_ssu |
| bin_224 | Bacteria | Firmicutes | Bacilli | 64.18 | 15.87 | 1032971 | 175 | no_ssu |
| bin_225 | Archaea | Thaumarchaeota | NA | 72.01 | 1.46 | 1065818 | 180 | no_ssu |
| bin_226 | Bacteria | Bacteroidetes | Cytophagia | 4.17 | 0 | 285573 | 102 | no_ssu |
| bin_227 | Bacteria | Proteobacteria | Gammaproteobacteria | 31.9 | 0 | 589681 | 194 | no_ssu |
| bin_228 | Bacteria | Bacteroidetes | Flavobacteriia | 4.17 | 0 | 372135 | 80 | no_ssu |
| bin_229 | Bacteria | Proteobacteria | Gammaproteobacteria | 33.56 | 0.89 | 982371 | 318 | no_ssu |
| bin_23 | Bacteria | Firmicutes | Bacilli | 48.75 | 0.57 | 833682 | 206 | no_ssu |
| bin_230 | Bacteria | Proteobacteria | Gammaproteobacteria | 50.05 | 18.99 | 20431296 | 6171 | no_ssu |
| bin_231 | Bacteria | Proteobacteria | Alphaproteobacteria | 81.76 | 10.19 | 2277481 | 498 | no_ssu |
| bin_232 | Bacteria | Proteobacteria | Gammaproteobacteria | 0 | 0 | 227402 | 45 | no_ssu |
| bin_233 | Bacteria | Proteobacteria | Gammaproteobacteria | 65.86 | 10.34 | 2047119 | 550 | no_ssu |
| bin_234 | Bacteria | Firmicutes | Erysipelotrichia | 4.39 | 0 | 390522 | 62 | no_ssu |
| bin_235 | Bacteria | Firmicutes | Bacilli | 84.33 | 1.72 | 706153 | 67 | no_ssu |
| bin_236 | Bacteria | Proteobacteria | Alphaproteobacteria | 4.17 | 0 | 644341 | 103 | no_ssu |
| bin_237 | Bacteria | Actinobacteria | Actinobacteria | 55.25 | 4.56 | 980808 | 251 | no_ssu |
| bin_238 | Bacteria | Proteobacteria | Betaproteobacteria | 20.69 | 1.72 | 985955 | 383 | no_ssu |
| bin_239 | Bacteria | Proteobacteria | Alphaproteobacteria | 56.71 | 2.38 | 2092866 | 672 | no_ssu |
| bin_24 | Bacteria | Proteobacteria | Alphaproteobacteria | 96.39 | 55.75 | 4429082 | 676 | no_ssu |
| bin_240 | Bacteria | Actinobacteria | Actinobacteria | 55.93 | 1.69 | 663593 | 151 | no_ssu |
| bin_241 | Bacteria | Proteobacteria | Gammaproteobacteria | 28.14 | 0 | 1219425 | 414 | Uncultured bacterium clone MCW3J6 16S ribosomal RNA gene, partial sequence |
| bin_242 | Bacteria | Actinobacteria | Actinobacteria | 65.87 | 1.71 | 967111 | 181 | no_ssu |
| bin_243 | Bacteria | Cyanobacteria | NA | 2.73 | 0.5 | 448593 | 33 | no_ssu |
| bin_244 | Bacteria | Proteobacteria | Oligoflexia | 74.18 | 3.68 | 2407419 | 551 | no_ssu |
| bin_245 | Bacteria | Proteobacteria | Gammaproteobacteria | 0 | 0 | 338775 | 95 | no_ssu |
| bin_246 | Bacteria | Proteobacteria | Alphaproteobacteria | 67.44 | 2.59 | 985333 | 237 | no_ssu |
| bin_247 | Bacteria | Proteobacteria | Alphaproteobacteria | 28.31 | 3.28 | 1158362 | 376 | no_ssu |
| bin_248 | Bacteria | Actinobacteria | Actinobacteria | 26.02 | 0 | 384101 | 100 | no_ssu |
| bin_249 | Bacteria | Proteobacteria | Gammaproteobacteria | 24.12 | 0.86 | 408321 | 152 | no_ssu |
| bin_25 | Bacteria | Proteobacteria | Gammaproteobacteria | 75.05 | 6.59 | 2307421 | 435 | no_ssu |
| bin_250 | Viruses | NA | NA | 0 | 0 | 305351 | 80 | no_ssu |
| bin_251 | Bacteria | Proteobacteria | Alphaproteobacteria | 54.76 | 12.51 | 2339210 | 690 | no_ssu |
| bin_252 | Viruses | NA | NA | 0 | 0 | 1348142 | 352 | no_ssu |
| bin_253 | Bacteria | Bacteroidetes | Flavobacteriia | 92.49 | 2.43 | 2014409 | 239 | no_ssu |
| bin_254 | Bacteria | Actinobacteria | Actinobacteria | 9.48 | 0 | 587190 | 46 | no_ssu |
| bin_255 | Bacteria | Proteobacteria | Gammaproteobacteria | 0 | 0 | 450390 | 108 | no_ssu |
| bin_256 | Bacteria | Proteobacteria | Gammaproteobacteria | 73.01 | 2.11 | 1625435 | 326 | no_ssu |
| bin_257 | Bacteria | Cyanobacteria | NA | 5.17 | 0 | 289364 | 83 | no_ssu |
| bin_258 | Bacteria | Proteobacteria | Gammaproteobacteria | 37.93 | 0 | 1775652 | 453 | no_ssu |
| bin_259 | Bacteria | Proteobacteria | Gammaproteobacteria | 0 | 0 | 232946 | 40 | no_ssu |
| bin_26 | Bacteria | Proteobacteria | Betaproteobacteria | 55.5 | 10.45 | 1084927 | 339 | Methylophilaceae bacterium NB0092 16S ribosomal RNA gene, partial sequence |
| bin_260 | Bacteria | Proteobacteria | Gammaproteobacteria | 4.8 | 0 | 416075 | 137 | no_ssu |
| bin_261 | Bacteria | Proteobacteria | Alphaproteobacteria | 14.71 | 4.62 | 11787295 | 3947 | no_ssu |
| bin_262 | Bacteria | Bacteroidetes | Flavobacteriia | 92.31 | 26.34 | 4055189 | 843 | Actinobacteria bacterium IMCC26256, complete genome |
| bin_263 | Bacteria | Bacteroidetes | Flavobacteriia | 67.93 | 6.92 | 1244730 | 303 | Uncultured bacterium clone W2-17 16S ribosomal RNA gene, partial sequence |
| bin_264 | Bacteria | Proteobacteria | Betaproteobacteria | 4.31 | 0 | 322493 | 130 | no_ssu |
| bin_265 | Bacteria | Proteobacteria | Gammaproteobacteria | 91.4 | 2.33 | 2004120 | 36 | Uncultured bacterium clone eff98 16S ribosomal RNA gene, partial sequence |
| bin_266 | Bacteria | Proteobacteria | Gammaproteobacteria | 0 | 0 | 241240 | 42 | no_ssu |
| bin_267 | Bacteria | Proteobacteria | Alphaproteobacteria | 5.8 | 10.34 | 504293 | 138 | no_ssu |
| bin_268 | Bacteria | Bacteroidetes | Bacteroidia | 0 | 0 | 332336 | 74 | no_ssu |
| bin_269 | Bacteria | Proteobacteria | Gammaproteobacteria | 3.92 | 0 | 555577 | 170 | no_ssu |
| bin_27 | Bacteria | Bacteroidetes | Flavobacteriia | 4.17 | 0 | 392981 | 115 | no_ssu |
| bin_270 | Bacteria | Proteobacteria | Betaproteobacteria | 62.79 | 2.69 | 1196258 | 255 | no_ssu |
| bin_271 | Viruses | NA | NA | 0 | 0 | 359105 | 65 | no_ssu |
| bin_272 | Bacteria | Cyanobacteria | NA | 47.93 | 8.62 | 1387235 | 466 | no_ssu |
| bin_273 | Bacteria | Proteobacteria | Betaproteobacteria | 67.76 | 7.76 | 1443840 | 191 | Uncultured bacterium clone E153 16S ribosomal RNA gene, partial sequence |
| bin_274 | Bacteria | Proteobacteria | Gammaproteobacteria | 59 | 3.57 | 1625033 | 442 | no_ssu |
| bin_275 | Bacteria | Proteobacteria | Gammaproteobacteria | 68.07 | 2.98 | 2366138 | 542 | no_ssu |
| bin_276 | Bacteria | Proteobacteria | Gammaproteobacteria | 5.9 | 0.07 | 1326031 | 499 | no_ssu |
| bin_277 | Bacteria | Proteobacteria | Betaproteobacteria | 83.26 | 4.85 | 1720328 | 239 | no_ssu |
| bin_278 | Bacteria | Proteobacteria | Alphaproteobacteria | 41.88 | 1.88 | 1386959 | 432 | no_ssu |
| bin_279 | Bacteria | Bacteroidetes | Cytophagia | 4.17 | 0 | 285915 | 33 | no_ssu |
| bin_28 | Bacteria | Actinobacteria | Actinobacteria | 74.36 | 1.75 | 2014851 | 378 | no_ssu |
| bin_280 | Bacteria | Bacteroidetes | Flavobacteriia | 77.27 | 3.07 | 1999885 | 441 | Uncultured delta proteobacterium clone I50-0320 16S ribosomal RNA gene, partial sequence |
| bin_281 | Bacteria | Cyanobacteria | NA | 26.03 | 0 | 392330 | 133 | no_ssu |
| bin_282 | Bacteria | Actinobacteria | Actinobacteria | 90.99 | 6.38 | 2933831 | 477 | no_ssu |
| bin_283 | Bacteria | Firmicutes | Bacilli | 0 | 0 | 386566 | 83 | no_ssu |
| bin_284 | Bacteria | Firmicutes | Bacilli | 2.79 | 0.22 | 405166 | 90 | no_ssu |
| bin_285 | Bacteria | Firmicutes | Bacilli | 3.55 | 0 | 338340 | 55 | no_ssu |
| bin_286 | Bacteria | Bacteroidetes | Flavobacteriia | 0 | 0 | 380819 | 30 | no_ssu |
| bin_287 | Bacteria | Firmicutes | Bacilli | 0 | 0 | 222048 | 44 | no_ssu |
| bin_288 | Bacteria | Bacteroidetes | Flavobacteriia | 81.51 | 1.84 | 1599718 | 240 | no_ssu |
| bin_289 | Bacteria | Firmicutes | Bacilli | 4.36 | 7.86 | 1932371 | 459 | no_ssu |
| bin_29 | Bacteria | Proteobacteria | Gammaproteobacteria | 6.46 | 0.58 | 849569 | 334 | no_ssu |
| bin_290 | Bacteria | Proteobacteria | Alphaproteobacteria | 0 | 0 | 349369 | 15 | no_ssu |
| bin_291 | Bacteria | Proteobacteria | Alphaproteobacteria | 1.72 | 0.87 | 442908 | 114 | no_ssu |
| bin_292 | Bacteria | Bacteroidetes | Flavobacteriia | 73.01 | 4.45 | 2046487 | 497 | Uncultured bacterium clone D8W_97 16S ribosomal RNA gene, partial sequence |
| bin_293 | Bacteria | Proteobacteria | Alphaproteobacteria | 61.5 | 39.36 | 4841316 | 1538 | no_ssu |
| bin_294 | Bacteria | Proteobacteria | Gammaproteobacteria | 3.44 | 0.14 | 347834 | 83 | no_ssu |
| bin_295 | Bacteria | Cyanobacteria | NA | 5.17 | 0 | 281514 | 97 | no_ssu |
| bin_296 | Bacteria | Proteobacteria | Betaproteobacteria | 22.41 | 3.45 | 1731370 | 606 | no_ssu |
| bin_297 | Bacteria | Proteobacteria | Betaproteobacteria | 15.34 | 0 | 598165 | 213 | no_ssu |
| bin_298 | Bacteria | Firmicutes | Bacilli | 3.04 | 0.5 | 504816 | 79 | no_ssu |
| bin_299 | Bacteria | Proteobacteria | Alphaproteobacteria | 80.76 | 2.38 | 4173065 | 957 | no_ssu |
| bin_3 | Bacteria | Cyanobacteria | NA | 75.98 | 1.43 | 3840341 | 908 | no_ssu |
| bin_30 | Bacteria | Firmicutes | Clostridia | 6.31 | 2.84 | 373159 | 86 | no_ssu |
| bin_300 | Bacteria | Bacteroidetes | Flavobacteriia | 77.7 | 60.69 | 3046242 | 505 | no_ssu |
| bin_301 | Bacteria | Actinobacteria | Actinobacteria | 0 | 0 | 236202 | 22 | no_ssu |
| bin_302 | Bacteria | Proteobacteria | Betaproteobacteria | 25.96 | 57.89 | 1804217 | 468 | no_ssu |
| bin_303 | Bacteria | Proteobacteria | Gammaproteobacteria | 0 | 0 | 578549 | 163 | no_ssu |
| bin_304 | Bacteria | Bacteroidetes | Flavobacteriia | 2.68 | 0 | 506386 | 106 | no_ssu |
| bin_305 | Bacteria | Proteobacteria | Betaproteobacteria | 10.34 | 0 | 982185 | 348 | no_ssu |
| bin_306 | Bacteria | Proteobacteria | Alphaproteobacteria | 55.52 | 3.45 | 2009252 | 506 | no_ssu |
| bin_307 | Bacteria | Proteobacteria | Gammaproteobacteria | 77.59 | 6.03 | 3620837 | 534 | no_ssu |
| bin_308 | Bacteria | Proteobacteria | Alphaproteobacteria | 0 | 0 | 220702 | 33 | no_ssu |
| bin_309 | Bacteria | Proteobacteria | Alphaproteobacteria | 94.37 | 6.94 | 4941496 | 813 | no_ssu |
| bin_31 | Bacteria | Actinobacteria | Actinobacteria | 86.99 | 2.56 | 1895045 | 255 | Uncultured marine bacterium clone S17-78 16S ribosomal RNA gene, partial sequence |
| bin_310 | Bacteria | Actinobacteria | Actinobacteria | 63.34 | 87.38 | 1722151 | 518 | no_ssu |
| bin_311 | Bacteria | Proteobacteria | Betaproteobacteria | 57.55 | 2.59 | 2311501 | 437 | no_ssu |
| bin_312 | Bacteria | Proteobacteria | Betaproteobacteria | 0 | 0 | 375536 | 55 | no_ssu |
| bin_313 | Bacteria | Firmicutes | Bacilli | 8.65 | 1.29 | 767158 | 133 | no_ssu |
| bin_314 | Bacteria | Bacteroidetes | Flavobacteriia | 98.92 | 0 | 2441059 | 150 | Uncultured Cryomorphaceae bacterium partial 16S rRNA gene, clone ZS-1-345 |
| bin_315 | Bacteria | Proteobacteria | Gammaproteobacteria | 60.37 | 0.03 | 1493755 | 405 | no_ssu |
| bin_316 | Bacteria | Proteobacteria | Gammaproteobacteria | 3.04 | 0 | 356768 | 25 | no_ssu |
| bin_317 | Bacteria | Bacteroidetes | Flavobacteriia | 78.47 | 5.15 | 1281597 | 156 | Uncultured marine bacterium clone 155D1Bb04Q 16S ribosomal RNA gene, partial sequence |
| bin_318 | Bacteria | Proteobacteria | Gammaproteobacteria | 0 | 0 | 357297 | 12 | no_ssu |
| bin_319 | Bacteria | Proteobacteria | Alphaproteobacteria | 40.26 | 3.03 | 643733 | 114 | no_ssu |
| bin_32 | Bacteria | Cyanobacteria | NA | 0 | 0 | 313080 | 77 | no_ssu |
| bin_320 | Bacteria | Proteobacteria | Gammaproteobacteria | 27.59 | 1.72 | 1847433 | 551 | no_ssu |
| bin_321 | Bacteria | Firmicutes | Bacilli | 1.01 | 0 | 360449 | 87 | no_ssu |
| bin_322 | Bacteria | Proteobacteria | Gammaproteobacteria | 29.08 | 5.2 | 2191362 | 740 | Uncultured Auriculariaceae clone Elev_18S_1278 18S ribosomal RNA gene, partial sequence |
| bin_323 | Bacteria | Actinobacteria | Actinobacteria | 97.44 | 7.48 | 2379587 | 164 | Uncultured marine bacterium clone S17-152 16S ribosomal RNA gene, partial sequence |
| bin_324 | Bacteria | Proteobacteria | Gammaproteobacteria | 4.13 | 0.43 | 330051 | 61 | no_ssu |
| bin_325 | Bacteria | Proteobacteria | Alphaproteobacteria | 37.93 | 6.9 | 1401268 | 535 | no_ssu |
| bin_326 | Bacteria | Proteobacteria | Gammaproteobacteria | 81.75 | 1.75 | 2758657 | 158 | Uncultured bacterium clone S73_054 16S ribosomal RNA gene, partial sequence |
| bin_327 | Bacteria | Proteobacteria | Alphaproteobacteria | 55.97 | 15.06 | 664916 | 122 | no_ssu |
| bin_328 | Bacteria | Proteobacteria | Gammaproteobacteria | 1.72 | 0 | 303780 | 65 | no_ssu |
| bin_329 | Bacteria | Firmicutes | Bacilli | 4.91 | 0.5 | 538099 | 50 | no_ssu |
| bin_33 | Bacteria | Bacteroidetes | Flavobacteriia | 68.36 | 6.36 | 1009624 | 189 | Uncultured marine bacterium clone S10-15 16S ribosomal RNA gene, partial sequence |
| bin_330 | Bacteria | Cyanobacteria | NA | 5.54 | 2.95 | 650598 | 166 | no_ssu |
| bin_331 | Bacteria | Proteobacteria | Betaproteobacteria | 76.98 | 3.79 | 1913312 | 423 | Uncultured bacterium clone T2S203A04 16S ribosomal RNA gene, partial sequence |
| bin_332 | Bacteria | Firmicutes | Bacilli | 0 | 0 | 248209 | 26 | no_ssu |
| bin_333 | Bacteria | Bacteroidetes | Bacteroidia | 4.92 | 0 | 348268 | 71 | no_ssu |
| bin_334 | Bacteria | Proteobacteria | Gammaproteobacteria | 0 | 0 | 283211 | 87 | no_ssu |
| bin_335 | Bacteria | Proteobacteria | Gammaproteobacteria | 47.3 | 0.89 | 2266880 | 673 | no_ssu |
| bin_336 | Bacteria | Actinobacteria | Actinobacteria | 88.73 | 12.86 | 3065656 | 392 | Humibacter sp. WJ7-1 chromosome, complete genome |
| bin_337 | Bacteria | Cyanobacteria | NA | 29.48 | 6.9 | 1051191 | 301 | no_ssu |
| bin_338 | Bacteria | Actinobacteria | Actinobacteria | 32.92 | 0 | 542606 | 156 | no_ssu |
| bin_339 | Bacteria | Firmicutes | Bacilli | 3.98 | 0.5 | 504557 | 44 | no_ssu |
| bin_34 | Bacteria | Proteobacteria | Alphaproteobacteria | 94.23 | 2.6 | 2857317 | 316 | no_ssu |
| bin_340 | Bacteria | Proteobacteria | Alphaproteobacteria | 2.76 | 0 | 313190 | 46 | no_ssu |
| bin_341 | Bacteria | Proteobacteria | Betaproteobacteria | 91.43 | 134.28 | 6230551 | 1691 | no_ssu |
| bin_342 | Bacteria | Proteobacteria | Betaproteobacteria | 92.32 | 1.12 | 2824490 | 341 | Sutterella sp. strain ASD3426 16S ribosomal RNA gene, partial sequence |
| bin_343 | Bacteria | Proteobacteria | Gammaproteobacteria | 80.57 | 6.45 | 6943019 | 1325 | Uncultured bacterium clone 8S24 16S ribosomal RNA gene, partial sequence |
| bin_344 | Bacteria | Proteobacteria | Oligoflexia | 0 | 0 | 347575 | 71 | no_ssu |
| bin_345 | Bacteria | Firmicutes | Bacilli | 4.39 | 0 | 730949 | 225 | no_ssu |
| bin_346 | Bacteria | Proteobacteria | Gammaproteobacteria | 0 | 0 | 206707 | 10 | no_ssu |
| bin_347 | Bacteria | Cyanobacteria | NA | 24.22 | 1.72 | 1238631 | 366 | no_ssu |
| bin_348 | Bacteria | Fusobacteria | Fusobacteriia | 3.67 | 0 | 201571 | 25 | no_ssu |
| bin_349 | Bacteria | Proteobacteria | Gammaproteobacteria | 0.86 | 0 | 322692 | 7 | no_ssu |
| bin_35 | Bacteria | Proteobacteria | Gammaproteobacteria | 28.1 | 11.66 | 3905308 | 1141 | no_ssu |
| bin_350 | Bacteria | Proteobacteria | Gammaproteobacteria | 86.1 | 1.12 | 5854131 | 790 | Uncultured planctomycete clone PN06-10-79 16S ribosomal RNA gene, partial sequence |
| bin_351 | Bacteria | Proteobacteria | Alphaproteobacteria | 22.64 | 45.28 | 802056 | 203 | Chroomonas mesostigmatica CCMP1168 chloroplast, complete genome |
| bin_352 | Bacteria | Actinobacteria | Actinobacteria | 69.73 | 5.47 | 1142560 | 222 | no_ssu |
| bin_353 | Bacteria | Proteobacteria | Alphaproteobacteria | 48.07 | 5.36 | 2015767 | 620 | no_ssu |
| bin_354 | Bacteria | Bacteroidetes | Flavobacteriia | 74.45 | 60.39 | 5338197 | 1136 | no_ssu |
| bin_355 | Bacteria | Bacteroidetes | Flavobacteriia | 4.17 | 0 | 471172 | 88 | no_ssu |
| bin_356 | Bacteria | Proteobacteria | Gammaproteobacteria | 59.62 | 1.85 | 895989 | 246 | no_ssu |
| bin_357 | Bacteria | Actinobacteria | Actinobacteria | 52.8 | 3.44 | 775148 | 214 | no_ssu |
| bin_358 | Bacteria | Actinobacteria | Actinobacteria | 53.45 | 18.1 | 848430 | 256 | no_ssu |
| bin_359 | Bacteria | Proteobacteria | Alphaproteobacteria | 3.51 | 0.58 | 314972 | 70 | no_ssu |
| bin_36 | Bacteria | Proteobacteria | Gammaproteobacteria | 0 | 0 | 433507 | 116 | no_ssu |
| bin_360 | Bacteria | Actinobacteria | Actinobacteria | 20.98 | 0 | 685320 | 220 | no_ssu |
| bin_361 | Bacteria | Proteobacteria | Gammaproteobacteria | 12.41 | 0.63 | 1180568 | 303 | no_ssu |
| bin_362 | Bacteria | Proteobacteria | Gammaproteobacteria | 27.41 | 0 | 384603 | 96 | no_ssu |
| bin_363 | Bacteria | Cyanobacteria | NA | 94.73 | 170.74 | 13255693 | 2473 | no_ssu |
| bin_364 | Bacteria | Bacteroidetes | Flavobacteriia | 88.2 | 6.67 | 2762063 | 338 | no_ssu |
| bin_365 | Bacteria | Proteobacteria | Gammaproteobacteria | 0 | 0 | 337377 | 75 | no_ssu |
| bin_366 | Bacteria | Bacteroidetes | Flavobacteriia | 31.05 | 5.66 | 900866 | 242 | no_ssu |
| bin_367 | Bacteria | Cyanobacteria | NA | 80.71 | 1.9 | 3860594 | 866 | no_ssu |
| bin_368 | Bacteria | Proteobacteria | Gammaproteobacteria | 70.86 | 11.21 | 3032000 | 574 | no_ssu |
| bin_369 | Bacteria | Proteobacteria | Alphaproteobacteria | 1.72 | 0 | 204928 | 42 | no_ssu |
| bin_37 | Bacteria | Bacteroidetes | Flavobacteriia | 41.49 | 0 | 1118567 | 242 | no_ssu |
| bin_370 | Bacteria | Proteobacteria | Alphaproteobacteria | 1.29 | 0.93 | 592810 | 91 | no_ssu |
| bin_371 | Bacteria | Tenericutes | Mollicutes | 1.68 | 0 | 290322 | 52 | no_ssu |
| bin_372 | Bacteria | Firmicutes | Bacilli | 0 | 0 | 247763 | 38 | no_ssu |
| bin_373 | Bacteria | Proteobacteria | Gammaproteobacteria | 0 | 0 | 281984 | 56 | no_ssu |
| bin_374 | Bacteria | Firmicutes | Bacilli | 63.77 | 25.09 | 1202471 | 340 | no_ssu |
| bin_375 | Bacteria | Firmicutes | Erysipelotrichia | 3.98 | 0 | 323136 | 29 | no_ssu |
| bin_376 | Bacteria | Proteobacteria | Gammaproteobacteria | 32.89 | 2.59 | 569157 | 140 | no_ssu |
| bin_377 | Bacteria | Proteobacteria | Alphaproteobacteria | 23.95 | 1.33 | 273246 | 67 | no_ssu |
| bin_378 | Bacteria | Proteobacteria | Oligoflexia | 93.7 | 1.79 | 4012872 | 132 | no_ssu |
| bin_379 | Bacteria | Proteobacteria | Gammaproteobacteria | 7.05 | 0 | 535324 | 205 | Uncultured bacterium clone DP7.4.14 16S ribosomal RNA gene, partial sequence |
| bin_38 | Bacteria | Proteobacteria | Alphaproteobacteria | 70.29 | 42.18 | 2031662 | 411 | no_ssu |
| bin_380 | Bacteria | Proteobacteria | Alphaproteobacteria | 77.28 | 2.41 | 3128610 | 669 | Uncultured bacterium clone 2E-021 16S ribosomal RNA gene, partial sequence |
| bin_381 | Bacteria | Bacteroidetes | Flavobacteriia | 58.44 | 8.13 | 1210240 | 228 | no_ssu |
| bin_382 | Bacteria | Proteobacteria | Alphaproteobacteria | 0 | 0 | 329126 | 49 | no_ssu |
| bin_383 | Bacteria | Proteobacteria | Gammaproteobacteria | 91.25 | 1.36 | 3567317 | 73 | Uncultured bacterium clone NN44 16S ribosomal RNA gene, partial sequence |
| bin_384 | Bacteria | Firmicutes | Bacilli | 1.34 | 0 | 245454 | 85 | no_ssu |
| bin_385 | Bacteria | Actinobacteria | Actinobacteria | 53.61 | 5.17 | 674194 | 106 | no_ssu |
| bin_386 | Bacteria | Proteobacteria | Gammaproteobacteria | 81 | 2.87 | 1992184 | 340 | Uncultured bacterium clone 8EP-60 16S ribosomal RNA gene, partial sequence |
| bin_387 | Bacteria | Actinobacteria | Actinobacteria | 38.5 | 0.85 | 1808107 | 527 | no_ssu |
| bin_388 | Bacteria | Proteobacteria | Epsilonproteobacteria | 10.34 | 0 | 739461 | 254 | Uncultured diatom clone Re21-16S_14 16S ribosomal RNA gene, partial sequence; chloroplast |
| bin_389 | Bacteria | Proteobacteria | Alphaproteobacteria | 17.3 | 0 | 1382991 | 469 | no_ssu |
| bin_39 | Bacteria | Proteobacteria | Alphaproteobacteria | 82.42 | 85.69 | 8840939 | 2039 | Rhodobacter capsulatus SB 1003, complete genome |
| bin_390 | Bacteria | Proteobacteria | Gammaproteobacteria | 0 | 0 | 244136 | 49 | no_ssu |
| bin_391 | Bacteria | Firmicutes | Bacilli | 3.45 | 0.86 | 217294 | 63 | no_ssu |
| bin_392 | Bacteria | Proteobacteria | Alphaproteobacteria | 36.22 | 1.14 | 2304537 | 715 | no_ssu |
| bin_393 | Bacteria | Proteobacteria | Alphaproteobacteria | 35.34 | 1.72 | 1229905 | 421 | no_ssu |
| bin_394 | Bacteria | Proteobacteria | Gammaproteobacteria | 95.6 | 2.7 | 2559813 | 111 | no_ssu |
| bin_395 | Bacteria | Bacteroidetes | Flavobacteriia | 63.85 | 2.92 | 1202735 | 278 | no_ssu |
| bin_396 | Bacteria | Proteobacteria | Betaproteobacteria | 71.58 | 6.69 | 2222807 | 574 | Ramlibacter tataouinensis strain 5-10, complete genome |
| bin_397 | Bacteria | Proteobacteria | Gammaproteobacteria | 16.14 | 4.36 | 2161599 | 662 | Uncultured delta proteobacterium HF0130_05G09 genomic sequence |
| bin_398 | Bacteria | Cyanobacteria | NA | 5.78 | 1.08 | 384768 | 75 | no_ssu |
| bin_399 | Bacteria | Proteobacteria | Alphaproteobacteria | 8.33 | 4.17 | 1500150 | 497 | no_ssu |
| bin_4 | Bacteria | Bacteroidetes | Flavobacteriia | 98.53 | 0.25 | 1964342 | 93 | no_ssu |
| bin_40 | Bacteria | Proteobacteria | Gammaproteobacteria | 34.48 | 0.86 | 1403057 | 436 | no_ssu |
| bin_400 | Bacteria | Proteobacteria | Alphaproteobacteria | 0 | 0 | 378223 | 88 | no_ssu |
| bin_401 | Bacteria | Proteobacteria | Gammaproteobacteria | 1.15 | 0 | 481639 | 158 | no_ssu |
| bin_402 | Bacteria | Cyanobacteria | NA | 38.4 | 0.09 | 1356214 | 426 | no_ssu |
| bin_403 | Bacteria | Proteobacteria | Alphaproteobacteria | 96.69 | 3.5 | 3790081 | 205 | Uncultured bacterium clone A8S-99 16S ribosomal RNA gene, partial sequence |
| bin_404 | Bacteria | Proteobacteria | Alphaproteobacteria | 71.7 | 10.92 | 4104180 | 1129 | no_ssu |
| bin_405 | Bacteria | Firmicutes | Bacilli | 2.66 | 0 | 361610 | 51 | no_ssu |
| bin_406 | Bacteria | Actinobacteria | Actinobacteria | 45.77 | 3.45 | 1302723 | 371 | no_ssu |
| bin_407 | Bacteria | Proteobacteria | Betaproteobacteria | 8.56 | 1.72 | 484474 | 136 | no_ssu |
| bin_408 | Bacteria | Firmicutes | Bacilli | 3.04 | 0.5 | 492817 | 83 | no_ssu |
| bin_409 | Bacteria | Actinobacteria | Actinobacteria | 40.53 | 1.75 | 810466 | 222 | no_ssu |
| bin_41 | Bacteria | Actinobacteria | NA | 10.42 | 0 | 361222 | 111 | no_ssu |
| bin_410 | Bacteria | Cyanobacteria | NA | 7.27 | 0 | 383862 | 114 | no_ssu |
| bin_411 | Bacteria | Actinobacteria | Actinobacteria | 58.62 | 5.4 | 1616615 | 422 | no_ssu |
| bin_412 | Bacteria | Cyanobacteria | NA | 32.41 | 100.67 | 1018901 | 269 | Uncultured bacterium clone A12 16S ribosomal RNA gene, partial sequence |
| bin_413 | Bacteria | Proteobacteria | Gammaproteobacteria | 2.52 | 0.43 | 380090 | 95 | no_ssu |
| bin_414 | Bacteria | Firmicutes | Tissierellia | 3.37 | 0 | 405959 | 81 | no_ssu |
| bin_415 | Bacteria | Proteobacteria | Gammaproteobacteria | 0 | 0 | 309448 | 22 | no_ssu |
| bin_416 | Bacteria | Proteobacteria | Alphaproteobacteria | 59.37 | 1.34 | 5230900 | 1272 | Uncultured planctomycete clone OTU-X1-24 16S ribosomal RNA gene, partial sequence |
| bin_417 | Bacteria | Bacteroidetes | Flavobacteriia | 17.92 | 0 | 214562 | 72 | no_ssu |
| bin_418 | Bacteria | Proteobacteria | Gammaproteobacteria | 0 | 0 | 395661 | 84 | no_ssu |
| bin_419 | Archaea | Euryarchaeota | Methanobacteria | 0.79 | 0.22 | 249550 | 61 | no_ssu |
| bin_42 | Bacteria | Bacteroidetes | Flavobacteriia | 88.23 | 74.75 | 3284205 | 793 | no_ssu |
| bin_420 | Bacteria | Proteobacteria | Alphaproteobacteria | 40.57 | 3.77 | 768744 | 168 | no_ssu |
| bin_421 | Bacteria | Bacteroidetes | Flavobacteriia | 84.29 | 5.86 | 2543685 | 481 | Uncultured bacterium clone RP8_18 16S ribosomal RNA gene, partial sequence |
| bin_422 | Bacteria | Bacteroidetes | Flavobacteriia | 0 | 0 | 228652 | 29 | no_ssu |
| bin_423 | Bacteria | Proteobacteria | Alphaproteobacteria | 78.39 | 5.75 | 2142821 | 493 | Uncultured bacterium clone K08-108-BAC 16S ribosomal RNA gene, partial sequence |
| bin_424 | Bacteria | Proteobacteria | Alphaproteobacteria | 28.39 | 1.89 | 315597 | 83 | no_ssu |
| bin_425 | Bacteria | Proteobacteria | Betaproteobacteria | 8.62 | 1.72 | 288092 | 74 | no_ssu |
| bin_426 | Bacteria | Firmicutes | Bacilli | 0 | 0 | 311226 | 24 | no_ssu |
| bin_427 | Bacteria | Proteobacteria | Gammaproteobacteria | 61.86 | 22.14 | 2123021 | 657 | no_ssu |
| bin_428 | Bacteria | Proteobacteria | Betaproteobacteria | 15.52 | 0 | 646665 | 224 | no_ssu |
| bin_429 | Bacteria | Firmicutes | Bacilli | 3.04 | 0.5 | 455019 | 30 | no_ssu |
| bin_43 | Bacteria | Actinobacteria | Actinobacteria | 92.31 | 1.28 | 3084068 | 96 | Uncultured bacterium clone LX 34 16S ribosomal RNA gene, partial sequence |
| bin_430 | Bacteria | Actinobacteria | Actinobacteria | 23.59 | 8.62 | 1483467 | 497 | no_ssu |
| bin_431 | Bacteria | Proteobacteria | Gammaproteobacteria | 95.94 | 1.41 | 2768830 | 98 | Uncultured bacterium partial 16S rRNA gene, clone SIFF475_N9D4_16S_B |
| bin_432 | Bacteria | Actinobacteria | Actinobacteria | 70.08 | 3.7 | 2024209 | 314 | no_ssu |
| bin_433 | Bacteria | Actinobacteria | Actinobacteria | 4.17 | 0 | 320857 | 86 | no_ssu |
| bin_434 | Bacteria | Proteobacteria | Alphaproteobacteria | 27.59 | 3.45 | 1382559 | 477 | no_ssu |
| bin_435 | Bacteria | Proteobacteria | Alphaproteobacteria | 20.3 | 0 | 1165603 | 394 | no_ssu |
| bin_436 | Bacteria | Proteobacteria | Alphaproteobacteria | 5.39 | 1.58 | 914937 | 247 | no_ssu |
| bin_437 | Bacteria | Verrucomicrobia | Opitutae | 91.95 | 162.08 | 16068030 | 4943 | no_ssu |
| bin_438 | Bacteria | Bacteroidetes | Flavobacteriia | 98.57 | 0.24 | 2799814 | 198 | Uncultured bacterium RNA for 16S rRNA, partial sequence, clone: B0618R003_N12 |
| bin_439 | Bacteria | Bacteroidetes | Flavobacteriia | 98.13 | 1.66 | 1809900 | 51 | no_ssu |
| bin_44 | Bacteria | Proteobacteria | Gammaproteobacteria | 88.76 | 3.49 | 5105912 | 714 | no_ssu |
| bin_440 | Bacteria | Bacteroidetes | Flavobacteriia | 93.75 | 54.1 | 3849159 | 869 | no_ssu |
| bin_441 | Bacteria | Proteobacteria | Gammaproteobacteria | 88.39 | 1.79 | 2598878 | 342 | no_ssu |
| bin_442 | Bacteria | Proteobacteria | Betaproteobacteria | 95.73 | 0.69 | 1947182 | 155 | no_ssu |
| bin_443 | Bacteria | Proteobacteria | Alphaproteobacteria | 60.01 | 21.33 | 2702452 | 914 | no_ssu |
| bin_444 | Bacteria | Proteobacteria | Alphaproteobacteria | 38.17 | 0.86 | 1903828 | 638 | no_ssu |
| bin_445 | Bacteria | Proteobacteria | Alphaproteobacteria | 35.85 | 6.6 | 771496 | 193 | no_ssu |
| bin_446 | Bacteria | Proteobacteria | Gammaproteobacteria | 97.3 | 0 | 2081352 | 114 | no_ssu |
| bin_447 | Bacteria | Proteobacteria | Gammaproteobacteria | 34.71 | 0.57 | 496804 | 106 | no_ssu |
| bin_448 | Bacteria | Actinobacteria | Actinobacteria | 69.07 | 5.46 | 1169837 | 216 | Uncultured actinobacterium clone WA0.2-0d-65 16S ribosomal RNA gene, partial sequence |
| bin_449 | Bacteria | Actinobacteria | Actinobacteria | 85.58 | 92.18 | 3846573 | 1032 | no_ssu |
| bin_45 | Bacteria | Firmicutes | Clostridia | 0.83 | 0 | 273648 | 21 | no_ssu |
| bin_450 | Bacteria | Proteobacteria | Alphaproteobacteria | 86.82 | 11.02 | 3642285 | 436 | no_ssu |
| bin_451 | Bacteria | Firmicutes | Clostridia | 53.92 | 1.75 | 532149 | 100 | Uncultured marine bacterium clone S21-12 16S ribosomal RNA gene, partial sequence |
| bin_452 | Bacteria | Firmicutes | Bacilli | 50.45 | 1.71 | 659798 | 185 | no_ssu |
| bin_453 | Bacteria | Proteobacteria | Gammaproteobacteria | 43.65 | 0 | 1349300 | 369 | no_ssu |
| bin_454 | Bacteria | Firmicutes | Bacilli | 0.49 | 0 | 290313 | 81 | no_ssu |
| bin_455 | Bacteria | Proteobacteria | Gammaproteobacteria | 29.26 | 11.15 | 623022 | 167 | Uncultured gamma proteobacterium clone ARTE1_102 16S ribosomal RNA gene, partial sequence |
| bin_456 | Bacteria | Proteobacteria | Gammaproteobacteria | 17.34 | 0 | 317174 | 103 | no_ssu |
| bin_457 | Bacteria | Chloroflexi | Caldilineae | 93.18 | 3.94 | 4261663 | 478 | no_ssu |
| bin_458 | Bacteria | Cyanobacteria | NA | 67.02 | 6.58 | 1666026 | 328 | no_ssu |
| bin_459 | Bacteria | Firmicutes | Bacilli | 4.45 | 0.58 | 520855 | 77 | no_ssu |
| bin_46 | Bacteria | Proteobacteria | Gammaproteobacteria | 0 | 0 | 600658 | 98 | no_ssu |
| bin_460 | Bacteria | Proteobacteria | Betaproteobacteria | 45.78 | 7.05 | 795840 | 237 | no_ssu |
| bin_461 | Bacteria | Proteobacteria | Gammaproteobacteria | 38.79 | 1.72 | 1258729 | 376 | no_ssu |
| bin_462 | Bacteria | Actinobacteria | Actinobacteria | 25.63 | 4.14 | 1144174 | 412 | no_ssu |
| bin_463 | Bacteria | Bacteroidetes | Flavobacteriia | 23.82 | 1.72 | 496071 | 152 | no_ssu |
| bin_464 | Bacteria | Actinobacteria | Actinobacteria | 59.91 | 1.72 | 851402 | 198 | no_ssu |
| bin_465 | Bacteria | Firmicutes | Bacilli | 0 | 0 | 234960 | 26 | no_ssu |
| bin_466 | Bacteria | Proteobacteria | Gammaproteobacteria | 92.03 | 2.08 | 4195391 | 582 | no_ssu |
| bin_467 | Bacteria | Proteobacteria | Gammaproteobacteria | 82.48 | 1.71 | 1337764 | 189 | Uncultured bacterium clone DWI02D 16S ribosomal RNA gene, partial sequence |
| bin_468 | Bacteria | Fusobacteria | Fusobacteriia | 5.44 | 0.52 | 435932 | 17 | no_ssu |
| bin_469 | Bacteria | Proteobacteria | Gammaproteobacteria | 3.13 | 0 | 272071 | 49 | no_ssu |
| bin_47 | Bacteria | Actinobacteria | Actinobacteria | 77.32 | 40.34 | 4105151 | 1369 | Uncultured actinobacterium clone THAD2-32 16S ribosomal RNA gene, partial sequence |
| bin_470 | Bacteria | Actinobacteria | Actinobacteria | 26.63 | 2.3 | 1237400 | 445 | no_ssu |
| bin_471 | Bacteria | Bacteroidetes | Flavobacteriia | 18.45 | 5.17 | 589285 | 163 | no_ssu |
| bin_472 | Bacteria | Proteobacteria | Gammaproteobacteria | 4.3 | 0.5 | 462497 | 65 | no_ssu |
| bin_473 | Bacteria | Proteobacteria | Gammaproteobacteria | 2.28 | 0.07 | 510854 | 105 | no_ssu |
| bin_474 | Bacteria | Proteobacteria | Gammaproteobacteria | 2.23 | 0 | 332094 | 68 | no_ssu |
| bin_475 | Bacteria | Cyanobacteria | NA | 83.64 | 6.03 | 1898294 | 260 | Uncultured bacterium clone T6_0611_49 16S ribosomal RNA gene, partial sequence |
| bin_476 | Bacteria | Bacteroidetes | Flavobacteriia | 86.74 | 37.03 | 2681058 | 625 | no_ssu |
| bin_477 | Bacteria | Proteobacteria | Gammaproteobacteria | 95.69 | 2.76 | 3167559 | 210 | Thiohalobacter thiocyanaticus DNA, complete genome, strain: FOKN1 |
| bin_478 | Bacteria | Proteobacteria | Betaproteobacteria | 25.08 | 0 | 1209264 | 170 | no_ssu |
| bin_479 | Bacteria | Actinobacteria | Actinobacteria | 86.47 | 3.48 | 2231887 | 222 | no_ssu |
| bin_48 | Bacteria | Actinobacteria | Actinobacteria | 76.27 | 3.51 | 1848521 | 355 | no_ssu |
| bin_480 | Bacteria | Proteobacteria | Gammaproteobacteria | 65.35 | 8.81 | 1146425 | 266 | Uncultured bacterium clone 6C233358 16S ribosomal RNA gene, partial sequence |
| bin_481 | Bacteria | Proteobacteria | Alphaproteobacteria | 91.01 | 1.31 | 3188671 | 494 | Uncultured planctomycete clone BL11-5 16S ribosomal RNA gene, partial sequence |
| bin_482 | Bacteria | Actinobacteria | Actinobacteria | 67.74 | 4.31 | 1479562 | 195 | no_ssu |
| bin_483 | Bacteria | Proteobacteria | Alphaproteobacteria | 50.5 | 3.45 | 1885263 | 551 | no_ssu |
| bin_484 | Bacteria | Proteobacteria | Alphaproteobacteria | 3.16 | 0.47 | 975858 | 357 | Uncultured bacterium clone N2_046 16S ribosomal RNA gene, partial sequence |
| bin_485 | Bacteria | Actinobacteria | Actinobacteria | 35.42 | 0 | 576998 | 178 | no_ssu |
| bin_486 | Bacteria | Cyanobacteria | NA | 25 | 0 | 1169839 | 248 | no_ssu |
| bin_487 | Bacteria | Firmicutes | Bacilli | 0.22 | 0 | 921646 | 249 | no_ssu |
| bin_488 | Bacteria | Proteobacteria | Alphaproteobacteria | 71.47 | 3.2 | 2499065 | 674 | no_ssu |
| bin_489 | Bacteria | Proteobacteria | Alphaproteobacteria | 87.36 | 62.8 | 6763439 | 1165 | Uncultured bacterium clone F5K2Q4C04I7N0W 16S ribosomal RNA gene, partial sequence |
| bin_49 | Bacteria | Actinobacteria | Actinobacteria | 0 | 0 | 315038 | 55 | no_ssu |
| bin_490 | Bacteria | Bacteroidetes | Flavobacteriia | 75.47 | 4.55 | 2313918 | 557 | no_ssu |
| bin_491 | Bacteria | Bacteroidetes | Flavobacteriia | 77.62 | 1.66 | 1586770 | 299 | Uncultured Bacteroidetes bacterium clone LSB-E08 16S ribosomal RNA gene, partial sequence |
| bin_492 | Bacteria | Proteobacteria | Alphaproteobacteria | 77.07 | 0.8 | 1765902 | 86 | Uncultured marine group II euryarchaeote clone PU032005arc1aE7 16S ribosomal RNA gene, partial sequence |
| bin_493 | Bacteria | Firmicutes | Bacilli | 23.14 | 2.7 | 1494700 | 292 | no_ssu |
| bin_494 | Bacteria | Proteobacteria | Gammaproteobacteria | 8.96 | 5.39 | 2043038 | 588 | Uncultured marine bacterium clone OTU34 16S ribosomal RNA gene, partial sequence |
| bin_495 | Bacteria | Proteobacteria | Alphaproteobacteria | 56.46 | 2.19 | 2334920 | 518 | Uncultured bacterium clone H02_SB4A 16S ribosomal RNA gene, partial sequence |
| bin_496 | Bacteria | Proteobacteria | Betaproteobacteria | 6.9 | 0 | 846904 | 259 | no_ssu |
| bin_497 | Bacteria | Actinobacteria | Actinobacteria | 23.19 | 0 | 696999 | 165 | no_ssu |
| bin_498 | Bacteria | Bacteroidetes | Flavobacteriia | 95.08 | 1.61 | 2288276 | 157 | no_ssu |
| bin_499 | Bacteria | Proteobacteria | Gammaproteobacteria | 79.33 | 1.07 | 1831872 | 213 | Uncultured marine group II euryarchaeote HF10_15F05 16S small subunit ribosomal RNA gene, partial sequence |
| bin_5 | Bacteria | Proteobacteria | Gammaproteobacteria | 66.38 | 16.38 | 3112040 | 764 | no_ssu |
| bin_50 | Bacteria | Firmicutes | Bacilli | 3.22 | 0 | 926649 | 178 | no_ssu |
| bin_500 | Bacteria | Bacteroidetes | Cytophagia | 88.51 | 86.21 | 5301534 | 325 | Uncultured bacterium clone 3C002442 16S ribosomal RNA gene, partial sequence |
| bin_501 | Bacteria | Bacteroidetes | Flavobacteriia | 5.38 | 0.5 | 437353 | 16 | no_ssu |
| bin_502 | Bacteria | Bacteroidetes | Cytophagia | 18.09 | 0 | 639713 | 204 | no_ssu |
| bin_503 | Bacteria | Proteobacteria | Gammaproteobacteria | 82.47 | 4.24 | 2072752 | 405 | Uncultured bacterium clone BL_Sep2011_2,5m_H12 16S ribosomal RNA gene, partial sequence |
| bin_504 | Bacteria | Firmicutes | Bacilli | 63.91 | 19.21 | 824656 | 238 | no_ssu |
| bin_505 | Bacteria | Proteobacteria | Betaproteobacteria | 84.64 | 5.91 | 3258836 | 381 | no_ssu |
| bin_506 | Bacteria | Actinobacteria | Actinobacteria | 69.59 | 13.13 | 1992821 | 545 | Uncultured bacterium clone Bbef-02F12 16S ribosomal RNA gene, partial sequence |
| bin_507 | Bacteria | Actinobacteria | Actinobacteria | 49.47 | 8.62 | 1754341 | 530 | no_ssu |
| bin_508 | Bacteria | Proteobacteria | Alphaproteobacteria | 44.7 | 7.74 | 1043067 | 249 | no_ssu |
| bin_509 | Bacteria | Proteobacteria | Gammaproteobacteria | 4.45 | 0.65 | 554950 | 121 | no_ssu |
| bin_51 | Bacteria | Actinobacteria | Actinobacteria | 94.36 | 7.53 | 1985595 | 213 | no_ssu |
| bin_510 | Bacteria | Actinobacteria | Actinobacteria | 40.35 | 0 | 518621 | 154 | no_ssu |
| bin_511 | Bacteria | Firmicutes | Bacilli | 43.42 | 0 | 693393 | 230 | no_ssu |
| bin_512 | Bacteria | Firmicutes | Bacilli | 0 | 0 | 740664 | 178 | no_ssu |
| bin_513 | Bacteria | Proteobacteria | Alphaproteobacteria | 27.59 | 6.9 | 909083 | 327 | no_ssu |
| bin_514 | Bacteria | Actinobacteria | Actinobacteria | 87.63 | 3.92 | 1876610 | 257 | Uncultured bacterium clone 8 16S ribosomal RNA gene, partial sequence |
| bin_515 | Bacteria | Proteobacteria | Alphaproteobacteria | 84.05 | 2.46 | 2147903 | 355 | Bacterium enrichment culture clone HB_62 16S ribosomal RNA gene, partial sequence |
| bin_516 | Bacteria | Bacteroidetes | Flavobacteriia | 99.18 | 0.11 | 2923732 | 88 | no_ssu |
| bin_517 | Bacteria | Proteobacteria | Alphaproteobacteria | 66.5 | 2.43 | 2429317 | 624 | no_ssu |
| bin_518 | Bacteria | Proteobacteria | Gammaproteobacteria | 41.87 | 8.41 | 644432 | 185 | no_ssu |
| bin_519 | Bacteria | Proteobacteria | Betaproteobacteria | 89.06 | 70.58 | 3021927 | 807 | no_ssu |
| bin_52 | Bacteria | Proteobacteria | Alphaproteobacteria | 51.59 | 1.39 | 1084697 | 327 | no_ssu |
| bin_520 | Bacteria | Proteobacteria | Gammaproteobacteria | 87.73 | 0.36 | 3090498 | 280 | no_ssu |
| bin_521 | Bacteria | Proteobacteria | Gammaproteobacteria | 75.86 | 0 | 963416 | 46 | Uncultured gamma proteobacterium clone EC3 16S ribosomal RNA gene, partial sequence |
| bin_522 | Bacteria | Bacteroidetes | Flavobacteriia | 54.54 | 1.98 | 2158231 | 623 | no_ssu |
| bin_523 | Bacteria | Bacteroidetes | Flavobacteriia | 0.79 | 0 | 211585 | 38 | no_ssu |
| bin_524 | Bacteria | Cyanobacteria | NA | 0 | 0 | 599628 | 161 | no_ssu |
| bin_525 | Bacteria | Cyanobacteria | NA | 38.1 | 3.62 | 1429493 | 437 | Synechococcus sp. CB0101 chromosome, complete genome |
| bin_526 | Bacteria | Firmicutes | Clostridia | 6.31 | 0.58 | 478854 | 71 | no_ssu |
| bin_527 | Bacteria | Actinobacteria | Actinobacteria | 30.53 | 0.86 | 488409 | 129 | no_ssu |
| bin_528 | Bacteria | Proteobacteria | Gammaproteobacteria | 86.99 | 3.45 | 4096903 | 440 | Uncultured bacterium clone ST27_5m_clone15 16S ribosomal RNA gene, partial sequence |
| bin_529 | Bacteria | Proteobacteria | Alphaproteobacteria | 24.29 | 3.45 | 1771170 | 488 | no_ssu |
| bin_53 | Bacteria | Bacteroidetes | Flavobacteriia | 85.52 | 12.07 | 2819153 | 232 | no_ssu |
| bin_530 | Bacteria | Proteobacteria | Alphaproteobacteria | 91.69 | 6.59 | 3039223 | 253 | Uncultured bacterium clone BJGMM-3s-275 16S ribosomal RNA gene, partial sequence |
| bin_531 | Bacteria | Proteobacteria | Gammaproteobacteria | 4.17 | 0 | 232838 | 67 | no_ssu |
| bin_532 | Bacteria | Cyanobacteria | NA | 79.07 | 6.84 | 1865858 | 314 | Synechococcus sp. CB0101 chromosome, complete genome |
| bin_533 | Bacteria | Firmicutes | Bacilli | 3.38 | 0 | 302754 | 89 | no_ssu |
| bin_534 | Bacteria | Firmicutes | Bacilli | 6.24 | 1.51 | 431965 | 81 | no_ssu |
| bin_535 | Bacteria | Proteobacteria | Alphaproteobacteria | 61.16 | 17.94 | 3603670 | 1165 | no_ssu |
| bin_536 | Bacteria | Proteobacteria | Gammaproteobacteria | 4.28 | 7.99 | 425857 | 95 | no_ssu |
| bin_537 | Bacteria | Proteobacteria | Alphaproteobacteria | 84.14 | 19.11 | 1812758 | 421 | Uncultured bacterium clone H0054 16S ribosomal RNA gene, partial sequence |
| bin_538 | Bacteria | Proteobacteria | Betaproteobacteria | 97.44 | 0.45 | 3302387 | 228 | Uncultured bacterium clone OTU1475 16S ribosomal RNA gene, partial sequence |
| bin_539 | Bacteria | Bacteroidetes | Bacteroidia | 13.79 | 0 | 368820 | 132 | no_ssu |
| bin_54 | Bacteria | Proteobacteria | Deltaproteobacteria | 18.18 | 0 | 608199 | 236 | no_ssu |
| bin_540 | Bacteria | Proteobacteria | Alphaproteobacteria | 79.91 | 8.79 | 4308514 | 683 | Uncultured bacterium 270 genomic sequence |
| bin_541 | Bacteria | Firmicutes | Bacilli | 45.1 | 1.71 | 434128 | 109 | Uncultured bacterium clone Wat38 16S ribosomal RNA gene, partial sequence |
| bin_542 | Bacteria | Proteobacteria | Gammaproteobacteria | 4.17 | 0 | 530941 | 90 | no_ssu |
| bin_543 | Bacteria | Actinobacteria | Actinobacteria | 0.96 | 0 | 367646 | 34 | no_ssu |
| bin_544 | Bacteria | Bacteroidetes | Flavobacteriia | 0 | 0 | 243711 | 59 | no_ssu |
| bin_545 | Bacteria | Actinobacteria | Actinobacteria | 62.54 | 6.9 | 1114816 | 240 | no_ssu |
| bin_546 | Bacteria | Proteobacteria | Gammaproteobacteria | 44.83 | 3.45 | 938579 | 262 | no_ssu |
| bin_547 | Bacteria | Proteobacteria | Gammaproteobacteria | 92.29 | 3.02 | 3406836 | 231 | no_ssu |
| bin_548 | Bacteria | Firmicutes | Bacilli | 3.2 | 0.43 | 415981 | 88 | no_ssu |
| bin_549 | Bacteria | Bacteroidetes | Flavobacteriia | 79.8 | 6.31 | 2183166 | 452 | Panacibacter ginsenosidivorans strain Gsoil1550 chromosome, complete genome |
| bin_55 | Bacteria | Proteobacteria | Gammaproteobacteria | 59.89 | 1.49 | 831242 | 230 | no_ssu |
| bin_550 | Bacteria | Proteobacteria | Gammaproteobacteria | 2.11 | 0.43 | 391864 | 79 | no_ssu |
| bin_551 | Bacteria | Proteobacteria | Alphaproteobacteria | 72.53 | 0.1 | 2407601 | 518 | no_ssu |
| bin_552 | Bacteria | Bacteroidetes | Cytophagia | 94.13 | 0.48 | 2175828 | 69 | no_ssu |
| bin_553 | Bacteria | Proteobacteria | Alphaproteobacteria | 97.7 | 1.54 | 2195083 | 96 | Erythrobacter litoralis strain DSM 8509, complete genome |
| bin_554 | Bacteria | Proteobacteria | Betaproteobacteria | 72.87 | 42.47 | 3106663 | 1065 | no_ssu |
| bin_555 | NA | NA | NA | 1.8 | 0 | 443685 | 17 | no_ssu |
| bin_556 | Bacteria | Bacteroidetes | Flavobacteriia | 95.54 | 1.09 | 2706514 | 242 | Uncultured Bacteroidetes bacterium clone WA0.2-0d-18 16S ribosomal RNA gene, partial sequence |
| bin_557 | Bacteria | Proteobacteria | Gammaproteobacteria | 39.59 | 8.05 | 833795 | 232 | no_ssu |
| bin_558 | Bacteria | Firmicutes | Bacilli | 7.25 | 0.5 | 507052 | 60 | no_ssu |
| bin_559 | Bacteria | Proteobacteria | Alphaproteobacteria | 86.11 | 3.85 | 2052547 | 367 | Uncultured soil bacterium 16S rRNA gene, clone H4_T0-11 |
| bin_56 | Bacteria | Firmicutes | Bacilli | 0 | 0 | 738290 | 138 | no_ssu |
| bin_560 | Bacteria | Actinobacteria | Actinobacteria | 21.87 | 1.72 | 574351 | 169 | no_ssu |
| bin_561 | Bacteria | Bacteroidetes | Flavobacteriia | 84.53 | 6.92 | 1670060 | 339 | no_ssu |
| bin_562 | Bacteria | Bacteroidetes | Flavobacteriia | 90.09 | 0.47 | 1831420 | 34 | no_ssu |
| bin_563 | Bacteria | Proteobacteria | Alphaproteobacteria | 93.44 | 3.93 | 2906992 | 160 | Uncultured marine bacterium clone B17-28 16S ribosomal RNA gene, partial sequence |
| bin_564 | Bacteria | Cyanobacteria | NA | 0 | 0 | 578755 | 102 | no_ssu |
| bin_565 | Bacteria | Proteobacteria | Gammaproteobacteria | 99.36 | 63.02 | 6972958 | 1315 | Uncultured bacterium clone EyjYyy78 16S ribosomal RNA gene, partial sequence |
| bin_566 | Bacteria | Firmicutes | Bacilli | 45.92 | 9.34 | 12749159 | 4305 | Chlorella heliozoae plastid, complete genome |
| bin_567 | Bacteria | Actinobacteria | Actinobacteria | 95.53 | 29.91 | 2844717 | 465 | Uncultured actinobacterium clone OOB120 16S ribosomal RNA gene, partial sequence |
| bin_568 | Bacteria | Cyanobacteria | NA | 31.94 | 5.17 | 1701114 | 454 | no_ssu |
| bin_569 | Bacteria | Proteobacteria | Gammaproteobacteria | 53.32 | 6.86 | 1302530 | 373 | no_ssu |
| bin_57 | Bacteria | Proteobacteria | Alphaproteobacteria | 95.78 | 1.52 | 2895419 | 242 | Yangia pacifica strain YSBP01 plasmid unnamed2, complete sequence |
| bin_570 | Bacteria | Proteobacteria | Gammaproteobacteria | 20.69 | 3.45 | 861483 | 283 | no_ssu |
| bin_571 | Bacteria | Proteobacteria | Betaproteobacteria | 33.1 | 0 | 1438352 | 461 | no_ssu |
| bin_572 | Bacteria | Proteobacteria | Gammaproteobacteria | 70.56 | 1.35 | 4257461 | 796 | no_ssu |
| bin_573 | Bacteria | Cyanobacteria | NA | 31.03 | 0.86 | 1067526 | 249 | no_ssu |
| bin_574 | Bacteria | Proteobacteria | Betaproteobacteria | 95.79 | 9.35 | 1276844 | 144 | Methylophilaceae bacterium NB0092 16S ribosomal RNA gene, partial sequence |
| bin_575 | Bacteria | Bacteroidetes | Flavobacteriia | 88.51 | 4.3 | 2356501 | 116 | no_ssu |
| bin_576 | Bacteria | Actinobacteria | Actinobacteria | 3.45 | 0 | 259186 | 66 | no_ssu |
| bin_577 | Bacteria | Proteobacteria | Betaproteobacteria | 89.82 | 2.68 | 1406333 | 162 | no_ssu |
| bin_578 | Bacteria | Proteobacteria | Alphaproteobacteria | 44.12 | 1.72 | 1762449 | 547 | no_ssu |
| bin_579 | Bacteria | Proteobacteria | Gammaproteobacteria | 79.62 | 1.41 | 4296339 | 844 | no_ssu |
| bin_58 | Bacteria | Proteobacteria | Alphaproteobacteria | 86.13 | 2.09 | 2236312 | 383 | no_ssu |
| bin_580 | Bacteria | Actinobacteria | Actinobacteria | 61.65 | 5.34 | 1171354 | 224 | no_ssu |
| bin_581 | Bacteria | Proteobacteria | Betaproteobacteria | 93.59 | 6.28 | 2003590 | 244 | no_ssu |
| bin_582 | Bacteria | Proteobacteria | Gammaproteobacteria | 26.03 | 0 | 1282083 | 275 | Uncultured marine bacterium clone S17-75 16S ribosomal RNA gene, partial sequence |
| bin_583 | Bacteria | Firmicutes | Bacilli | 6.17 | 0.5 | 426678 | 33 | no_ssu |
| bin_584 | Bacteria | Actinobacteria | Actinobacteria | 74.08 | 3.41 | 1017291 | 135 | Candidatus Planktophila sp. MMS-VB-114, complete genome |
| bin_585 | Bacteria | Actinobacteria | Actinobacteria | 16.77 | 0 | 293724 | 108 | no_ssu |
| bin_586 | Bacteria | Proteobacteria | Betaproteobacteria | 70.71 | 20.83 | 3509495 | 849 | no_ssu |
| bin_587 | Bacteria | Proteobacteria | Gammaproteobacteria | 79.31 | 5.22 | 3437310 | 776 | no_ssu |
| bin_588 | Bacteria | Firmicutes | Bacilli | 3.59 | 0.5 | 284730 | 78 | no_ssu |
| bin_589 | Bacteria | Proteobacteria | Alphaproteobacteria | 64.51 | 0.5 | 2545481 | 589 | no_ssu |
| bin_59 | Bacteria | Firmicutes | Clostridia | 17.48 | 1.88 | 232168 | 73 | no_ssu |
| bin_590 | Bacteria | Proteobacteria | Gammaproteobacteria | 44.92 | 1.37 | 633795 | 138 | no_ssu |
| bin_591 | Bacteria | Proteobacteria | Gammaproteobacteria | 0 | 0 | 373221 | 124 | no_ssu |
| bin_592 | Bacteria | Cyanobacteria | NA | 80.97 | 28.05 | 2254921 | 457 | no_ssu |
| bin_593 | Bacteria | Proteobacteria | Betaproteobacteria | 33.87 | 5.88 | 1575849 | 388 | no_ssu |
| bin_594 | Bacteria | Proteobacteria | Alphaproteobacteria | 70.73 | 2.13 | 819673 | 192 | Uncultured bacterium clone 265_361 16S ribosomal RNA gene, partial sequence |
| bin_595 | Bacteria | Actinobacteria | Actinobacteria | 79.9 | 26.29 | 1658589 | 350 | Nakamurella sp. s14-144 chromosome, complete genome |
| bin_596 | Bacteria | Firmicutes | Bacilli | 4.34 | 0.29 | 519168 | 138 | no_ssu |
| bin_597 | Bacteria | Proteobacteria | Gammaproteobacteria | 68.34 | 2.66 | 1836608 | 464 | Uncultured marine bacterium clone GD-C1 16S ribosomal RNA gene, partial sequence |
| bin_598 | Bacteria | Proteobacteria | Betaproteobacteria | 93.52 | 168.1 | 8563719 | 2099 | no_ssu |
| bin_599 | Bacteria | Firmicutes | Bacilli | 4.66 | 0.93 | 250557 | 67 | no_ssu |
| bin_6 | Bacteria | Proteobacteria | Gammaproteobacteria | 0.97 | 0 | 215328 | 28 | no_ssu |
| bin_60 | Bacteria | Proteobacteria | Gammaproteobacteria | 56.77 | 2.35 | 2309333 | 636 | no_ssu |
| bin_600 | Bacteria | Firmicutes | Bacilli | 4.84 | 0 | 745699 | 91 | no_ssu |
| bin_601 | Bacteria | Proteobacteria | Alphaproteobacteria | 85.33 | 6.71 | 2669610 | 604 | no_ssu |
| bin_602 | Bacteria | Actinobacteria | Actinobacteria | 67.56 | 4.47 | 1140503 | 153 | no_ssu |
| bin_603 | Bacteria | Proteobacteria | Betaproteobacteria | 34.2 | 0.58 | 2569544 | 636 | no_ssu |
| bin_604 | Bacteria | Proteobacteria | Gammaproteobacteria | 14.19 | 0 | 783829 | 303 | no_ssu |
| bin_605 | Bacteria | Firmicutes | Bacilli | 0 | 0 | 243155 | 36 | no_ssu |
| bin_606 | Bacteria | Proteobacteria | Alphaproteobacteria | 86.13 | 1.94 | 3676678 | 664 | Bacterium enrichment culture clone B30(2011) 16S ribosomal RNA gene, partial sequence |
| bin_607 | Bacteria | Actinobacteria | Actinobacteria | 91.68 | 6.84 | 2334629 | 124 | no_ssu |
| bin_608 | Bacteria | Bacteroidetes | Flavobacteriia | 90.32 | 10.43 | 1621254 | 245 | no_ssu |
| bin_609 | Bacteria | Proteobacteria | Gammaproteobacteria | 2.39 | 0 | 7451225 | 2676 | Uncultured bacterium G6Clone122 16S ribosomal RNA gene, partial sequence |
| bin_61 | Bacteria | Bacteroidetes | Flavobacteriia | 4.17 | 0 | 407659 | 154 | no_ssu |
| bin_610 | Bacteria | Proteobacteria | Alphaproteobacteria | 74.21 | 2.99 | 2545798 | 553 | Uncultured bacterium clone 2C228530 16S ribosomal RNA gene, partial sequence |
| bin_611 | Bacteria | Proteobacteria | Betaproteobacteria | 18.97 | 0 | 691046 | 220 | no_ssu |
| bin_612 | Bacteria | Proteobacteria | Alphaproteobacteria | 0 | 0 | 765088 | 262 | no_ssu |
| bin_613 | Bacteria | Actinobacteria | Actinobacteria | 69.3 | 0 | 993626 | 157 | no_ssu |
| bin_614 | Bacteria | Proteobacteria | Gammaproteobacteria | 39.37 | 7.39 | 2266407 | 732 | no_ssu |
| bin_615 | Bacteria | Proteobacteria | Alphaproteobacteria | 55.62 | 1.1 | 1206235 | 383 | no_ssu |
| bin_616 | Bacteria | Proteobacteria | Alphaproteobacteria | 39.9 | 1.01 | 1153108 | 369 | no_ssu |
| bin_617 | Bacteria | Cyanobacteria | NA | 65.23 | 1.62 | 2490276 | 700 | Uncultured Archaeosporales clone CL0183 small subunit ribosomal RNA gene, partial sequence; internal transcribed spacer 1, 5.8S ribosomal RNA gene, and internal transcribed spacer 2, complete sequence; and large subunit ribosomal RNA gene, partial sequence |
| bin_618 | Bacteria | Firmicutes | Clostridia | 0 | 0 | 535075 | 115 | no_ssu |
| bin_619 | Bacteria | Proteobacteria | Gammaproteobacteria | 93.33 | 2.13 | 3232018 | 233 | Uncultured bacterium gene for 16S ribosomal RNA, partial sequence, clone: TU-31-E |
| bin_62 | Bacteria | Proteobacteria | Gammaproteobacteria | 64.97 | 0.09 | 1276135 | 323 | no_ssu |
| bin_620 | Bacteria | Proteobacteria | Betaproteobacteria | 3.45 | 0 | 221604 | 90 | no_ssu |
| bin_621 | Bacteria | Proteobacteria | Oligoflexia | 21.47 | 1.72 | 1237078 | 431 | no_ssu |
| bin_622 | Bacteria | Firmicutes | Clostridia | 0 | 0 | 408756 | 69 | no_ssu |
| bin_623 | Bacteria | Proteobacteria | Betaproteobacteria | 50.16 | 5.17 | 3262702 | 898 | Burkholderia contaminans strain SK875 chromosome SK875-3, complete sequence |
| bin_624 | Bacteria | Proteobacteria | Betaproteobacteria | 0 | 0 | 397393 | 69 | no_ssu |
| bin_625 | Bacteria | Verrucomicrobia | Opitutae | 89.02 | 5.85 | 3243890 | 508 | Opitutus terrae PB90-1, complete genome |
| bin_626 | Bacteria | Chloroflexi | Caldilineae | 72.89 | 2 | 3811657 | 879 | Roseiflexus castenholzii DSM 13941, complete genome |
| bin_627 | Bacteria | Proteobacteria | Oligoflexia | 63.58 | 14.58 | 2689356 | 774 | Uncultured bacterium clone b17 16S ribosomal RNA gene, partial sequence |
| bin_628 | Bacteria | Proteobacteria | Betaproteobacteria | 4.17 | 4.17 | 288491 | 74 | no_ssu |
| bin_629 | Bacteria | Bacteroidetes | Flavobacteriia | 90.95 | 0.9 | 1482884 | 79 | Uncultured marine microorganism clone NB062806_204 16S ribosomal RNA gene, partial sequence |
| bin_63 | Bacteria | Proteobacteria | Alphaproteobacteria | 33.2 | 0.3 | 911905 | 237 | no_ssu |
| bin_630 | Bacteria | Firmicutes | Bacilli | 1.65 | 0 | 240142 | 55 | no_ssu |
| bin_631 | Bacteria | Bacteroidetes | Cytophagia | 93.41 | 1.79 | 3429590 | 414 | no_ssu |
| bin_632 | Bacteria | Proteobacteria | Alphaproteobacteria | 0 | 0 | 218326 | 33 | no_ssu |
| bin_633 | Bacteria | Firmicutes | Bacilli | 19.87 | 4.06 | 16098238 | 5255 | Uncultured organism clone 051011_T2S1_W_T_SDP_111 small subunit ribosomal RNA gene, partial sequence; mitochondrial |
| bin_634 | Bacteria | Proteobacteria | Alphaproteobacteria | 23.96 | 2.83 | 495242 | 114 | no_ssu |
| bin_635 | Bacteria | Proteobacteria | Gammaproteobacteria | 97.75 | 0 | 6044890 | 224 | Uncultured bacterium clone F1012 16S ribosomal RNA gene, partial sequence |
| bin_636 | Bacteria | Proteobacteria | Betaproteobacteria | 73.85 | 13.65 | 1477320 | 372 | Uncultured bacterium clone CR11E 16S ribosomal RNA gene, partial sequence |
| bin_637 | Bacteria | Proteobacteria | Alphaproteobacteria | 92.47 | 3.86 | 3683927 | 563 | no_ssu |
| bin_638 | Bacteria | Proteobacteria | Alphaproteobacteria | 25.48 | 0.19 | 1058401 | 360 | no_ssu |
| bin_639 | Bacteria | Proteobacteria | Gammaproteobacteria | 86.34 | 7.44 | 2834705 | 512 | no_ssu |
| bin_64 | Bacteria | Proteobacteria | Alphaproteobacteria | 2.22 | 0.22 | 232718 | 67 | no_ssu |
| bin_640 | Bacteria | Proteobacteria | Gammaproteobacteria | 82.5 | 31.47 | 3180898 | 416 | Uncultured bacterium clone ASS_B1 16S ribosomal RNA gene and 16S-23S ribosomal RNA intergenic spacer, partial sequence |
| bin_641 | Bacteria | Proteobacteria | Betaproteobacteria | 38.5 | 0.93 | 1242811 | 395 | no_ssu |
| bin_642 | Bacteria | Proteobacteria | Alphaproteobacteria | 89.22 | 5.65 | 4026353 | 506 | no_ssu |
| bin_643 | Bacteria | Bacteroidetes | Flavobacteriia | 82.92 | 0 | 1481121 | 180 | no_ssu |
| bin_644 | Bacteria | Cyanobacteria | NA | 0.36 | 0.29 | 297507 | 66 | no_ssu |
| bin_645 | Bacteria | Proteobacteria | Alphaproteobacteria | 80.21 | 4.13 | 2551203 | 542 | no_ssu |
| bin_646 | Bacteria | Proteobacteria | Betaproteobacteria | 6.31 | 2.62 | 922407 | 267 | no_ssu |
| bin_647 | Bacteria | Proteobacteria | Betaproteobacteria | 65.12 | 19.28 | 1866886 | 483 | no_ssu |
| bin_648 | Bacteria | Proteobacteria | Gammaproteobacteria | 77.89 | 8.83 | 2579488 | 492 | Uncultured bacterium clone A16 16S ribosomal RNA gene, partial sequence |
| bin_649 | Bacteria | Actinobacteria | Actinobacteria | 20.36 | 3.51 | 540883 | 151 | no_ssu |
| bin_65 | Bacteria | Firmicutes | Clostridia | 4.3 | 0.36 | 333263 | 76 | no_ssu |
| bin_650 | Bacteria | Proteobacteria | Alphaproteobacteria | 8.54 | 0 | 978693 | 375 | no_ssu |
| bin_651 | Bacteria | Proteobacteria | Gammaproteobacteria | 32.15 | 16.03 | 1324889 | 386 | Moritella marina ATCC 15381 strain MP-1 chromosome, complete genome |
| bin_652 | Bacteria | Proteobacteria | Gammaproteobacteria | 33.93 | 0 | 1023093 | 261 | Uncultured bacterium isolate DGGE gel band RB3-56 16S ribosomal RNA gene, partial sequence |
| bin_653 | Bacteria | Proteobacteria | Alphaproteobacteria | 49.88 | 200.87 | 4545782 | 1447 | no_ssu |
| bin_654 | Bacteria | Proteobacteria | Gammaproteobacteria | 1.08 | 0 | 402506 | 119 | no_ssu |
| bin_655 | Bacteria | Proteobacteria | Betaproteobacteria | 67.51 | 1.96 | 3961646 | 888 | no_ssu |
| bin_656 | Bacteria | Proteobacteria | Gammaproteobacteria | 86.7 | 2.56 | 2092682 | 297 | no_ssu |
| bin_657 | Bacteria | Proteobacteria | Gammaproteobacteria | 89.9 | 15.37 | 2626070 | 177 | Uncultured bacterium clone C6 16S ribosomal RNA gene, partial sequence |
| bin_658 | Bacteria | Proteobacteria | Alphaproteobacteria | 28.5 | 0 | 614074 | 164 | no_ssu |
| bin_659 | Bacteria | Proteobacteria | Alphaproteobacteria | 100 | 2.04 | 2223277 | 120 | Uncultured marine bacterium clone S17-55 16S ribosomal RNA gene, partial sequence |
| bin_66 | Bacteria | Firmicutes | Bacilli | 0 | 0 | 227327 | 44 | no_ssu |
| bin_660 | Bacteria | Cyanobacteria | NA | 43.1 | 10.34 | 1974511 | 608 | Uncultured bacterium clone SHWH_night1_16S_549 16S ribosomal RNA gene, partial sequence |
| bin_661 | Bacteria | Actinobacteria | Actinobacteria | 29.97 | 0 | 436051 | 128 | no_ssu |
| bin_662 | Bacteria | Actinobacteria | Actinobacteria | 80.31 | 2.75 | 1856415 | 309 | Uncultured marine bacterium clone S17-152 16S ribosomal RNA gene, partial sequence |
| bin_663 | Bacteria | Cyanobacteria | NA | 45.76 | 3.47 | 1658317 | 552 | Uncultured bacterium clone S72 16S ribosomal RNA gene, partial sequence |
| bin_664 | Bacteria | Proteobacteria | Gammaproteobacteria | 88.45 | 1.09 | 2172291 | 198 | no_ssu |
| bin_665 | Bacteria | Proteobacteria | Betaproteobacteria | 67.01 | 35.34 | 5005286 | 1284 | no_ssu |
| bin_666 | Bacteria | Proteobacteria | Gammaproteobacteria | 51.5 | 10.34 | 1046738 | 318 | no_ssu |
| bin_667 | Bacteria | Proteobacteria | Alphaproteobacteria | 41 | 1.32 | 2547364 | 850 | no_ssu |
| bin_668 | Bacteria | Proteobacteria | Alphaproteobacteria | 50.44 | 3.04 | 1442108 | 349 | Novosphingobium pentaromativorans US6-1, complete genome |
| bin_669 | Bacteria | Bacteroidetes | Flavobacteriia | 88.73 | 13.24 | 3925834 | 703 | no_ssu |
| bin_67 | Bacteria | Actinobacteria | Actinobacteria | 70.44 | 16.86 | 1818231 | 456 | no_ssu |
| bin_670 | Bacteria | Proteobacteria | Gammaproteobacteria | 95.52 | 22.81 | 2234172 | 296 | no_ssu |
| bin_671 | Bacteria | Proteobacteria | Gammaproteobacteria | 79.84 | 2.95 | 3621584 | 805 | no_ssu |
| bin_672 | Bacteria | Cyanobacteria | NA | 53.45 | 1.72 | 2017291 | 329 | no_ssu |
| bin_673 | Bacteria | Proteobacteria | Gammaproteobacteria | 0 | 0 | 860642 | 216 | no_ssu |
| bin_674 | Bacteria | Proteobacteria | Alphaproteobacteria | 31.15 | 14.75 | 596454 | 178 | no_ssu |
| bin_675 | Bacteria | Proteobacteria | Gammaproteobacteria | 51.33 | 13.79 | 2924024 | 771 | no_ssu |
| bin_676 | Bacteria | Cyanobacteria | NA | 21.52 | 0 | 1000477 | 324 | no_ssu |
| bin_677 | Bacteria | Bacteroidetes | Flavobacteriia | 76.76 | 13.28 | 2595064 | 630 | Uncultured Bacteroidetes bacterium clone WA0.2-0d-61 16S ribosomal RNA gene, partial sequence |
| bin_678 | Bacteria | Proteobacteria | Alphaproteobacteria | 14.66 | 0 | 622682 | 215 | no_ssu |
| bin_679 | Bacteria | Proteobacteria | Gammaproteobacteria | 22.1 | 0 | 284013 | 95 | no_ssu |
| bin_68 | Bacteria | Proteobacteria | Gammaproteobacteria | 0.38 | 0 | 350208 | 135 | no_ssu |
| bin_680 | Bacteria | Actinobacteria | Actinobacteria | 58.03 | 17.87 | 1517871 | 461 | no_ssu |
| bin_681 | Bacteria | Proteobacteria | Gammaproteobacteria | 81.27 | 35.77 | 3560996 | 776 | no_ssu |
| bin_682 | Bacteria | Proteobacteria | Betaproteobacteria | 76.37 | 26.3 | 1186485 | 250 | no_ssu |
| bin_683 | Bacteria | Proteobacteria | Alphaproteobacteria | 85.29 | 1.86 | 3748511 | 676 | Sulfitobacter sp. D7 chromosome, complete genome |
| bin_684 | Bacteria | Proteobacteria | Alphaproteobacteria | 58.99 | 2.75 | 1274854 | 311 | no_ssu |
| bin_685 | Bacteria | Actinobacteria | Actinobacteria | 52.76 | 15.71 | 1101868 | 328 | no_ssu |
| bin_686 | Bacteria | Proteobacteria | Gammaproteobacteria | 3.04 | 0.43 | 424244 | 65 | no_ssu |
| bin_687 | Bacteria | Proteobacteria | Betaproteobacteria | 24.83 | 0.76 | 570412 | 168 | no_ssu |
| bin_688 | Bacteria | Actinobacteria | Actinobacteria | 65.86 | 4.47 | 985610 | 164 | no_ssu |
| bin_689 | Bacteria | Proteobacteria | Alphaproteobacteria | 17.89 | 1.75 | 1419331 | 366 | no_ssu |
| bin_69 | Bacteria | Proteobacteria | Alphaproteobacteria | 62.03 | 56.68 | 2069975 | 652 | no_ssu |
| bin_690 | Bacteria | Proteobacteria | Alphaproteobacteria | 21.47 | 0 | 930108 | 261 | no_ssu |
| bin_691 | Bacteria | Proteobacteria | Gammaproteobacteria | 72.02 | 0.84 | 2054972 | 516 | Aquitalea sp. THG-DN7.12 chromosome, complete genome |
| bin_692 | Bacteria | Proteobacteria | Gammaproteobacteria | 69.18 | 20.6 | 1940185 | 569 | Uncultured euryarchaeote clone 9NSWArch_81 16S ribosomal RNA gene, partial sequence |
| bin_693 | Bacteria | Proteobacteria | Gammaproteobacteria | 80.91 | 1.82 | 4012117 | 663 | no_ssu |
| bin_694 | Bacteria | Proteobacteria | Betaproteobacteria | 0 | 0 | 347718 | 83 | no_ssu |
| bin_695 | Bacteria | Cyanobacteria | NA | 54.47 | 11.21 | 2324820 | 495 | no_ssu |
| bin_696 | Bacteria | Proteobacteria | Gammaproteobacteria | 54.49 | 14.01 | 2115093 | 680 | no_ssu |
| bin_697 | Bacteria | Proteobacteria | Betaproteobacteria | 85.06 | 3.69 | 2619671 | 583 | Uncultured bacterium clone 3C002581 16S ribosomal RNA gene, partial sequence |
| bin_698 | Bacteria | Actinobacteria | Actinobacteria | 51.21 | 6.62 | 2459899 | 668 | no_ssu |
| bin_699 | Bacteria | Proteobacteria | Gammaproteobacteria | 17.55 | 1.72 | 608196 | 195 | no_ssu |
| bin_7 | Bacteria | Proteobacteria | Alphaproteobacteria | 86.45 | 2.49 | 3174692 | 540 | Uncultured bacterium clone CE1-DCM-79 16S ribosomal RNA gene, partial sequence |
| bin_70 | Bacteria | Actinobacteria | Actinobacteria | 73.5 | 4.66 | 1332349 | 328 | no_ssu |
| bin_700 | Bacteria | Proteobacteria | Gammaproteobacteria | 22.41 | 0 | 848260 | 283 | Uncultured sediment bacterium DNA, 16S ribosomal RNA and intergenic spacer, clone: OB8 |
| bin_701 | Bacteria | Proteobacteria | Alphaproteobacteria | 77.87 | 4.02 | 3543039 | 736 | no_ssu |
| bin_702 | Bacteria | Bacteroidetes | Flavobacteriia | 82.54 | 3.78 | 2211504 | 445 | no_ssu |
| bin_703 | Bacteria | Cyanobacteria | NA | 49.66 | 0 | 1037902 | 319 | no_ssu |
| bin_704 | Bacteria | Bacteroidetes | Flavobacteriia | 39.81 | 7.76 | 1455467 | 392 | Uncultured marine bacterium clone S1-18 16S ribosomal RNA gene, partial sequence |
| bin_705 | Bacteria | Firmicutes | Bacilli | 0 | 0 | 981503 | 58 | no_ssu |
| bin_706 | Bacteria | Firmicutes | Bacilli | 3.47 | 0 | 567993 | 175 | no_ssu |
| bin_707 | Bacteria | Proteobacteria | Gammaproteobacteria | 5.38 | 0.58 | 354157 | 85 | no_ssu |
| bin_708 | Bacteria | Proteobacteria | Gammaproteobacteria | 94.07 | 0.74 | 1714489 | 51 | Uncultured bacterium clone PC-FL10-68 16S ribosomal RNA gene, partial sequence |
| bin_709 | Bacteria | Cyanobacteria | NA | 42.64 | 7.93 | 2544533 | 716 | Synechococcus sp. WH 8101 chromosome, complete genome |
| bin_71 | Bacteria | Proteobacteria | Gammaproteobacteria | 0 | 0 | 327563 | 20 | no_ssu |
| bin_710 | Bacteria | Cyanobacteria | NA | 60.58 | 9.74 | 2053215 | 613 | no_ssu |
| bin_711 | Bacteria | Proteobacteria | Alphaproteobacteria | 62.67 | 9.39 | 2346894 | 710 | no_ssu |
| bin_712 | Bacteria | Firmicutes | Clostridia | 4.37 | 0.5 | 338265 | 46 | no_ssu |
| bin_713 | Bacteria | Bacteroidetes | Flavobacteriia | 51.08 | 2.54 | 786937 | 245 | no_ssu |
| bin_714 | Bacteria | Proteobacteria | Betaproteobacteria | 60.34 | 32.76 | 1585657 | 361 | Candidatus Methylopumilus universalis strain MMS-VI-38 chromosome |
| bin_715 | Bacteria | Proteobacteria | Alphaproteobacteria | 86.92 | 7.13 | 2049835 | 382 | no_ssu |
| bin_716 | Bacteria | Actinobacteria | Actinobacteria | 39.83 | 3.45 | 2044873 | 619 | Uncultured bacterium clone OTU7382 16S ribosomal RNA gene, partial sequence |
| bin_717 | Bacteria | Proteobacteria | Gammaproteobacteria | 5.49 | 0 | 558391 | 208 | no_ssu |
| bin_718 | Bacteria | Actinobacteria | Actinobacteria | 53.65 | 19.9 | 1644716 | 511 | Uncultured bacterium clone 4 16S ribosomal RNA gene, partial sequence |
| bin_719 | Bacteria | Proteobacteria | Gammaproteobacteria | 42.11 | 3.51 | 1799059 | 514 | no_ssu |
| bin_72 | Bacteria | Proteobacteria | Betaproteobacteria | 56.35 | 48.43 | 7257814 | 2614 | no_ssu |
| bin_720 | Bacteria | Proteobacteria | Alphaproteobacteria | 10.7 | 1.01 | 4384177 | 1651 | no_ssu |
| bin_721 | Bacteria | Tenericutes | Mollicutes | 4.55 | 0.5 | 414406 | 36 | no_ssu |
| bin_722 | Bacteria | Proteobacteria | Gammaproteobacteria | 21.13 | 1.08 | 867826 | 322 | Uncultured bacterium clone WAS043 16S ribosomal RNA gene, partial sequence |
| bin_723 | Bacteria | Proteobacteria | Betaproteobacteria | 28.23 | 1.28 | 1353875 | 455 | no_ssu |
| bin_724 | Archaea | Thaumarchaeota | NA | 0 | 0 | 2677762 | 580 | no_ssu |
| bin_725 | Bacteria | Proteobacteria | Gammaproteobacteria | 4.17 | 0 | 544463 | 186 | no_ssu |
| bin_726 | Bacteria | Proteobacteria | Gammaproteobacteria | 30.17 | 0 | 1216728 | 417 | no_ssu |
| bin_727 | Bacteria | Proteobacteria | Gammaproteobacteria | 36.02 | 10.27 | 9195008 | 2945 | Minutocellus polymorphus gene for 18S ribosomal RNA, partial sequence, strain: NIES-3970 |
| bin_728 | Bacteria | Proteobacteria | Gammaproteobacteria | 10.78 | 0.29 | 3385918 | 1209 | no_ssu |
| bin_729 | Bacteria | Actinobacteria | Actinobacteria | 0 | 0 | 4382194 | 1091 | no_ssu |
| bin_73 | Bacteria | Proteobacteria | Betaproteobacteria | 0 | 0 | 316337 | 35 | no_ssu |
| bin_730 | Bacteria | Proteobacteria | Alphaproteobacteria | 62.62 | 0.87 | 1150586 | 282 | no_ssu |
| bin_731 | Bacteria | Bacteroidetes | Flavobacteriia | 0 | 0 | 276445 | 61 | no_ssu |
| bin_732 | Bacteria | Actinobacteria | NA | 10.84 | 0 | 1158394 | 265 | no_ssu |
| bin_733 | Viruses | NA | NA | 0 | 0 | 1567073 | 220 | no_ssu |
| bin_734 | Bacteria | Firmicutes | Bacilli | 5.31 | 0 | 447862 | 54 | no_ssu |
| bin_735 | Bacteria | Actinobacteria | Actinobacteria | 37.41 | 18.42 | 2660030 | 865 | no_ssu |
| bin_736 | Bacteria | Proteobacteria | Epsilonproteobacteria | 67.14 | 4.31 | 1682362 | 432 | no_ssu |
| bin_737 | Bacteria | Proteobacteria | Alphaproteobacteria | 94.51 | 1.78 | 1371410 | 103 | Uncultured bacterium clone MH7m-91 16S ribosomal RNA gene, partial sequence |
| bin_738 | Bacteria | Firmicutes | Bacilli | 0 | 0 | 364699 | 90 | no_ssu |
| bin_739 | Bacteria | Proteobacteria | Betaproteobacteria | 30.28 | 0 | 880510 | 225 | no_ssu |
| bin_74 | Bacteria | Cyanobacteria | NA | 88.63 | 2.63 | 3612913 | 620 | no_ssu |
| bin_740 | Bacteria | Bacteroidetes | Flavobacteriia | 87.29 | 0.54 | 2154100 | 319 | no_ssu |
| bin_741 | Bacteria | Proteobacteria | Gammaproteobacteria | 83.36 | 6.08 | 3342754 | 513 | Uncultured bacterium clone 72-02 16S ribosomal RNA gene, partial sequence |
| bin_742 | Bacteria | Bacteroidetes | Flavobacteriia | 33.26 | 0 | 466817 | 122 | no_ssu |
| bin_743 | Bacteria | Proteobacteria | Alphaproteobacteria | 18.68 | 0 | 221954 | 52 | no_ssu |
| bin_744 | Bacteria | Proteobacteria | Gammaproteobacteria | 71.99 | 2.8 | 2414473 | 322 | Uncultured delta proteobacterium partial 16S rRNA gene, clone ZS-4-53 |
| bin_745 | Bacteria | Cyanobacteria | NA | 19.8 | 0 | 696093 | 239 | no_ssu |
| bin_746 | Bacteria | Proteobacteria | Alphaproteobacteria | 5.79 | 0.47 | 860507 | 125 | no_ssu |
| bin_747 | Bacteria | Bacteroidetes | Flavobacteriia | 88.53 | 9.04 | 2484044 | 381 | no_ssu |
| bin_748 | Bacteria | Proteobacteria | Alphaproteobacteria | 90.78 | 2.28 | 5036666 | 497 | Akkermansia muciniphila strain DSM 22959 chromosome, complete genome |
| bin_749 | Bacteria | Proteobacteria | Alphaproteobacteria | 87.7 | 29.98 | 2734729 | 690 | Uncultured alpha proteobacterium HF0070_34A12 genomic sequence |
| bin_75 | Bacteria | Proteobacteria | Alphaproteobacteria | 15.05 | 1.72 | 1221910 | 403 | no_ssu |
| bin_750 | Viruses | NA | NA | 0 | 0 | 210446 | 45 | no_ssu |
| bin_751 | Archaea | Thaumarchaeota | NA | 84.95 | 3.88 | 1004306 | 179 | no_ssu |
| bin_752 | Bacteria | Proteobacteria | Gammaproteobacteria | 70.23 | 2.35 | 4117137 | 920 | Uncultured bacterium clone T61 16S ribosomal RNA gene, partial sequence |
| bin_753 | Bacteria | Proteobacteria | Alphaproteobacteria | 61.86 | 7.02 | 2953526 | 684 | Uncultured bacterium clone Contig\41 16S ribosomal RNA gene, partial sequence |
| bin_754 | Bacteria | Proteobacteria | Alphaproteobacteria | 100 | 105 | 8038780 | 1830 | Uncultured bacterium 92 genomic sequence |
| bin_755 | Bacteria | Proteobacteria | Alphaproteobacteria | 82.26 | 13.99 | 2565377 | 584 | no_ssu |
| bin_756 | Bacteria | Actinobacteria | Actinobacteria | 88.43 | 5.3 | 2422690 | 392 | Uncultured bacterium clone C38 16S ribosomal RNA gene, partial sequence |
| bin_757 | Bacteria | Proteobacteria | Gammaproteobacteria | 3.09 | 0 | 515260 | 119 | no_ssu |
| bin_758 | Bacteria | Actinobacteria | Actinobacteria | 54.07 | 1.49 | 1770548 | 481 | Uncultured bacterium clone 10 16S ribosomal RNA gene, partial sequence |
| bin_759 | Bacteria | Firmicutes | Bacilli | 0 | 0 | 254172 | 54 | no_ssu |
| bin_76 | Bacteria | Bacteroidetes | Flavobacteriia | 16.9 | 0 | 309339 | 107 | no_ssu |
| bin_760 | Bacteria | Actinobacteria | Actinobacteria | 88.7 | 4.73 | 2227633 | 255 | no_ssu |
| bin_761 | Bacteria | Firmicutes | Bacilli | 60.12 | 1.41 | 872963 | 178 | no_ssu |
| bin_762 | Bacteria | Bacteroidetes | Flavobacteriia | 0 | 0 | 405543 | 46 | no_ssu |
| bin_763 | Bacteria | Proteobacteria | Gammaproteobacteria | 76.05 | 4.74 | 1035890 | 50 | Salmonella enterica subsp. enterica serovar Typhimurium strain 22792, complete genome |
| bin_764 | Bacteria | Bacteroidetes | Flavobacteriia | 82.12 | 8 | 1799252 | 276 | Bathycoccus prasinos genomic : Mitochondrion |
| bin_765 | Bacteria | Actinobacteria | Actinobacteria | 80.82 | 6.06 | 2121332 | 411 | no_ssu |
| bin_766 | Bacteria | Actinobacteria | Actinobacteria | 33.5 | 4.19 | 5400035 | 1864 | no_ssu |
| bin_767 | Bacteria | Proteobacteria | Alphaproteobacteria | 67.05 | 8.32 | 2433121 | 710 | Uncultured verrucomicrobium HF0070_15G23 genomic sequence |
| bin_768 | Bacteria | Actinobacteria | Actinobacteria | 62.03 | 24.45 | 938981 | 277 | no_ssu |
| bin_769 | Bacteria | Firmicutes | Bacilli | 0 | 0 | 310703 | 33 | no_ssu |
| bin_77 | Bacteria | Actinobacteria | Actinobacteria | 55.9 | 4.87 | 799783 | 152 | no_ssu |
| bin_770 | Bacteria | Proteobacteria | Gammaproteobacteria | 27.91 | 1.83 | 696220 | 243 | no_ssu |
| bin_771 | Bacteria | Proteobacteria | Alphaproteobacteria | 60.12 | 1.62 | 1569756 | 374 | no_ssu |
| bin_772 | Bacteria | Proteobacteria | Gammaproteobacteria | 33.07 | 3.45 | 1352480 | 444 | no_ssu |
| bin_773 | Bacteria | Proteobacteria | Alphaproteobacteria | 90.04 | 21.33 | 3083652 | 612 | Uncultured bacterium clone WC3_64 16S ribosomal RNA gene, partial sequence |
| bin_774 | Bacteria | Bacteroidetes | Flavobacteriia | 72.49 | 39.56 | 3830627 | 1253 | no_ssu |
| bin_775 | Bacteria | Proteobacteria | Alphaproteobacteria | 76.62 | 46.63 | 11312874 | 3172 | no_ssu |
| bin_776 | Bacteria | Actinobacteria | Actinobacteria | 36.11 | 5.17 | 639835 | 209 | no_ssu |
| bin_777 | Bacteria | Proteobacteria | Gammaproteobacteria | 90.69 | 3.58 | 4022181 | 721 | no_ssu |
| bin_778 | Bacteria | Proteobacteria | Alphaproteobacteria | 97.44 | 6.72 | 3122569 | 205 | Uncultured bacterium clone VE07-62-BAC 16S ribosomal RNA gene, partial sequence |
| bin_779 | Bacteria | Proteobacteria | Betaproteobacteria | 3.51 | 0 | 309777 | 33 | no_ssu |
| bin_78 | Bacteria | Proteobacteria | Gammaproteobacteria | 31.03 | 0 | 1062186 | 321 | no_ssu |
| bin_780 | Bacteria | Proteobacteria | Gammaproteobacteria | 86.17 | 4.77 | 4846942 | 916 | no_ssu |
| bin_781 | NA | NA | NA | 4.45 | 0.5 | 323733 | 11 | no_ssu |
| bin_782 | Bacteria | Actinobacteria | Actinobacteria | 74.71 | 8.92 | 1212029 | 264 | Kocuria rosea strain ATCC 186 chromosome, complete genome |
| bin_783 | Bacteria | Proteobacteria | Alphaproteobacteria | 0 | 0 | 202940 | 64 | no_ssu |
| bin_784 | Bacteria | Firmicutes | Clostridia | 17.32 | 0 | 704407 | 227 | no_ssu |
| bin_785 | Bacteria | Firmicutes | Bacilli | 73.35 | 41.62 | 1784712 | 508 | Uncultured bacterium clone AN0C1AB08 16S ribosomal RNA gene, partial sequence |
| bin_786 | Bacteria | Proteobacteria | Betaproteobacteria | 8.78 | 0 | 574567 | 203 | no_ssu |
| bin_787 | Bacteria | Actinobacteria | Actinobacteria | 90.6 | 2.14 | 2337596 | 200 | Actinobacteria bacterium IMCC26256, complete genome |
| bin_788 | Bacteria | Proteobacteria | Gammaproteobacteria | 8.76 | 0 | 1953595 | 698 | no_ssu |
| bin_789 | Bacteria | Proteobacteria | Gammaproteobacteria | 91.61 | 59.08 | 4227646 | 974 | Uncultured bacterium clone 4I.7E 16S ribosomal RNA gene, partial sequence |
| bin_79 | Bacteria | Proteobacteria | Alphaproteobacteria | 2.43 | 0.16 | 564668 | 179 | Uncultured alpha proteobacterium clone A8W_212 16S ribosomal RNA gene, partial sequence |
| bin_790 | Bacteria | Proteobacteria | Alphaproteobacteria | 96.39 | 3.61 | 5794353 | 636 | Uncultured bacterium clone 5C230780 16S ribosomal RNA gene, partial sequence |
| bin_791 | Bacteria | Verrucomicrobia | Opitutae | 73.88 | 2.32 | 3008482 | 750 | Uncultured bacterium clone NC6F1d1_17434 16S ribosomal RNA gene, partial sequence |
| bin_792 | Bacteria | Proteobacteria | Betaproteobacteria | 84.33 | 17.24 | 3302387 | 458 | no_ssu |
| bin_793 | Bacteria | Proteobacteria | Betaproteobacteria | 44.75 | 12.07 | 957295 | 164 | no_ssu |
| bin_794 | Bacteria | Bacteroidetes | Flavobacteriia | 97.93 | 1.11 | 2687404 | 168 | no_ssu |
| bin_795 | Bacteria | Actinobacteria | NA | 51.9 | 2.59 | 743223 | 214 | no_ssu |
| bin_796 | Bacteria | Proteobacteria | Gammaproteobacteria | 0 | 0 | 379021 | 23 | no_ssu |
| bin_797 | Bacteria | Proteobacteria | Alphaproteobacteria | 31.19 | 0 | 1234052 | 427 | no_ssu |
| bin_798 | Bacteria | Firmicutes | Bacilli | 0 | 0 | 2606598 | 635 | no_ssu |
| bin_799 | Bacteria | Actinobacteria | Actinobacteria | 71.55 | 1.72 | 1041643 | 196 | no_ssu |
| bin_8 | Bacteria | Proteobacteria | Gammaproteobacteria | 0 | 0 | 229658 | 38 | no_ssu |
| bin_80 | Bacteria | Proteobacteria | Alphaproteobacteria | 43.1 | 0 | 930521 | 295 | no_ssu |
| bin_800 | Bacteria | Firmicutes | Bacilli | 6.78 | 0.29 | 1096027 | 303 | no_ssu |
| bin_801 | Bacteria | Proteobacteria | Gammaproteobacteria | 6.11 | 17.24 | 1028587 | 245 | no_ssu |
| bin_802 | Bacteria | Proteobacteria | Gammaproteobacteria | 73.43 | 0 | 4441590 | 951 | no_ssu |
| bin_803 | Bacteria | Proteobacteria | Gammaproteobacteria | 94.82 | 0.99 | 2543291 | 177 | Alistipes shahii WAL 8301 draft genome |
| bin_804 | Bacteria | Bacteroidetes | Flavobacteriia | 79.15 | 7.35 | 2011351 | 522 | Hemiselmis andersenii strain CCMP 644 mitochondrion, complete genome |
| bin_805 | Bacteria | Proteobacteria | Gammaproteobacteria | 91.1 | 1.16 | 2260384 | 98 | Simiduia agarivorans SA1 = DSM 21679, complete genome |
| bin_806 | Bacteria | Firmicutes | Bacilli | 3.37 | 0 | 370154 | 89 | no_ssu |
| bin_807 | Bacteria | Proteobacteria | Alphaproteobacteria | 69.44 | 0.84 | 3124373 | 284 | no_ssu |
| bin_808 | Bacteria | Cyanobacteria | NA | 41.38 | 0 | 1316096 | 350 | Synechococcus sp. CB0101 chromosome, complete genome |
| bin_809 | Bacteria | Proteobacteria | Alphaproteobacteria | 24.83 | 0 | 980809 | 345 | no_ssu |
| bin_81 | Bacteria | Proteobacteria | Gammaproteobacteria | 61.39 | 3.96 | 594367 | 86 | Uncultured candidate division WS5 bacterium clone YL018 16S ribosomal RNA gene, partial sequence |
| bin_810 | Bacteria | Proteobacteria | Alphaproteobacteria | 96.53 | 3.89 | 2461056 | 261 | Uncultured bacterium clone MD79 16S ribosomal RNA gene, partial sequence |
| bin_811 | Bacteria | Firmicutes | Bacilli | 63.52 | 3.32 | 1005147 | 278 | Gracilimonas sp. 8A47 16S ribosomal RNA gene, partial sequence |
| bin_812 | Bacteria | Proteobacteria | Gammaproteobacteria | 92.88 | 0.56 | 2581018 | 251 | no_ssu |
| bin_813 | Bacteria | Firmicutes | Clostridia | 3.01 | 0 | 218620 | 61 | no_ssu |
| bin_814 | Bacteria | Proteobacteria | Betaproteobacteria | 59.84 | 2.83 | 2092600 | 417 | no_ssu |
| bin_815 | Bacteria | Proteobacteria | Alphaproteobacteria | 24.61 | 2.3 | 1260291 | 428 | no_ssu |
| bin_816 | Bacteria | Proteobacteria | Gammaproteobacteria | 47.81 | 0 | 808192 | 267 | no_ssu |
| bin_817 | Bacteria | Bacteroidetes | Flavobacteriia | 69.38 | 4.91 | 2107856 | 517 | no_ssu |
| bin_818 | Bacteria | Actinobacteria | Actinobacteria | 11.72 | 1.76 | 2352618 | 605 | Uncultured bacterium clone 602_TC3_89 16S ribosomal RNA gene, partial sequence |
| bin_819 | Bacteria | Proteobacteria | Alphaproteobacteria | 22.41 | 0 | 582836 | 205 | no_ssu |
| bin_82 | Bacteria | Firmicutes | Bacilli | 6.31 | 0.5 | 470558 | 16 | no_ssu |
| bin_820 | Bacteria | Proteobacteria | Gammaproteobacteria | 54.24 | 0.36 | 3110181 | 855 | no_ssu |
| bin_821 | Bacteria | Proteobacteria | Alphaproteobacteria | 8.33 | 0 | 623036 | 214 | no_ssu |
| bin_822 | Bacteria | Firmicutes | Bacilli | 23.28 | 0 | 544686 | 200 | no_ssu |
| bin_823 | Bacteria | Actinobacteria | Actinobacteria | 83.45 | 5.73 | 1875915 | 324 | Uncultured actinobacterium clone THAD2-72 16S ribosomal RNA gene, partial sequence |
| bin_824 | Bacteria | Actinobacteria | Actinobacteria | 49.26 | 1.53 | 1147315 | 271 | Uncultured bacterium clone X0022 16S ribosomal RNA gene, partial sequence |
| bin_825 | Bacteria | Proteobacteria | Gammaproteobacteria | 94.87 | 2.58 | 3366300 | 272 | Uncultured gamma proteobacterium clone S-30-24 16S ribosomal RNA gene, partial sequence |
| bin_826 | Bacteria | Proteobacteria | Gammaproteobacteria | 42.5 | 3.65 | 748434 | 187 | Uncultured bacterium clone PEX_8+P_H9 16S ribosomal RNA gene, partial sequence |
| bin_827 | Bacteria | Proteobacteria | Alphaproteobacteria | 0 | 0 | 232992 | 50 | no_ssu |
| bin_828 | Bacteria | Proteobacteria | Alphaproteobacteria | 91.46 | 5.11 | 2036820 | 208 | Uncultured bacterium clone NIT_75 16S ribosomal RNA gene, partial sequence |
| bin_829 | Bacteria | Proteobacteria | Gammaproteobacteria | 53.35 | 1.1 | 484406 | 62 | no_ssu |
| bin_83 | Bacteria | Proteobacteria | Betaproteobacteria | 5.17 | 0 | 646343 | 228 | no_ssu |
| bin_830 | Bacteria | Firmicutes | Bacilli | 3.51 | 0.5 | 355390 | 68 | no_ssu |
| bin_831 | Bacteria | Proteobacteria | Gammaproteobacteria | 3.22 | 1.37 | 323735 | 78 | no_ssu |
| bin_832 | Viruses | NA | NA | 2.11 | 0 | 593040 | 27 | no_ssu |
| bin_833 | Bacteria | Actinobacteria | Actinobacteria | 21.03 | 0 | 282781 | 56 | no_ssu |
| bin_834 | Bacteria | Proteobacteria | Betaproteobacteria | 8.33 | 0 | 444362 | 170 | no_ssu |
| bin_835 | Bacteria | Bacteroidetes | Chitinophagia | 19.07 | 0.62 | 856141 | 319 | no_ssu |
| bin_836 | Bacteria | Proteobacteria | Betaproteobacteria | 81.32 | 4.47 | 2462833 | 515 | Burkholderia multivorans strain FDAARGOS_622 chromosome 3, complete sequence |
| bin_837 | Bacteria | Proteobacteria | Betaproteobacteria | 91.6 | 2.13 | 2102137 | 245 | no_ssu |
| bin_838 | Bacteria | Actinobacteria | Actinobacteria | 23.57 | 0 | 554574 | 172 | no_ssu |
| bin_839 | Bacteria | Actinobacteria | Actinobacteria | 0 | 0 | 262725 | 70 | no_ssu |
| bin_84 | Bacteria | Firmicutes | Bacilli | 76.37 | 12.84 | 940319 | 191 | no_ssu |
| bin_840 | Bacteria | Proteobacteria | Alphaproteobacteria | 40.91 | 1.85 | 362809 | 79 | no_ssu |
| bin_841 | Bacteria | Bacteroidetes | Flavobacteriia | 4.55 | 1.58 | 302872 | 89 | no_ssu |
| bin_842 | Bacteria | Firmicutes | Bacilli | 0 | 0 | 224181 | 29 | no_ssu |
| bin_843 | Bacteria | Actinobacteria | Actinobacteria | 68.54 | 5.56 | 1106297 | 285 | no_ssu |
| bin_844 | Bacteria | Proteobacteria | Gammaproteobacteria | 0.93 | 0 | 1658597 | 513 | no_ssu |
| bin_845 | Bacteria | Firmicutes | Clostridia | 0 | 0 | 211867 | 48 | no_ssu |
| bin_846 | Bacteria | Proteobacteria | Alphaproteobacteria | 31.41 | 0 | 1019607 | 355 | Uncultured bacterium clone 5C230826 16S ribosomal RNA gene, partial sequence |
| bin_847 | Bacteria | Tenericutes | Mollicutes | 0 | 0 | 262489 | 80 | no_ssu |
| bin_848 | Bacteria | Firmicutes | Clostridia | 4.84 | 0.5 | 458609 | 62 | no_ssu |
| bin_849 | Bacteria | Proteobacteria | Alphaproteobacteria | 0 | 0 | 266987 | 104 | no_ssu |
| bin_85 | Bacteria | Proteobacteria | Gammaproteobacteria | 0 | 0 | 619628 | 158 | no_ssu |
| bin_850 | Bacteria | Proteobacteria | Gammaproteobacteria | 0 | 0 | 220864 | 58 | no_ssu |
| bin_851 | Bacteria | Proteobacteria | Alphaproteobacteria | 9.89 | 0 | 918193 | 261 | Uncultured bacterium clone W2-62 16S ribosomal RNA gene, partial sequence |
| bin_852 | Bacteria | Proteobacteria | Betaproteobacteria | 97.24 | 1.18 | 2931090 | 67 | Bacterium SH5-6 16S ribosomal RNA gene, partial sequence |
| bin_853 | Bacteria | Proteobacteria | Gammaproteobacteria | 94.12 | 0.8 | 2868963 | 101 | no_ssu |
| bin_854 | Bacteria | Proteobacteria | Gammaproteobacteria | 61.33 | 2.89 | 2064823 | 573 | no_ssu |
| bin_855 | Bacteria | Bacteroidetes | Cytophagia | 27.59 | 0 | 871785 | 258 | no_ssu |
| bin_856 | Bacteria | Proteobacteria | Betaproteobacteria | 78.73 | 40.14 | 4849380 | 1231 | no_ssu |
| bin_857 | Bacteria | Proteobacteria | Alphaproteobacteria | 71.2 | 1.31 | 3524672 | 828 | no_ssu |
| bin_858 | Bacteria | Proteobacteria | Betaproteobacteria | 49.83 | 0.62 | 1244320 | 220 | no_ssu |
| bin_859 | Bacteria | Bacteroidetes | Flavobacteriia | 42.24 | 0 | 1607503 | 391 | no_ssu |
| bin_86 | Bacteria | Firmicutes | Bacilli | 3.35 | 0 | 594390 | 155 | no_ssu |
| bin_860 | Bacteria | Proteobacteria | Gammaproteobacteria | 52.61 | 3.72 | 2580501 | 738 | no_ssu |
| bin_861 | Bacteria | Actinobacteria | Actinobacteria | 56.63 | 0.85 | 1137074 | 301 | no_ssu |
| bin_862 | Bacteria | Proteobacteria | Gammaproteobacteria | 87.83 | 1.26 | 3273498 | 218 | no_ssu |
| bin_863 | Bacteria | Proteobacteria | Alphaproteobacteria | 97.16 | 0.94 | 2264462 | 168 | no_ssu |
| bin_864 | Bacteria | Cyanobacteria | NA | 99.52 | 0.33 | 3480185 | 184 | Cylindrospermopsis raciborskii NIES-1260 gene for 16S ribosomal RNA, partial sequence |
| bin_865 | Bacteria | Proteobacteria | Betaproteobacteria | 32.84 | 0 | 1032362 | 178 | no_ssu |
| bin_866 | Bacteria | Proteobacteria | Betaproteobacteria | 94.58 | 5.54 | 6373717 | 607 | Uncultured Alcaligenaceae bacterium clone TGRWLFZ-16s-SI380 16S ribosomal RNA gene, partial sequence |
| bin_867 | Bacteria | Proteobacteria | Gammaproteobacteria | 93.89 | 2.47 | 3273651 | 113 | no_ssu |
| bin_868 | Bacteria | Proteobacteria | Gammaproteobacteria | 57.76 | 6.03 | 2984623 | 862 | no_ssu |
| bin_869 | Bacteria | Firmicutes | Bacilli | 3.04 | 0.89 | 516318 | 120 | no_ssu |
| bin_87 | Bacteria | Proteobacteria | Betaproteobacteria | 36.21 | 3.45 | 1623832 | 555 | no_ssu |
| bin_870 | Bacteria | Proteobacteria | Gammaproteobacteria | 59.09 | 0.84 | 2717870 | 696 | no_ssu |
| bin_871 | Bacteria | Actinobacteria | NA | 23.04 | 1.72 | 338172 | 112 | no_ssu |
| bin_872 | Bacteria | Proteobacteria | Gammaproteobacteria | 4.17 | 0 | 455405 | 158 | no_ssu |
| bin_873 | Bacteria | Actinobacteria | Actinobacteria | 31.21 | 3.45 | 621766 | 184 | no_ssu |
| bin_874 | Bacteria | Proteobacteria | Alphaproteobacteria | 18.1 | 0 | 397784 | 143 | no_ssu |
| bin_875 | Bacteria | Proteobacteria | Alphaproteobacteria | 43.57 | 1.72 | 735267 | 248 | no_ssu |
| bin_876 | Bacteria | Proteobacteria | Gammaproteobacteria | 6.78 | 0.5 | 492693 | 42 | no_ssu |
| bin_877 | Bacteria | Proteobacteria | Alphaproteobacteria | 0 | 0 | 248895 | 18 | no_ssu |
| bin_878 | Bacteria | Proteobacteria | Gammaproteobacteria | 76.44 | 8.26 | 1225839 | 179 | no_ssu |
| bin_879 | Bacteria | Proteobacteria | Gammaproteobacteria | 80.74 | 4.9 | 3197954 | 679 | no_ssu |
| bin_88 | Bacteria | Proteobacteria | Gammaproteobacteria | 31.03 | 0 | 851821 | 243 | no_ssu |
| bin_880 | Bacteria | Bacteroidetes | Flavobacteriia | 67.76 | 1.72 | 941697 | 112 | Uncultured marine bacterium clone S10-15 16S ribosomal RNA gene, partial sequence |
| bin_881 | Bacteria | Actinobacteria | Actinobacteria | 4.17 | 0 | 304800 | 107 | no_ssu |
| bin_882 | Bacteria | Proteobacteria | Gammaproteobacteria | 42.8 | 0.64 | 723673 | 252 | Uncultured bacterium clone BQ 16S ribosomal RNA gene, partial sequence |
| bin_883 | Bacteria | Proteobacteria | Alphaproteobacteria | 16.14 | 0 | 778958 | 271 | no_ssu |
| bin_884 | Bacteria | Proteobacteria | Alphaproteobacteria | 2.19 | 0 | 233932 | 60 | no_ssu |
| bin_885 | Bacteria | Firmicutes | Bacilli | 0 | 0 | 842565 | 200 | no_ssu |
| bin_886 | Bacteria | Proteobacteria | Alphaproteobacteria | 64.88 | 0 | 2601461 | 657 | no_ssu |
| bin_887 | Bacteria | Firmicutes | Bacilli | 1.4 | 0 | 411757 | 131 | no_ssu |
| bin_888 | Bacteria | Actinobacteria | Actinobacteria | 53.51 | 0.42 | 906049 | 237 | Uncultured marine bacterium clone S24-72 16S ribosomal RNA gene, partial sequence |
| bin_889 | Bacteria | Proteobacteria | Gammaproteobacteria | 2.39 | 0.88 | 290983 | 73 | no_ssu |
| bin_89 | Bacteria | Proteobacteria | Gammaproteobacteria | 98.69 | 3.51 | 5700016 | 156 | no_ssu |
| bin_890 | Bacteria | Proteobacteria | Gammaproteobacteria | 89.64 | 86.74 | 4706887 | 763 | no_ssu |
| bin_891 | Bacteria | Firmicutes | Clostridia | 0 | 0 | 263915 | 53 | no_ssu |
| bin_892 | Bacteria | Proteobacteria | Gammaproteobacteria | 59.77 | 0 | 1038369 | 199 | Uncultured bacterium clone T0-Ps-25C-25 16S ribosomal RNA gene, complete sequence |
| bin_893 | Bacteria | Proteobacteria | Gammaproteobacteria | 15.67 | 0 | 1195692 | 402 | no_ssu |
| bin_894 | Bacteria | Proteobacteria | Gammaproteobacteria | 68.94 | 1.53 | 2320650 | 570 | Gamma proteobacterium BW-2 chromosome, complete genome |
| bin_895 | Bacteria | Bacteroidetes | Flavobacteriia | 77.95 | 2.7 | 1981206 | 450 | no_ssu |
| bin_896 | Bacteria | Actinobacteria | Actinobacteria | 13.4 | 2.59 | 696229 | 275 | no_ssu |
| bin_897 | Bacteria | Actinobacteria | Actinobacteria | 69.72 | 3.42 | 1951355 | 434 | Uncultured Acidimicrobiales bacterium clone OTU187 16S ribosomal RNA gene, partial sequence |
| bin_898 | Bacteria | Proteobacteria | Gammaproteobacteria | 73.92 | 17.72 | 3296577 | 902 | no_ssu |
| bin_899 | Bacteria | Proteobacteria | Gammaproteobacteria | 10.54 | 1.62 | 3907491 | 1491 | no_ssu |
| bin_9 | Bacteria | Proteobacteria | Alphaproteobacteria | 100 | 645.87 | 12667669 | 3857 | Uncultured bacterium clone E136_F05 16S ribosomal RNA gene, partial sequence |
| bin_90 | Bacteria | Firmicutes | Bacilli | 0 | 0 | 339923 | 65 | no_ssu |
| bin_900 | Bacteria | Proteobacteria | Gammaproteobacteria | 19.12 | 0 | 394977 | 139 | no_ssu |
| bin_901 | Bacteria | Proteobacteria | Betaproteobacteria | 52.85 | 0 | 1192785 | 363 | no_ssu |
| bin_902 | Bacteria | Firmicutes | Negativicutes | 0 | 0 | 239634 | 40 | no_ssu |
| bin_903 | Bacteria | Proteobacteria | Gammaproteobacteria | 3.79 | 0 | 276385 | 54 | no_ssu |
| bin_904 | Bacteria | Proteobacteria | Gammaproteobacteria | 32.76 | 0 | 994964 | 314 | no_ssu |
| bin_905 | Bacteria | Verrucomicrobia | Opitutae | 97.95 | 3.42 | 2598956 | 54 | no_ssu |
| bin_906 | Viruses | NA | NA | 2.79 | 0.31 | 325749 | 90 | no_ssu |
| bin_907 | Bacteria | Proteobacteria | Betaproteobacteria | 22.41 | 3.45 | 1319515 | 431 | no_ssu |
| bin_908 | Bacteria | Proteobacteria | Alphaproteobacteria | 66.77 | 477.51 | 14618903 | 3828 | Uncultured bacterium clone FW3W7_P3_C01 16S ribosomal RNA gene, partial sequence |
| bin_909 | Bacteria | Bacteroidetes | Flavobacteriia | 96.49 | 12.9 | 3239601 | 299 | Ichthyophthirius multifiliis strain G5 mitochondrion, complete genome |
| bin_91 | Bacteria | Proteobacteria | Gammaproteobacteria | 53.83 | 0 | 1402535 | 373 | no_ssu |
| bin_910 | Bacteria | Actinobacteria | Actinobacteria | 4.17 | 0 | 258215 | 65 | no_ssu |
| bin_911 | Bacteria | Firmicutes | Bacilli | 19.15 | 0.18 | 539209 | 177 | no_ssu |
| bin_912 | Bacteria | Cyanobacteria | NA | 89.66 | 62.25 | 3022814 | 523 | no_ssu |
| bin_913 | Bacteria | Proteobacteria | Gammaproteobacteria | 76.59 | 22.94 | 2505948 | 526 | Uncultured bacterium clone ASS_B1 16S ribosomal RNA gene and 16S-23S ribosomal RNA intergenic spacer, partial sequence |
| bin_914 | Bacteria | Proteobacteria | Gammaproteobacteria | 4.45 | 0.5 | 479136 | 75 | no_ssu |
| bin_915 | Bacteria | Cyanobacteria | NA | 23.43 | 0 | 626716 | 217 | no_ssu |
| bin_916 | Bacteria | Actinobacteria | Actinobacteria | 88.28 | 0 | 2034357 | 286 | Uncultured bacterium clone Contig\98 16S ribosomal RNA gene, partial sequence |
| bin_917 | Bacteria | Bacteroidetes | Flavobacteriia | 0 | 0 | 548374 | 132 | no_ssu |
| bin_918 | Bacteria | Proteobacteria | Alphaproteobacteria | 6 | 0.86 | 526164 | 92 | no_ssu |
| bin_919 | Bacteria | Proteobacteria | Gammaproteobacteria | 20.9 | 0 | 354356 | 128 | no_ssu |
| bin_92 | Bacteria | Actinobacteria | Actinobacteria | 0 | 0 | 908668 | 220 | no_ssu |
| bin_920 | Bacteria | Firmicutes | Bacilli | 5.44 | 2.34 | 520667 | 39 | no_ssu |
| bin_921 | Bacteria | Proteobacteria | Alphaproteobacteria | 8.62 | 1.72 | 1525834 | 556 | no_ssu |
| bin_922 | Bacteria | Firmicutes | Bacilli | 5.38 | 0.58 | 523214 | 56 | no_ssu |
| bin_923 | Bacteria | Actinobacteria | Actinobacteria | 1.15 | 0 | 765918 | 181 | no_ssu |
| bin_924 | Bacteria | Proteobacteria | Gammaproteobacteria | 93.42 | 1.72 | 4658137 | 339 | Bacterium enrichment culture clone B161(2011) 16S ribosomal RNA gene, partial sequence |
| bin_925 | Bacteria | Proteobacteria | Gammaproteobacteria | 93.53 | 14.1 | 3103077 | 367 | no_ssu |
| bin_926 | Bacteria | Actinobacteria | Actinobacteria | 54.1 | 2.7 | 1253148 | 375 | no_ssu |
| bin_927 | Bacteria | Proteobacteria | Alphaproteobacteria | 72.45 | 3.6 | 2521620 | 572 | Uncultured bacterium clone DWI03F 16S ribosomal RNA gene, partial sequence |
| bin_928 | Bacteria | Proteobacteria | Gammaproteobacteria | 66.54 | 6.9 | 2405020 | 580 | no_ssu |
| bin_929 | Bacteria | Firmicutes | Clostridia | 4.91 | 0.81 | 431618 | 41 | no_ssu |
| bin_93 | Bacteria | Bacteroidetes | Flavobacteriia | 18.53 | 0 | 530994 | 195 | no_ssu |
| bin_930 | Bacteria | Firmicutes | Bacilli | 71.54 | 6.18 | 1121581 | 117 | Uncultured organism clone SBXZ_4236 16S ribosomal RNA gene, partial sequence |
| bin_931 | Bacteria | Proteobacteria | Gammaproteobacteria | 8.33 | 0 | 992879 | 354 | no_ssu |
| bin_932 | Bacteria | Firmicutes | Clostridia | 71.06 | 2.8 | 408338 | 65 | no_ssu |
| bin_933 | Bacteria | Firmicutes | Clostridia | 4.92 | 0.43 | 428150 | 74 | no_ssu |
| bin_934 | Bacteria | Cyanobacteria | NA | 97.15 | 1.36 | 4417960 | 265 | no_ssu |
| bin_935 | Bacteria | Proteobacteria | Alphaproteobacteria | 71.91 | 1.2 | 1550188 | 380 | no_ssu |
| bin_936 | Bacteria | Firmicutes | Bacilli | 94.94 | 1.12 | 1004490 | 67 | Uncultured bacterium clone ESBB1AH06 16S ribosomal RNA gene, partial sequence |
| bin_937 | Bacteria | Firmicutes | Bacilli | 0 | 0 | 214889 | 28 | no_ssu |
| bin_938 | Bacteria | Firmicutes | Bacilli | 5.78 | 0.5 | 476157 | 79 | no_ssu |
| bin_939 | Bacteria | Proteobacteria | Gammaproteobacteria | 0 | 0 | 280663 | 36 | no_ssu |
| bin_94 | Bacteria | Cyanobacteria | NA | 39.68 | 1.72 | 1478035 | 335 | no_ssu |
| bin_940 | Bacteria | Proteobacteria | Gammaproteobacteria | 56.84 | 1.06 | 1802692 | 522 | no_ssu |
| bin_941 | Bacteria | Firmicutes | Bacilli | 9.51 | 0 | 913127 | 77 | no_ssu |
| bin_942 | Bacteria | Bacteroidetes | Flavobacteriia | 0 | 0 | 487578 | 88 | no_ssu |
| bin_943 | Bacteria | Bacteroidetes | Flavobacteriia | 84.47 | 6.43 | 2329675 | 367 | no_ssu |
| bin_944 | Bacteria | Bacteroidetes | Flavobacteriia | 4.31 | 0 | 335796 | 116 | no_ssu |
| bin_945 | Bacteria | Firmicutes | Bacilli | 4.91 | 0.86 | 421376 | 31 | no_ssu |
| bin_946 | Bacteria | Proteobacteria | Betaproteobacteria | 31.9 | 0.86 | 746905 | 220 | no_ssu |
| bin_947 | Bacteria | Proteobacteria | Gammaproteobacteria | 4.17 | 0 | 501995 | 64 | no_ssu |
| bin_948 | Bacteria | Proteobacteria | Alphaproteobacteria | 28.89 | 0 | 1037379 | 275 | no_ssu |
| bin_949 | Bacteria | Bacteroidetes | Cytophagia | 12.5 | 4.17 | 386331 | 116 | no_ssu |
| bin_95 | Bacteria | Firmicutes | Bacilli | 4.47 | 0.45 | 438041 | 64 | no_ssu |
| bin_950 | Bacteria | Actinobacteria | Actinobacteria | 51.22 | 2.93 | 1860641 | 581 | Leptocylindrus danicus chloroplast, complete genome |
| bin_951 | Bacteria | Proteobacteria | Betaproteobacteria | 71.72 | 3.45 | 1399621 | 150 | no_ssu |
| bin_952 | Bacteria | Firmicutes | Bacilli | 4.91 | 0.5 | 455036 | 20 | no_ssu |
| bin_953 | Bacteria | Proteobacteria | Gammaproteobacteria | 64.46 | 1.48 | 1514546 | 367 | Thalassolituus sp. strain C2-1 16S ribosomal RNA gene, partial sequence |
| bin_954 | Bacteria | Bacteroidetes | Flavobacteriia | 0 | 0 | 349541 | 32 | no_ssu |
| bin_955 | Bacteria | Bacteroidetes | Flavobacteriia | 0 | 0 | 485115 | 82 | no_ssu |
| bin_956 | Bacteria | Proteobacteria | Gammaproteobacteria | 50.08 | 1.72 | 2050861 | 673 | no_ssu |
| bin_957 | Bacteria | Bacteroidetes | Flavobacteriia | 92.41 | 1.65 | 3144341 | 329 | no_ssu |
| bin_958 | Bacteria | Firmicutes | Bacilli | 84.48 | 11.79 | 1087181 | 183 | Candidatus Actinomarina minuta clone MedDCM-OCT-S40-C95 genomic sequence |
| bin_959 | Bacteria | Firmicutes | Bacilli | 4.7 | 0.5 | 407836 | 30 | no_ssu |
| bin_96 | Archaea | Thaumarchaeota | NA | 62.91 | 33.98 | 1327060 | 352 | Uncultured bacterium JM9_G5 genomic sequence |
| bin_960 | Bacteria | Firmicutes | Clostridia | 1.29 | 0 | 245091 | 64 | no_ssu |
| bin_961 | Bacteria | Cyanobacteria | NA | 8.62 | 0 | 291493 | 105 | no_ssu |
| bin_962 | Bacteria | Proteobacteria | Alphaproteobacteria | 82.77 | 5.07 | 2209084 | 384 | no_ssu |
| bin_963 | Bacteria | Proteobacteria | Gammaproteobacteria | 0 | 0 | 1194487 | 314 | no_ssu |
| bin_964 | Bacteria | Bacteroidetes | Flavobacteriia | 49.45 | 0.48 | 1486888 | 454 | no_ssu |
| bin_965 | Bacteria | Cyanobacteria | NA | 1.72 | 0 | 382487 | 138 | no_ssu |
| bin_966 | Bacteria | Proteobacteria | Gammaproteobacteria | 80.05 | 0.75 | 2581472 | 375 | no_ssu |
| bin_967 | Bacteria | Proteobacteria | Alphaproteobacteria | 7.21 | 0 | 491506 | 197 | no_ssu |
| bin_968 | Bacteria | Proteobacteria | Alphaproteobacteria | 39.23 | 9.32 | 2652968 | 978 | no_ssu |
| bin_969 | Archaea | Thaumarchaeota | NA | 0 | 0 | 766660 | 73 | no_ssu |
| bin_97 | Bacteria | Proteobacteria | Gammaproteobacteria | 74.72 | 18.19 | 1643213 | 436 | no_ssu |
| bin_970 | Bacteria | Proteobacteria | Gammaproteobacteria | 0 | 0 | 656946 | 126 | no_ssu |
| bin_971 | Bacteria | Proteobacteria | Gammaproteobacteria | 0 | 0 | 434363 | 92 | no_ssu |
| bin_972 | Bacteria | Cyanobacteria | NA | 12.07 | 0 | 393904 | 129 | no_ssu |
| bin_973 | Bacteria | Proteobacteria | Gammaproteobacteria | 6.25 | 0 | 668795 | 227 | no_ssu |
| bin_974 | Bacteria | Proteobacteria | Gammaproteobacteria | 73.83 | 4.84 | 3248802 | 757 | no_ssu |
| bin_975 | Bacteria | Actinobacteria | Actinobacteria | 0 | 0 | 229935 | 69 | no_ssu |
| bin_976 | Bacteria | Firmicutes | Bacilli | 4.57 | 0 | 249251 | 93 | no_ssu |
| bin_977 | Bacteria | Proteobacteria | Alphaproteobacteria | 72.92 | 11.21 | 2170658 | 387 | no_ssu |
| bin_978 | Bacteria | Proteobacteria | Alphaproteobacteria | 22.95 | 0 | 304074 | 80 | no_ssu |
| bin_979 | Bacteria | Proteobacteria | Gammaproteobacteria | 16.91 | 6.17 | 12724058 | 4059 | Uncultured bacterium gene for 16S ribosomal RNA, partial sequence, clone: f86_1 |
| bin_98 | Bacteria | Cyanobacteria | NA | 96.82 | 0.71 | 2793981 | 249 | Uncultured bacterium clone DP10.2.55 16S ribosomal RNA gene, partial sequence |
| bin_980 | Bacteria | Firmicutes | Bacilli | 6.17 | 0.5 | 420029 | 26 | no_ssu |
| bin_981 | Bacteria | Nitrospirae | Nitrospira | 85.53 | 5.24 | 2760392 | 494 | no_ssu |
| bin_982 | Viruses | NA | NA | 0 | 0 | 397575 | 74 | no_ssu |
| bin_983 | Bacteria | Cyanobacteria | NA | 61.47 | 6.66 | 1634642 | 420 | no_ssu |
| bin_984 | Bacteria | Bacteroidetes | Flavobacteriia | 1.65 | 0 | 427281 | 73 | Uncultured bacterium clone PS200 16S ribosomal RNA gene, partial sequence |
| bin_985 | Bacteria | Proteobacteria | Deltaproteobacteria | 0 | 0 | 206748 | 40 | no_ssu |
| bin_986 | Bacteria | Proteobacteria | Gammaproteobacteria | 2.14 | 0.07 | 487043 | 106 | no_ssu |
| bin_987 | Bacteria | Proteobacteria | Betaproteobacteria | 4.17 | 0 | 422038 | 161 | no_ssu |
| bin_988 | Bacteria | Proteobacteria | Betaproteobacteria | 0 | 0 | 428136 | 157 | no_ssu |
| bin_989 | Bacteria | Proteobacteria | Betaproteobacteria | 29.62 | 0 | 369171 | 112 | no_ssu |
| bin_99 | Bacteria | Proteobacteria | Gammaproteobacteria | 30.01 | 0 | 522616 | 182 | no_ssu |
| bin_990 | Bacteria | Actinobacteria | Actinobacteria | 51.68 | 8.55 | 1014506 | 266 | no_ssu |
| bin_991 | Bacteria | Firmicutes | Bacilli | 0 | 0 | 284312 | 39 | no_ssu |
| bin_992 | Bacteria | Verrucomicrobia | Opitutae | 85.35 | 1.37 | 2246221 | 364 | Uncultured Opitutus sp. clone PBL2-50 16S ribosomal RNA gene, partial sequence |
| bin_993 | Bacteria | Proteobacteria | Betaproteobacteria | 1.65 | 0.43 | 299171 | 46 | no_ssu |
| bin_994 | Bacteria | Actinobacteria | Actinobacteria | 61.01 | 7.63 | 1033062 | 243 | no_ssu |
| bin_995 | Bacteria | Firmicutes | Bacilli | 3.01 | 0.47 | 422071 | 104 | no_ssu |
| bin_996 | Bacteria | Proteobacteria | Alphaproteobacteria | 8.62 | 0 | 399596 | 130 | no_ssu |
| bin_997 | Bacteria | Proteobacteria | Gammaproteobacteria | 4.01 | 0.29 | 372024 | 66 | no_ssu |
| bin_998 | Bacteria | Actinobacteria | Actinobacteria | 53.71 | 39.66 | 1992633 | 595 | no_ssu |
| bin_999 | Bacteria | Firmicutes | Clostridia | 4.13 | 1.44 | 403730 | 71 | no_ssu |
